# Design of Single‐Atom Catalysts Anchored in N‐Doped Biphenylene Using Symbolic Regression for Electrocatalytic Nitrate Reduction to Ammonia

**DOI:** 10.1002/advs.202512651

**Published:** 2026-01-08

**Authors:** Zheng Shu, Zhangsheng Shi, Huaxian Jia, Huifang Xu, Zian Xu, Zhongheng Li, Man‐Fai Ng, Teck Leong Tan, Fuqiang Huang, Yongqing Cai

**Affiliations:** ^1^ Joint Key Laboratory of the Ministry of Education, Institute of Applied Physics and Materials Engineering University of Macau Macau Macau SAR 999078 China; ^2^ State Key Laboratory of Metal Matrix Composites, School of Materials Science and Engineering Shanghai Jiao Tong University Shanghai 200240 China; ^3^ Department of Chemistry City University of Hong Kong Hong Kong Hong Kong SAR 999077 China; ^4^ Tencent, AI for Life Sciences Lab Shenzhen 518057 China; ^5^ Department of Materials Science and Engineering Southern University of Science and Technology Shenzhen 518055 China; ^6^ Institute of High Performance Computing (IHPC) Agency for Science, Technology and Research (A∗STAR) 1 Fusionopolis Way, #16‐16 Connexis Singapore 138632 Singapore

**Keywords:** ammonia production, catalysts design, nitrate reduction, symbolic regression

## Abstract

Electrocatalytic nitrate reduction (NO_3_RR) presents a synergistic strategy, achieving dual benefits in energy transformation and environmental remediation through a single process. However, the complexity of its reaction pathway and the lack of descriptors impede the rational design of high‐performance NO_3_RR electrocatalysts. Herein, employing a series of transition metal doped and nitrogen decorated biphenylene network (TM‐C_x_N_y_@BPN), an inclusive recipe toward the stability, reaction mechanism, and activity trend of single atomic catalysts (SACs) for NO_3_RR is proposed by integrating multidimensional insights from coordination environment, thermodynamic and electrochemical stability, Gibbs free energy profiles, electronic properties, and activity descriptors. It is revealed that the NO_3_RR performance of SACs is highly correlated with their local environments. Furthermore, the hybridization between the TM‐3*d* orbitals and 2π* orbitals of NO_3_^−^ gives rise to the formation of *d*‐π* orbitals, thus promoting the NO_3_^−^ activation. A symbolic regression is designed to capture the hidden descriptors of NO_3_RR, outperforming than using single adsorbate or electronic descriptors for hyper dimensional system entailing large geometric variability.

## Graphical abstract

A high‐throughput in silico calculations for NO_3_RR based on transition metal doped and nitrogen decorated biphenylene network are performed. A symbolic regression is designed to capture the hidden descriptors of NO_3_RR for hyper dimensional system entailing large geometric variability. The study sheds light on a new design principle for developing SACs supported on carbon materials with excellent performance toward NO_3_RR.

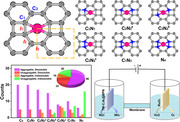

## 1 Introduction

With the rapid industrial developments and human activities, excessive nitrate (NO_3_^−^) accumulation may destroy the global nitrogen equilibrium.^[^ ^1^, ^2^, ^3^ ^]^ NO_3_^−^ is one of the major water pollutants mainly from industrial wastewater and domestic sewage. Continuous NO_3_^−^ accumulation in water bodies could disrupt the global nitrogen cycle equilibrium, posing a major hazard to human health and ecosystem.^[^ ^4^, ^5^ ^]^ Therefore, NO_3_^−^ remediation for reducing it to harmless products provides an appealing perspective for alleviating the environmental concerns. In recent years, the electrocatalytic nitrate reduction (NO_3_RR) has been considered as a “two birds, one stone” approach for the denitrification of wastewater, while simultaneously producing value‐added ammonia (NH_3_) under ambient conditions.^[^ ^6^, ^7^, ^8^ ^]^ NH_3_ is a desired product as the building block for the production of nitrogen fertilizers and pharmaceuticals, which is mainly dependent on the traditional Haber‐Bosch process under harsh condition of high temperature and high pressure.^[^ ^9^, ^10^ ^]^ The cathodic NO_3_RR is one of the most promising way to solve both the environmental remediation and energy transformation without massive fossil fuels consumption and huge green gases emission. Furthermore, compared with the other way via nitrogen (N_2_) reduction for obtaining NH_3_, NO_3_^−^ is very soluble in water to accelerate reaction kinetics, and the dissociation energy of N═O bond (204 kJ mol^−1^) is much lower than that of N≡N bond (941 kJ mol^−1^).^[^ ^11^, ^12^ ^]^ Nevertheless, the lack of highly active, stable and selective electrocatalysts hinders its widespread usage.

Intrinsically, the electrochemical reduction for NO_3_^−^ to NH_3_ is a complicated chemical process, involving a transfer of eight electrons (*e* ^−^) and nine protons (H^+^) by a series of consecutive hydrogenation and dehydration steps: NO_3_^−^ + 9H^+^ + 8*e* ^−^ → NH_3_ + 3H_2_O, *E*_0_ = 0.82 V.^[^ ^13^, ^14^, ^15^ ^]^ Moreover, these proton‐coupled electron transfer (PCET) steps are accompanied by multiple side reactions, which can generate many byproducts (e.g., NO_2_, NO, N_2_O, and N_2_). Among various metal catalysts, copper (Cu) surface has shown high activity and high selectivity as electrochemical catalyst for NO_3_RR due to the similarity of LUMO π* orbitals between NO_3_^−^ and Cu atoms.^[^ ^16^, ^17^, ^18^, ^19^ ^]^ Therefore, the design of Cu‐based materials has received much interests due to their low cost and relatively high activity.^[^ ^20^, ^21^ ^]^ For example, Zhou et al. reported that the incorporation of I single atoms on Cu (100) could achieve exceptional NO_3_RR activity with a high Faradaic efficiency of 98.5% under neutral conditions.^[^ ^20^ ^]^ Bai et al. unveiled the fundamental evolution process and active species using Cu_2_O nanocubes at different potentials and reaction times, thus promoting the understanding of underlying roles and reaction mechanisms of different Cu species.^[^ ^21^ ^]^ However, the poor stability, large limiting potential and NO_3_^−^ accumulation inhibit the development of Cu‐based electrocatalysts.

Since the concept of “single‐atom catalyst” (SAC) was proposed in 2011,^[^ ^22^ ^]^ much attention has been paid to this frontier area.^[^ ^23^, ^24^, ^25^, ^26^, ^27^, ^28^, ^29^, ^30^ ^]^ By tuning the coordination environments and atomic types of active centers, the maximum atomic utilization, the quantum effect and the best catalytic activity can be realized.^[^ ^23^, ^24^ ^]^ Furthermore, SACs could circumvent the undesired N–N coupling step in NO_3_RR due to the lack of neighboring active sites, which promotes the formation of NH_3_ and hinders the selectivity toward N_2_.^[^ ^12^, ^25^ ^]^ In the past few years, the so‐called transition metal anchored nitrogen and carbon (TM‐N‐C)‐based SACs have been considered as the promising electrocatalysts for NO_3_RR.^[^ ^25^, ^26^, ^27^, ^28^, ^29^, ^30^ ^]^ Niu et al. investigated the feasibility of SACs for NO_3_RR on graphitic carbon nitrides (g‐CN) with revealing the limiting potential by the adsorption strength of NO_3_^−^. In particular, Gu et al. experimentally designed Cu‐N_1_O_2_ SACs with coordination desymmetrization to achieve ≈96.5% NO_3_‐to‐NH_3_ conversion efficiency.^[^ ^29^ ^]^ Murphy et al. synthesized a rich set of TM‐N‐C‐based SACs to reveal diverse NO_3_RR performance.^[^ ^30^ ^]^ They presented a simple computational descriptor to estimate the NO_3_RR activity of TM‐N‐C catalysts correlated with the experimental performance. However, the structure‐activity relationships for NO_3_RR remain largely underexplored because the catalytic activity varies greatly on different carbon supports. Rational design of optimal electrocatalysts still remains challenging due to the complexity of NO_3_RR. Limited understanding remains about activation processes and fast proton transfer within a restricted hyper dimensional space formed by the reactant, environment, catalyst and its support.

In this work, aiming to explore the elusive correlation among the energetics associated with NO_3_RR, we propose a symbolic regression to tackling the multivariant dependence of the activation processes. Taking the new 2D carbon allotrope^[^ ^31^, ^32^, ^33^, ^34^, ^35^, ^36^ ^]^—biphenylene network (BPN) as the platform, the NO_3_RR activities are explored across the diverse chemical landscape of transition metals (TM), encompassing up to 25 distinct TM species (Figure S1, Supporting Information) embedded in diverse N*_x_* C*_y_* coordination environments (denoted as TM‐C_x_N_y_@BPN) formed by nitrogen substitution. Employing large‐scale density functional theory (DFT) calculations, we construct a comprehensive framework mapping the thermodynamic and electrochemical stability, reaction pathways, catalytic activity, and selectivity of these SACs to elucidate the structure‐activity relationships underlying NO_3_RR. The symbolic regression is demonstrated to an effective method to find the hidden expressions for predicting NO_3_RR performance that is hard to describe by single quantities.

## 2 Results and Discussion

### 2.1 Screening Stable TM‐C_x_N_y_@BPN for NO_3_RR

**Figure 1:**
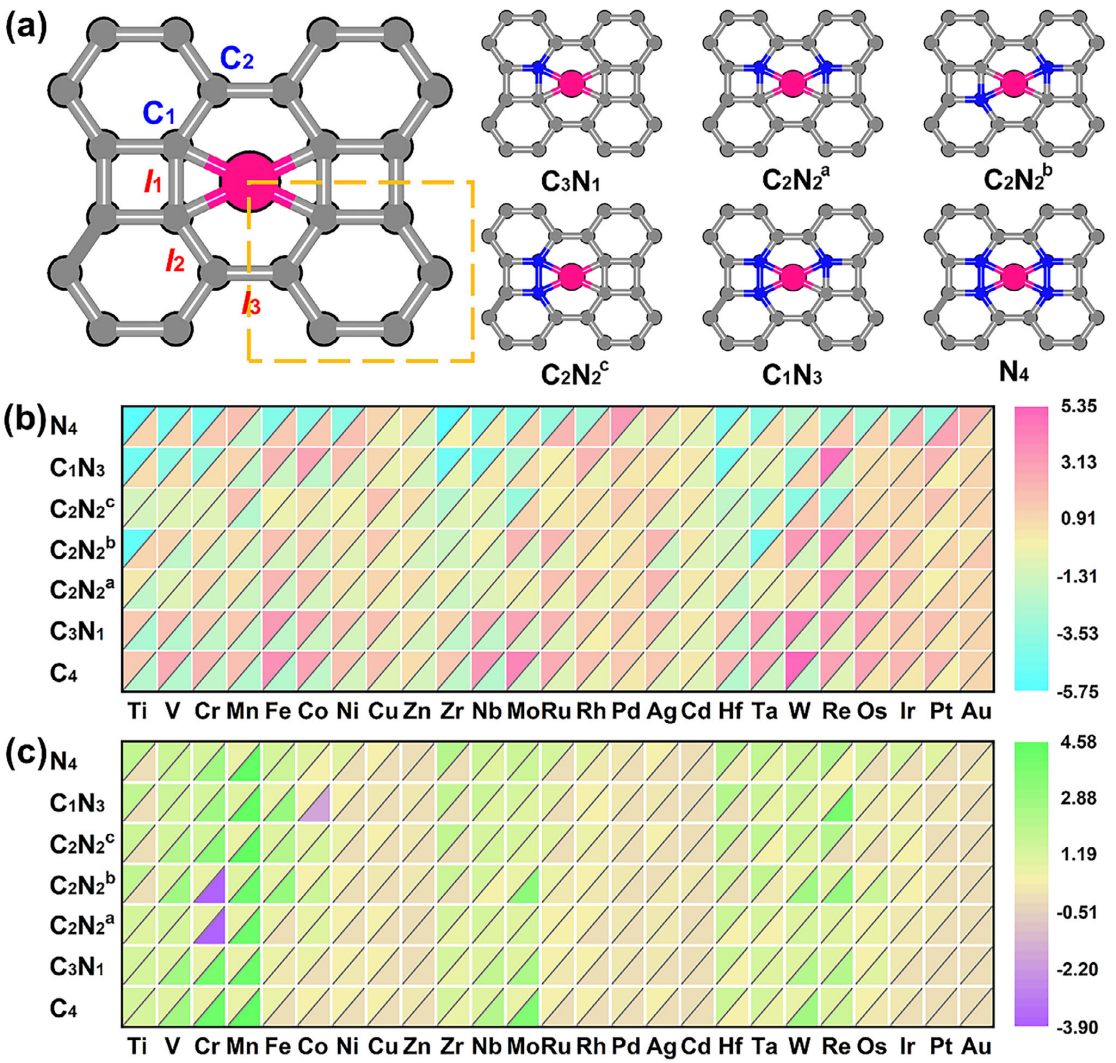
a) Schematic configurations of TM single atoms embedded in C*_x_* N*_y_* @BPN. The unit cell of BPN is indicated by the orange dash line. The grey, blue and pink balls represent C, N, and TM atoms, respectively. b) Double heatmap for the stabilities of TM‐C_x_N_y_@BPN. The data (with units of eV) located in the upper left corner represent formation energies (*E*_form_), while those located in the lower right corner represent dissolution potentials (*U*_diss_). The closer to blue the color is, the higher the thermodynamic stability of the candidates have. Conversely, the closer to red the color is, the higher the electrochemical stability of the candidates have. c) Double heatmap for charge transfer and spin states of active sites on TM‐C_x_N_y_@BPN. The data located in the upper left corner represent charge transfer (with units of e) of TM, while those located in the lower right corner represent the spin moment (with units of µ_B_) of TM.

**Figure** **1**a shows the atomic configuration of monolayer BPN, whose primitive cell contains six carbon atoms with a rectangular lattice (space group *Pmmm*). The lattice constants of monolayer BPN are *a* = 4.55 Å and *b* = 3.77 Å with an anisotropic *a*/*b* ratio of 1.21. As a non‐benzenoid carbon allotrope, BPN consists of octagonal, tetragonal, and hexagonal rings bonded by sp^2^ hybridization, causing slightly distinctive C─C bond lengths. As shown in Figure 1a, the lengths of *l*_1_, *l*_2_ and *l*_3_ C─C bonds are 1.45, 1.41, and 1.45 Å, respectively. The band structure and the density of state (DOS) of the pure BPN are shown in Figure S2 (Supporting Information). The pristine BPN exhibits a conductor‐like electronic structure with several energy bands across the Fermi level (*E*_F_), which is consistent with the experimental results.^[^ ^31^ ^]^ Even in narrow ribbons, BPN exhibits a metallic character, in contrast to graphene. In BPN, there are two nonequivalent C atoms, namely, C_1_ (4−6−8) with one four‐atom ring, one six‐atom ring and one eight‐atom ring, and C_2_ (6−8−8) with one six‐atom ring and two eight‐atom rings. The biggest hole in BPN formed by eight‐atom ring is used to anchor the central active TM atoms for constructing catalysts. In general, the local environment of active sites plays an essential role for its electronic structure and catalytic performance. As revealed by a previous report, the formation energy of vacancy of C_1_ atom is lower than that of C_2_ atom,^[^ ^35^ ^]^ implying that the C_1_ atom may be substituted more easily than the C_2_ atom. Hence, by replacing C_1_ atoms with 1‐4 N atoms, six different N*_x_* C*_y_* (*x* and *y* represent the number of N and C atoms eligible for bonding with TM atom respectively) coordination, namely C_3_N_1_, C_2_N_2_^a^, C_2_N_2_^b^, C_2_N_2_^c^, C_1_N_3,_ and N_4_, were considered to establish distinctive local coordination environments, as shown in Figure 1a. To confirm their thermal stabilities, these C_x_N_y_@BPN were simulated for 20 ps with a timestep of 2 fs at the constant temperature. As shown in Figure S3 (Supporting Information), the geometric structures of these C*_x_* N*_y_* @BPN remained stable, with their energy oscillating around the equilibrium value, thereby verifying their commendable thermodynamic stability. Furthermore, all C_x_N_y_@BPN structures exhibit metallic characteristics with energy bands crossing the *E*_F_ (see Figure S3, Supporting Information), implying the superior electron transport properties. Considering 25 *d*‐block TMs and 7 N*_x_* C*_y_* coordination environments, 175 different TM‐C_x_N_y_@BPN (see Figures S4–S10, Supporting Information) were generated to form a vast chemical space to exhaustively unravel the structure‐activity relationship of catalyzing NO_3_RR. Detailed lattice parameters of these TM‐C_x_N_y_@BPN can be found in the Tables S1–S7 (Supporting Information). As we know, carbon supports can affect the electron‐donating capability, thus changing the charged states and *d*‐band center of active sites. Compared to graphene and other planar sp^2^ hybridized carbon allotropes, BPN consists of periodically arranged four‐, six‐, and eight‐membered rings, giving richer chemical environments with different activities for various studies.^[^ ^31^ ^]^ Moreover, the characteristics of BPN compared to other carbon materials are further discussed in the Section 2.7.

The thermodynamic and electrochemical stabilities are the prerequisites for the utilization of electrocatalysts under operating conditions.^[^ ^10^, ^15^, ^34^ ^]^ The candidates with negative formation energy (*E*_form_) and positive dissolution potential (*U*_diss_) exhibit durability in acidic solution, which indicates the extent to which the metal atoms resist clustering and dissolution respectively. From Figure 1b, we clearly observe the stability profiles of these TM‐C_x_N_y_@BPN. Therefore, a stability trend chart with respect to coordination environments and atomic species of active sites was established. TM‐C*_x_* N*_y_* @BPN catalysts with *E*_f_ > 0 eV or *U*_diss_ < 0 are unstable and hence not suitable for NO_3_RR. In total, 30 TM‐C*_x_* N*_y_* @BPN with thermodynamic and electrochemical stabilities through stability screening were considered for further discussions. The specific values of *E*_f_ and *U*_diss_ of these catalysts are presented in Tables S1–S7 (Supporting Information). To further demonstrate the rationality of choosing the eight‐atom ring to anchor TM atoms, the formation energies of TM atoms on six‐atom ring and eight‐atom ring with N_4_ coordination environments (denoted as *E*_form‐N4@C6_ and *E*_form‐N4@C8_) were calculated. As shown in Table S8 and Figure S11 (Supporting Information), it can be found that the values of most *E*_form‐N4@C6_ are above 0, implying their instability. Therefore, it can be concluded that the eight‐atom ring in BPN is the center for adsorbing TM atoms. Furthermore, the charge transfer and spin magnetic moment were calculated by the Bader charge method to better understand the structural information of all TM‐C*_x_* N*_y_* @BPN, as shown in Figure 1c. Obviously, the electrons are transferred from TM atoms to C*_x_* N*_y_* @BPN supports for most cases, ranging from 0.03–2.16 |*e*|, which could promote the adsorption of NO_3_^−^ with negative charges. The detailed data of these quantities can also be found in Tables S1–S7 (Supporting Information). As shown in Figure S12 (Supporting Information), there is an obvious tendency that early (late) TMs lose more (less) electrons for all coordination environments.

### 2.2 Nitrate Adsorption and Selectivity

For NO_3_RR, the adsorption and activation of NO_3_^−^ are the key steps for the entire reduction reaction. The change of Gibbs free energy of NO_3_^−^ adsorption (Δ*G*_NO3*_) in aqueous solution can be quantitatively evaluated by using the thermodynamic cycle.^[^ ^10^ ^]^ The computational details and thermodynamic cycle diagram (see Figure S13, Supporting Information) are provided in the Supporting Information. Given that the solution environment was already incorporated through a 0.392 eV correction, the solvent effect was deliberately excluded from the Δ*G*_NO3*_ calculation to avoid double‐counting and ensure accurate energetic assessments. As illustrated in Figure S14 (Supporting Information), there are two possible NO_3_^−^ adsorption configurations on single metal centers, namely 1‐O and 2‐O patterns. The corresponding energies and TM‐O bond lengths are listed in Tables S9–S15 (Supporting Information). The majority of TM‐C*_x_* N*_y_* @BPN structures preferentially adsorb NO_3_^−^ via the 2‐O coordination motif, whereas a distinct minority—including Au‐C_4_@BPN, Au‐C_2_N_2_^a^@BPN, Au‐C_2_N_2_^b^@BPN, Au‐C_2_N_2_^c^@BPN, Au‐C_1_N_3_@BPN, Ni‐N_4_@BPN, and Pt‐N_4_@BPN—exhibit a thermodynamically favored 1‐O adsorption pattern. In addition, NO_3_^−^ can't stably absorb on some cases (i.e., all Zn‐C*_x_* N*_y_* @BPN, all Cd‐C_x_N_y_@BPN, Ag‐C_2_N_2_^a^@BPN, Ag‐C_2_N_2_^b^@BPN, Pd‐N_4_@BPN, and Au‐N_4_@BPN), thus these catalysts were ruled out in the following discussions of NO_3_^−^ activation and reduction. Considering the periodic trends of TMs, early TMs exhibit excessively strong NO_3_^−^ adsorption, whereas late TMs demonstrate notably weaker adsorption, as confirmed by the calculations shown in the Figure S15 (Supporting Information). This result is largely consistent with the charged states revealed by Figure S12 (Supporting Information).

**Figure 2:**
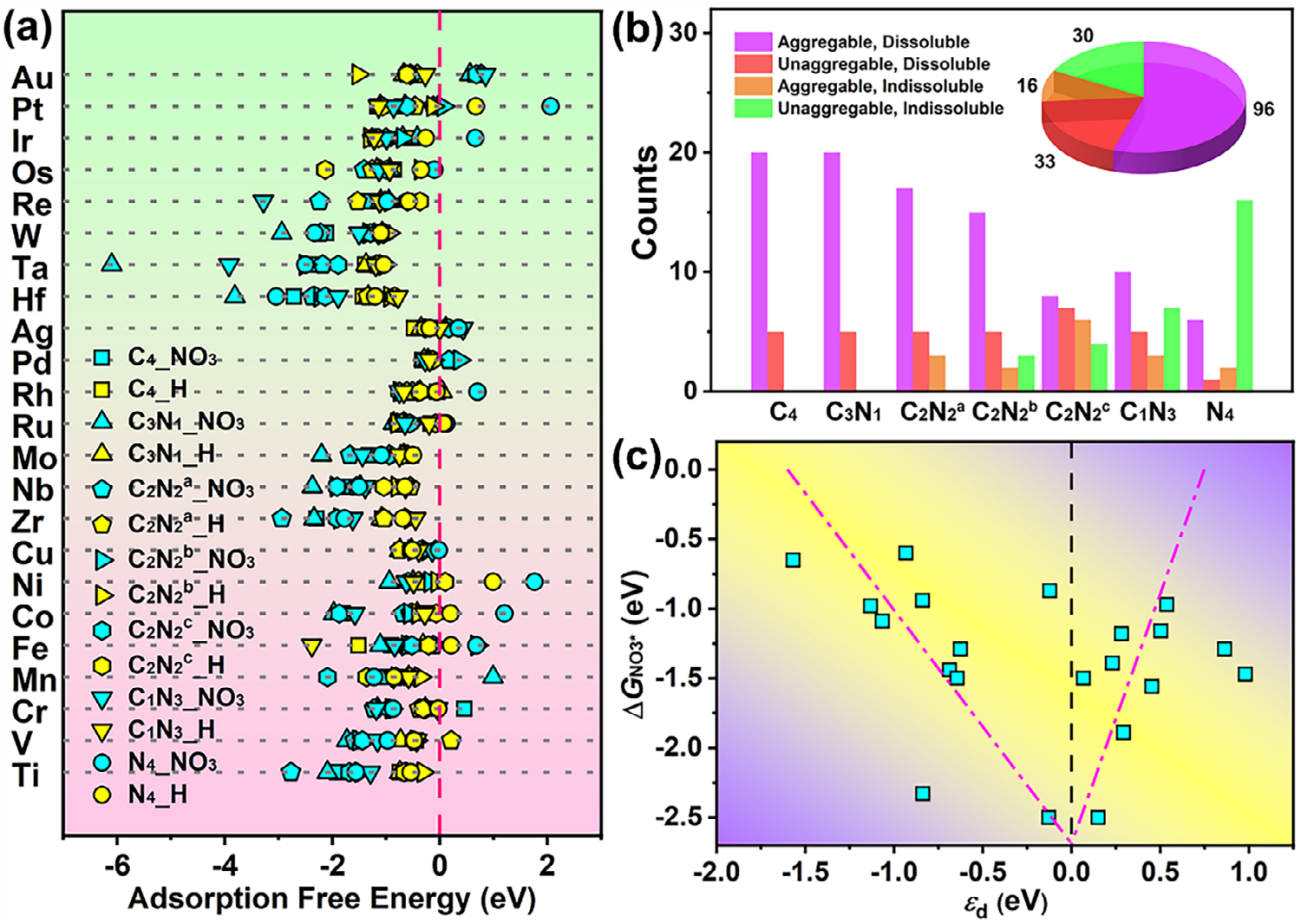
a) Comparison of adsorption free energies of NO_3_^−^ and atomic H on TM‐C*_x_* N*_y_* @BPN. b) Summary pie of unaggregable and indissoluble candidates. c) Volcano plot of *d*‐band center (*ε*_d_) versus adsorption free energies of NO_3_^−^ (Δ*G*_NO3*_).

On the other hand, hydrogen reduction reaction (HER) is the main competing reaction during electrochemical NO_3_RR process. When adsorption free energies of hydrogen (Δ*G*_H*_) is less than Δ*G*_NO3*_, the catalyst tends to preferably adsorb atomic H.^[^ ^14^, ^25^ ^]^ In other words, atomic H would first block the reaction site and make NO_3_^−^ adsorption difficult, thus reducing the Faraday efficiency of NH_3_ production. Therefore, a preliminary screening was carried out based on the adsorption selectivity of NO_3_^−^ versus atomic H. As displayed in **Figure** **2**a, the Δ*G*_NO3*_ and Δ*G*_H*_ on all TM‐C*_x_* N*_y_* @BPN are compiled to evaluate the preliminary selectivity, and their corresponding values are tabulated in Tables S9–S15 (Supporting Information). As shown in Figure S16 (Supporting Information), the region above (below) the red line represents NO_3_RR (HER) selective SACs. Those catalysts with Δ*G*_H*_ < Δ*G*_NO3*_ were excluded in further discussion. The bond lengths of TM‐O and TM‐H are provided in Tables S9–S15 (Supporting Information). The main reason that we selected all TM‐C*_x_* N*_y_* @BPN for H and NO_3_^−^ adsorption is to explore their trends with atomic number and periodicity, as revealed in Figures S15 and S16 (Supporting Information). We can observe that Δ*G*_NO3*_ linearly increases with the increasing Δ*G*_H*_ on TM‐N_4_@BPN (see Figure S16g, Supporting Information), because most cases of TM‐N_4_@BPN are thermodynamically and electrochemically stable. However, this tendency is less apparent on other TM‐C*_x_* N*_y_* @BPN due to the existence of amounts of unstable examples. Figure S17 (Supporting Information) marked those TM‐C*_x_* N*_y_* @BPN whose Δ*G*_NO3*_ − Δ*G*_H*_ <−0.20 eV to alleviate computational errors, and the cases below the dashed line are further recognized as selective candidates toward NO_3_RR. Herein, to investigate the trends of NO_3_^−^ adsorption on TM‐C*_x_* N*_y_* @BPN, the relationship between charge transfer from TM atoms to C*_x_* N*_y_* @BPN substrates (denoted as *δ*) and Δ*G*_NO3*_ is examined in Figure S18a (Supporting Information). There is no significant linear correlation between *δ* and Δ*G*_NO3*_ with a poor coefficient of determination (R^2^) of 0.29. In the case of Δ*G*_H*_, the value of R^2^ becomes smaller, as shown in Figure S18b (Supporting Information). Inspired by previous works,^[^ ^37^, ^38^ ^]^ the catalytic activity is highly correlated with the composition of near‐neighbor environment of the binding site, so a descriptor 𝜑 that considers the active sites together with the nearest atoms is designed to quantify varying coordination environment as

(1) $$\varphi =\theta_{d}\times \frac{\chi_{\mathrm{TM}}+n_{N}\times \chi_{N}+n_{C}\times \chi_{C}}{\chi_{O/H}}$$

where *θ*_d_ represents the number of *d* electrons, *χ*_TM_, *χ*_N_, *χ*_C_, and *χ*_O/H_ correspond to the electronegativity of TM, N, C, and O or H elements, *n*_N_ and *n*_C_ denote the number of nearest neighboring N and C atoms. Herein, by employing 𝜑 as a new descriptor, the relationship between 𝜑 and Δ*G*_NO3*_ was investigated as shown in Figure S18c (Supporting Information). The 𝜑 descriptor exhibits a stronger linear correlation (R^2^ = 0.59) than that of *δ* and Δ*G*_NO3*_. However, this descriptor does not adequately differentiate between distinct C_2_N_2_ coordination environments. For example, the values of Ti‐C_2_N_2_^a^@BPN, Ti‐C_2_N_2_^b^@BPN and Ti‐C_2_N_2_^c^@BPN are same (𝜑 = 9.02). Furthermore, 𝜑 and Δ*G*_H*_ weakly correlate with the R^2^ of 0.115, as shown in Figure S18d (Supporting Information). This phenomenon indicates that *δ* and 𝜑 can't describe the Δ*G*_NO3*_ and Δ*G*_H*_ well, because unstable catalytic sites may undergo significant movement after H and NO_3_^−^ adsorption and should be excluded in the subsequent calculations. Therefore, exploring new descriptors that can characterize adsorption and catalytic performance is desired, which will be discussed in the following section.

Figure 2b summarizes the catalyst stability against metal aggregation and metal dissolution in the TM‐C_x_N_y_@BPN systems. Based on the above screening, there are twenty‐one unaggregatable, indissoluble and selective candidates. According to the Sabatier principle, an ideal catalyst should interact with the intermediates moderately, because too weak or too strong binding strength can cause a large free energy barrier for the whole reaction. To reveal the relationship between Δ*G*_NO3*_ and the electronic features of single‐atom sites, *d*‐band center (*ε*_d_) of TM is examined. The connection of the Δ*G*_NO3*_ with the *ε*_d_ for these twenty‐one TM‐C_x_N_y_@BPN is shown in Figure 2c. The projected density of states (PDOS) onto *d*‐orbitals of TMs and corresponding *ε*_d_ values are shown in Figure S19 (Supporting Information). A volcano‐shaped plot is formed, where SACs with *ε*_d_ approaching the top of volcano have the stronger NO_3_^−^ adsorption ability.

### 2.3 NO_3_RR Mechanism and NH_3_ Selectivity

**Figure 3:**
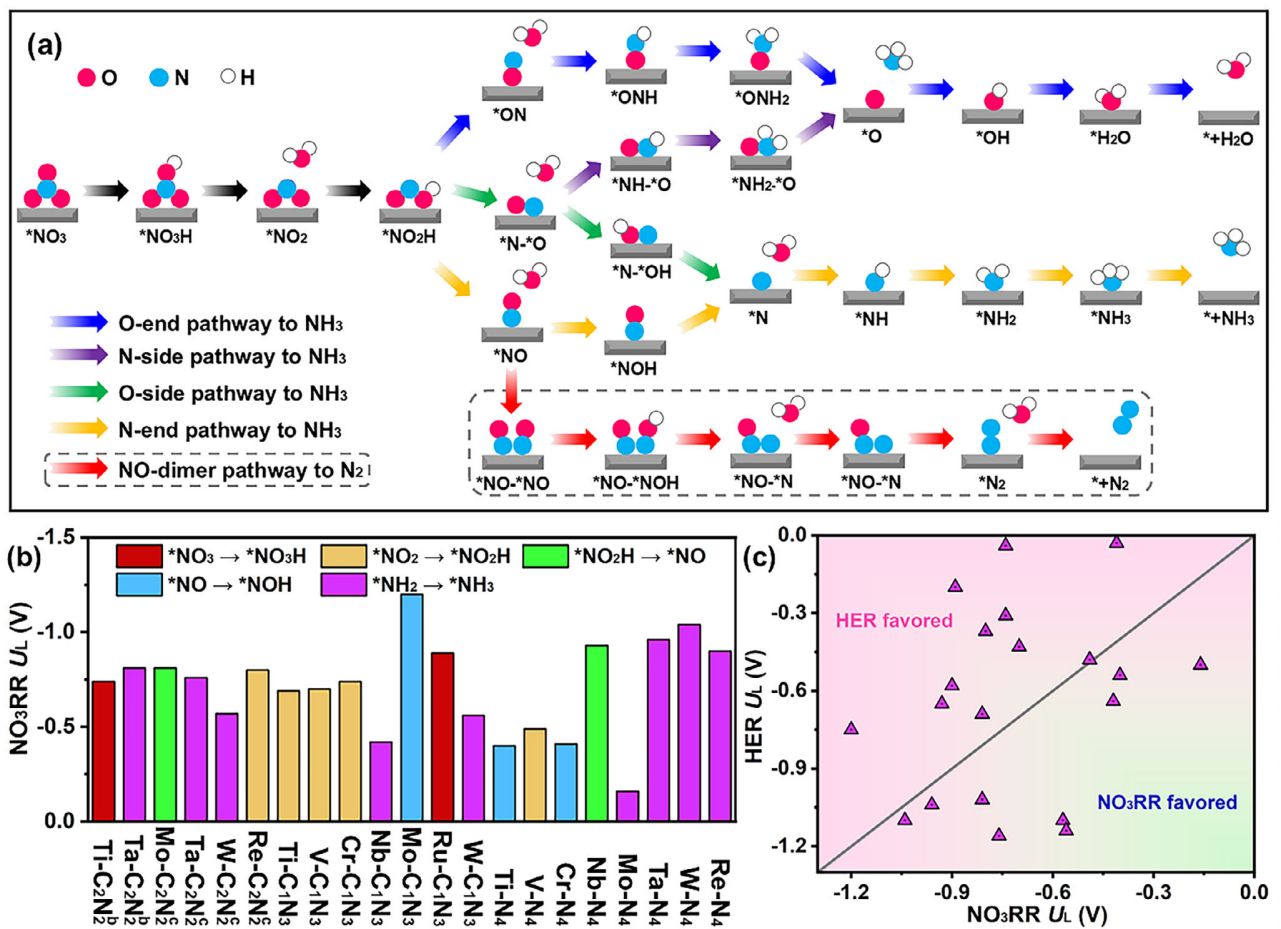
a) Various pathways of NO_3_RR, including O‐end, N‐end, O‐side, N‐side and NO‐dimer. b) Summary of limiting potentials on TM‐C*_x_* N*_y_* @BPN. c) Limiting potentials for NO_3_RR and HER illustrating the NO_3_RR selectivity of TM‐C*_x_* N*_y_* @BPN.

Prior to evaluating the activity performance of NO_3_RR, the structural stability of twenty‐one TM‐C_x_N_y_@BPN was assessed by AIMD simulations at room temperature over 20 ps (using a 2 fs time step) where the structural integrity is well maintained (see Figure S20, Supporting Information). Subsequently, the complete reaction pathways of these catalysts were calculated to elucidate the reaction mechanism. The reaction pathways for the NO_3_RR on SACs considered in this work are outlined in **Figure** **3**a, in line with previous theoretical studies.^[^ ^25^, ^26^ ^]^ The possible pathways of NO_3_RR could be categorized into four types according to the hydrogenation above the site of the *NO intermediate: O‐end, N‐end, O‐side and N‐side, as illustrated in Figure 3a. For the *NO intermediate, three primary configurations are identified (N‐end, O‐end and NO‐side, see Figure S21, Supporting Information), each leading to different hydrogenation pathways.

The changes of free energy of NO adsorption (Δ*G*_NO*_) associated with each configuration were calculated and shown in Table S16 (Supporting Information). The O‐end configuration exhibits a significantly higher ΔG value compared to N‐end and NO‐side configurations, indicating its thermodynamic infeasibility. Hence, the O‐end pathway was excluded in the following discussions. The N‐end pattern is the most stable *NO adsorption configuration on most substrates, while, for Ta‐C_2_N_2_^b^@BPN, Mo‐C_2_N_2_^c^@BPN, V‐N_4_@BPN, Nb‐N_4_@BPN, Ta‐N_4_@BPN, and W‐N_4_@BPN, the NO‐side pattern is more energetically favorable than the N‐end pattern. The potential‐limiting steps (PDS) can be evaluated by *U*_L_ = −Δ*G*_max_/e, where Δ*G*_max_ is the maximum Δ*G* among all elementary steps. The most favorable NO_3_RR pathway is identified by examining all possible intermediates with the lowest Δ*G*_max_ for the full steps in nitrate reduction pathways toward NH_3_. The values of *U*_L_ with their corresponding PDS for all TM‐C*_x_* N*_y_* @BPN are compared and shown in Figure 3b. Additionally, HER would inevitably dominate the whole reaction process if more negative *U*_L_ of NO_3_RR than that of HER. As shown in Figure 3c, the catalysts whose *U*_L_ of NO_3_RR is less negative than that of HER are favorable for NO_3_RR during the reaction process.

It is well recognized that Cu is the most active metal catalyst for NO_3_RR.^[^ ^16^, ^17^, ^18^, ^19^ ^]^ The Cu (100) surface is more favorable for the NO_3_RR than the Cu (111) surface,^[^ ^17^, ^18^, ^39^ ^]^ thus the NO_3_RR performance of Cu (100) is adopted as a benchmark for comparison. As shown in Figure S22a (Supporting Information), the NO_3_^−^ adsorption on Cu (100) surface is an exothermic process with an energy barrier of −0.16 eV, therefore, pristine Cu (100) surface has a weak ability to activate NO_3_^−^ ions. The vibrational frequencies, zero‐point energy and entropic contributions of intermediate species on Cu (100) surface at room temperature are calculated and listed in Table S17 (Supporting Information), and corresponding structures are shown in Figure S22b (Supporting Information). Unfortunately, the competing step of N‐N coupling for forming N_2_ is comparably easy to occur on Cu surfaces with a downhill energy barrier of −0.084 eV (see Figure S23a, Supporting Information), thereby suppressing the selectivity toward NH_3_. Since the distance between two adjacent metal active sites in SACs is relatively long and the interaction between them is small during the reaction, the possibility of NO‐dimer pathway for TM‐C_x_N_y_@BPN systems was excluded from consideration. To validate this suspect, in addition, the pathway involving the binding of NO as a dimer and subsequently generating N_2_ is also considered and depicted in the diagram of Ti‐N_4_@BPN and Mo‐N_4_@BPN, as shown in Figure S23b, c (Supporting Information). The N‐N coupling process involves high barriers up to 1.51 and 2.50 eV on Ti/N_4_@BPN and Mo/N_4_@BPN, respectively, which is much higher than that of Cu (100) surface.

**Figure 4:**
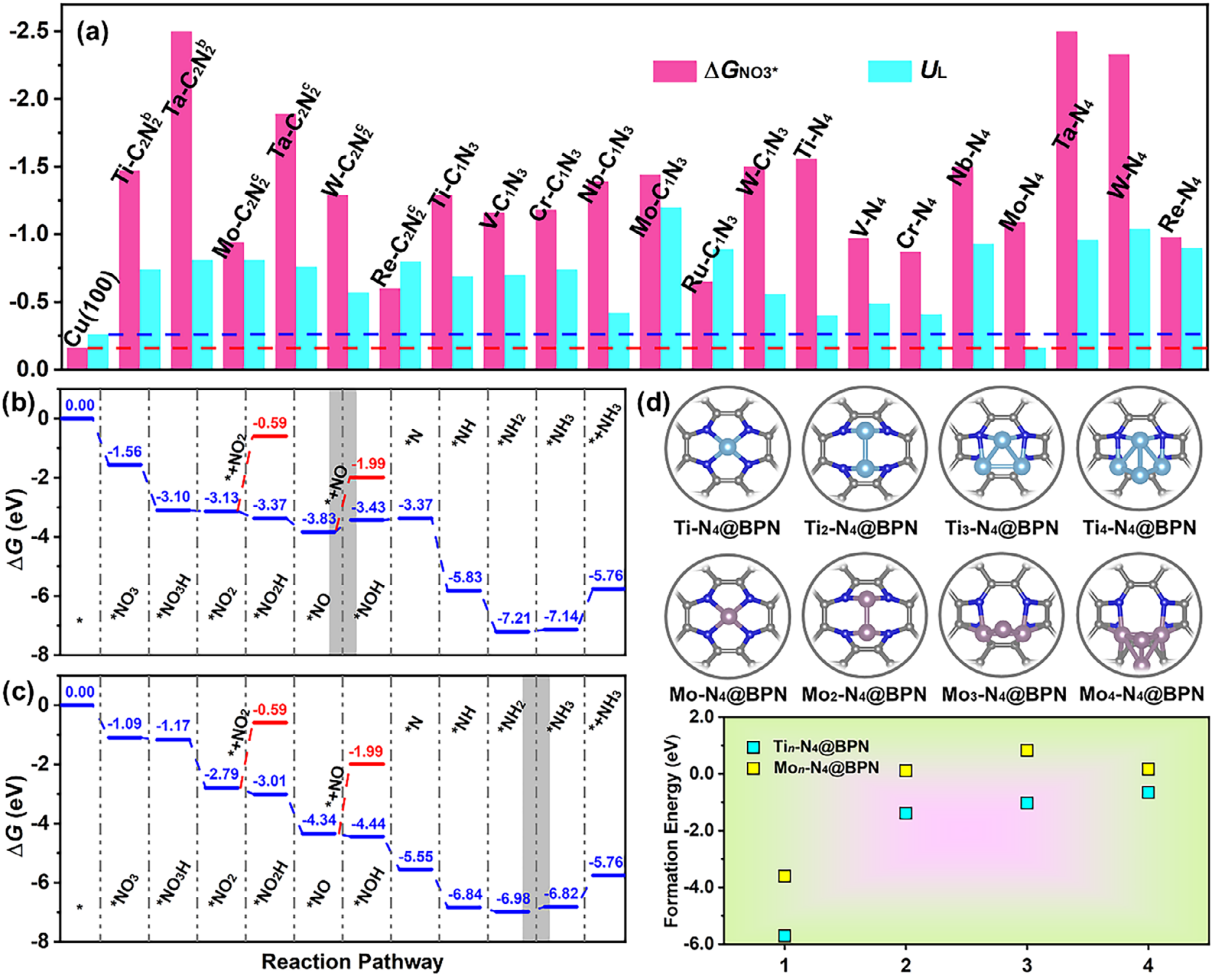
a) Comparison of adsorption free energies of NO_3_^−^ and limiting potentials on Cu (100) and TM‐C*_x_* N*_y_* @BPN. The unit of former is eV while that of later is V. Gibbs free energy profiles for NO_3_RR of b) Ti‐N_4_@BPN and c) Mo‐N_4_@BPN. d) Formation energies (*E*_form_) of Ti*_n_*‐N_4_@BPN and Mo*_n_*‐N_4_@BPN (*n* = 1‐4) and their corresponding structures.

Herein, activation via the N‐side pathway on Ta/C_2_N_2_^b^@BPN, Mo/C_2_N_2_^c^@BPN, V/N_4_@BPN, Nb/N_4_@BPN, Ta/N_4_@BPN, and W/N_4_@BPN is first investigated because the NO‐side configuration is more favorable than the N‐end configuration on these catalysts. However, the limiting *U*_L_ for NO_3_‐to‐NH_3_ on Ta/C_2_N_2_^b^@BPN, Mo/C_2_N_2_^c^@BPN, V/N_4_@BPN, Nb/N_4_@BPN, Ta/N_4_@BPN, and W/N_4_@BPN reaches down to −1.48, −1.54, −1.45, −1.32, −1.94, and −1.93 eV, respectively (see Figure S24, Supporting Information), indicating the difficulty associated to generating NH_3_ through the N‐side pathway. The specific values of each elementary steps can be found in Table S18 (Supporting Information). In contrast, the N‐end pathway is the most favorable mechanism for the systems we studied. As shown in **Figure** **4**a, a comparison of Δ*G*_NO3*_ and *U*_L_ for Cu (100) and all TM‐C_x_N_y_@BPN systems is presented. We can observe that all TM‐C_x_N_y_@BPN exhibit stronger adsorption of NO_3_^−^ than Cu (100). Among them, the Mo‐N_4_@BPN exhibits the highest *U*_L_ of −0.16 V to generate NH_3_, whereas Ti‐N_4_@BPN shows the next highest *U*_L_ of −0.40 V. For the Mo‐N_4_@BPN, its NO_3_RR performance exceeds the benchmark Cu (100) whose *U*_L_ is −0.26 V. The NO_3_RR free energy profiles for other TM‐C*_x_* N*_y_* @BPN are provided in the Figures S25–S27 (Supporting Information). The specific values of each elementary steps can be found in Table S19 (Supporting Information). Morever, the Nb‐C_1_N_3_@BPN, V‐N_4_@BPN and Cr‐N_4_@BPN are also predicted to be promising catalysts, with their respective *U*_L_ calculated at −0.42, −0.49, and −0.41 V, respectively.

### 2.4 NO_3_RR Performance of Ti‐N_4_@BPN and Mo‐N_4_@BPN

The ΔG for NO_3_^−^ adsorption decreases, whereas that for the competing hydrogen adsorption increases, with the applied potential. Therefore, those catalysts with *U*_L_ of NO_3_RR being lower than −0.50 V are still promising and thus selected for further exploration next regarding their NH_3_ selectivity. We find that, for Ti‐N_4_@BPN and Mo‐N_4_@BPN, the adsorption of NO_3_^−^ is comparatively stronger than that of hydrogen when potential reaches their respective *U*_L_ of NO_3_RR, as shown in Figure S28 (Supporting Information). In contrast, the Nb‐C_1_N_3_@BPN and V‐N_4_@BPN have weaker adsorption of NO_3_^−^ than that of hydrogen and the selectivity for NO_3_^−^ adsorption is inhibited under working potential. It should be noted that Cr‐N_4_@BPN is excluded from this discussion because it is HER favorable during the whole reaction process, as revealed in Figure 3c. Therefore, only Ti‐N_4_@BPN and Mo‐N_4_@BPN are examined to explore their NO_3_‐to‐NH_3_ performance below.

As shown in Figure 4b,c, the free energy profiles via N‐end pathway are presented. According to the free energy profiles via N‐end pathway, the NO_3_^−^ is stably adsorbed with two TM‐O bonds through an exothermic process with energy change of −1.56 eV for Ti‐N_4_@BPN and −1.09 eV for Mo‐N_4_@BPN. The PDS of Ti‐N_4_@BPN occurs in the *NO → *NOH, while the step of *NH_2_ → *NH_3_ is the PDS for Mo‐N_4_@BPN. The corresponding structures of each intermediate are shown in Figure S29 (Supporting Information). With the continuous supply of H^+^/*e* ^−^ pairs, successive hydrogenation with the O atoms of the adsorbed NO_3_^−^ induces the formation of NO* intermediate, with the Δ*G* of −1.54, −0.03, −0.24, and −0.46 eV for Ti‐N_4_@BPN, and −0.08, −1.62, −0.22, and −1.33 eV for Mo‐N_4_@BPN (Figure 4b,c). In the following step, NO* is hydrogenated by H^+^/*e* ^−^ pair to form NOH* intermediate, which is identified as the PDS for Ti‐N_4_@BPN with a small Δ*G* of 0.40 eV. In contrast, the step is exothermic with Δ*G* of −0.10 eV for Mo‐N_4_@BPN. Subsequently, NOH* is attacked by H^+^/*e* ^−^ pair to release H_2_O and form N* intermediate with Δ*G* of +0.06 eV for Ti‐N_4_@BPN and −1.11 eV for Mo‐N_4_@BPN. Eventually, *N species are readily hydrogenated to the formation of *NH, *NH_2_ and *NH_3_ and the desorption of NH_3_. For Ti‐N_4_@BPN, this process is energetically downhill until NH_3_ release. Interestingly, no energy barriers is found in the process from *NO_3_ to *NO, indicating that the electroreduction of NO_3_‐to‐NO is completely spontaneous on Ti‐N_4_@BPN and Mo‐N_4_@BPN. In addition, the possibilities to the formation of NO_2_, NO and N_2_ are investigated to validate their selectivity of NO_3_RR toward NH_3_. As plotted in Figure 4b,c, the energy barriers for the release of NO_2_ and NO reach up to 2.54 and 1.84 eV on Ti‐N_4_@BPN and 2.20 and 2.35 eV on Mo‐N_4_@BPN, implying that it is difficult to release these byproducts.

The formation possibilities of TM clusters on Ti‐N_4_@BPN and Mo‐N_4_@BPN are explored. Here, the values of *E*_form_ for Ti*_n_*‐N_4_@BPN and Mo*_n_*‐N_4_@BPN (*n* = 1–4) were calculated to evaluate the formation possibilities of TM clusters on BPN. The optimized structures and *E*_form_ values of Ti*_n_*‐N_4_@BPN and Mo*_n_*‐N_4_@BPN are shown in Figure 4d. The calculation results indicate that the formation of Ti and Mo clusters on N_4_@BPN is unfavorable because *E*_form_ values get higher with the increased size of clusters. Based on above discussions, it can be concluded that Ti‐N_4_@BPN and Mo*_n_*‐N_4_@BPN exhibit thermodynamic stability for NO_3_RR toward NH_3_.

### 2.5 Bonding Nature of Ti‐N_4_@BPN and Mo‐N_4_@BPN for NO_3_RR

**Figure 5:**
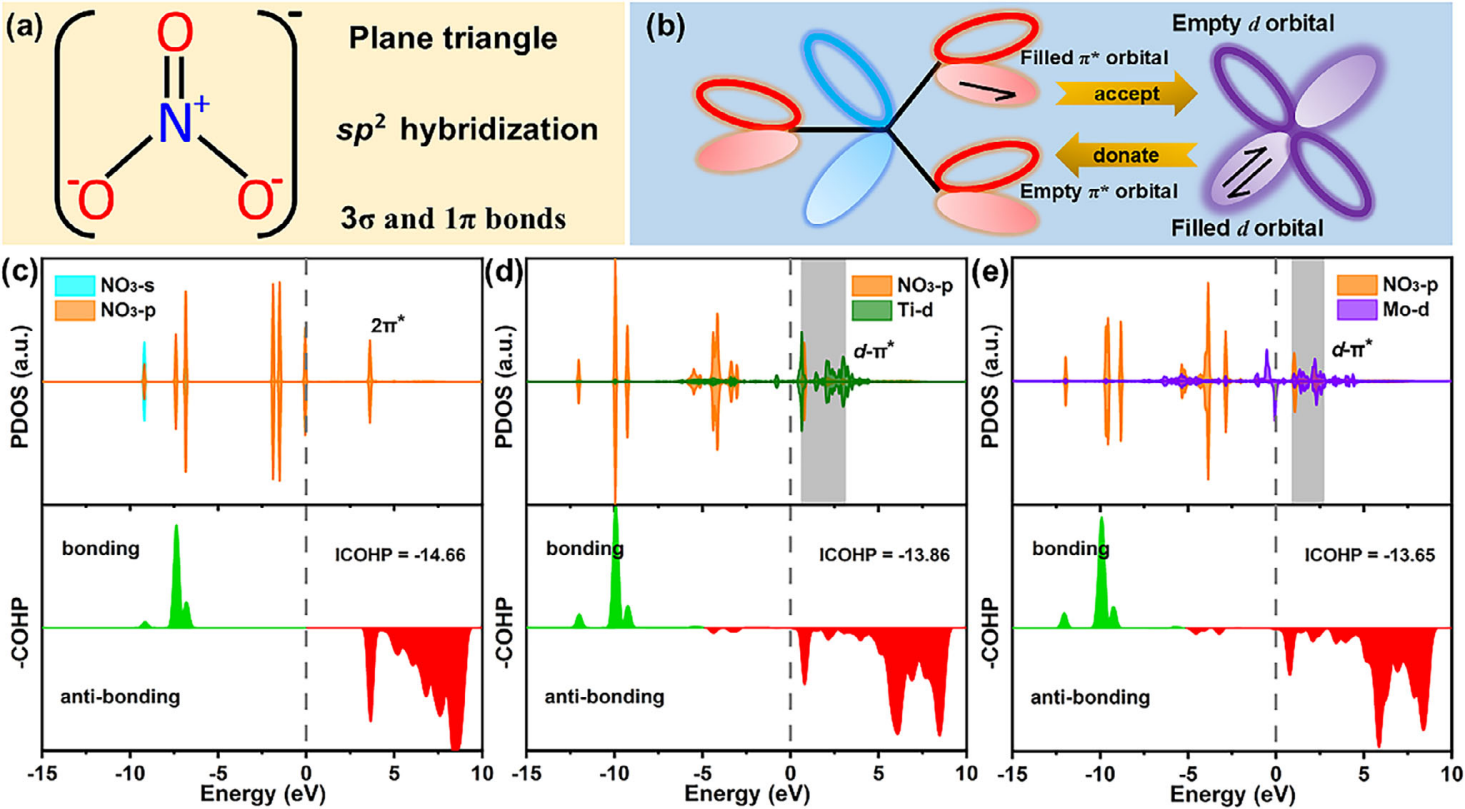
a) Illustration of the geometric configuration of NO_3_^−^. b) Overview of the activation mechanism for NO_3_RR on SACs. PDOS and COHP profiles for c) isolated NO_3_^−^, NO_3_‐adsorbed d) Ti‐N_4_@BPN and e) Mo‐N_4_@BPN, respectively.

Further analyses on the charge transfer, electronic structure, and the crystal orbital Hamilton population (COHP) have been performed to reveal the underlying mechanism of NO_3_^−^ activation and reduction. As illustrated in **Figure** **5**a, NO_3_^−^ exhibits a planar triangle geometry, featuring three 120° bond angles. When NO_3_^−^ adsorbs on SACs, the empty *d*‐orbitals of the active sites can accept electrons from NO_3_^−^, strengthening the TM─O bonds. Simultaneously, the filled *d*‐orbitals of active sites donate electrons back into the anti‐bonding orbital of NO_3_^−^, thereby weakening the N─O bond. This process facilitates sufficient TM─O orbital hybridization through the “acceptance–backdonation” mechanism which is demonstrated in Figure 5b. As revealed by the charge density difference in Figure S30 (Supporting Information), the charges are transferred from the *d* orbitals of metal centers to NO_3_^−^ adsorbed above Ti‐N_4_@BPN and Mo‐N_4_@BPN, giving rise to a weaker N═O bond by “pushing” electrons into the antibonding orbitals of NO_3_^−^.^[^ ^15^, ^40^ ^]^ The charge accumulation on O atoms and depletion between O and N atoms result in N═O bond activation, as reflected by the elongation of N═O bond lengths. The relevant electronic structure and bonding/antibonding characteristics of isolated NO_3_^−^, NO_3_‐adsorbed Ti‐N_4_@BPN and NO_3_‐adsorbed Mo‐N_4_@BPN are further calculated, as shown in Figure 5c–e. For isolated NO_3_^−^, there are several bonding and antibonding states. Once NO_3_^−^ adsorbs on active sites, the TM‐*d* orbitals overlap with NO_3_‐*p* orbitals to form hybridized orbitals, resulting in the formation of bonding and antibonding states in the TM‐NO_3_ complex. Specifically, both the TM‐*d* orbitals and NO_3_‐*p* orbitals split into the bonding and antibonding states, and the adsorption strength is determined by the antibonding states. Comparison of the DOS of NO_3_^−^ and TM before and after adsorption show that the *d*‐*p* hybrid interaction between metal and O leads to the redistribution of electrons, and the *d*‐band center moves up. Hybridization of the TM‐3*d* orbitals and 2π* orbitals of NO_3_^−^ can be observed, giving rise to the formation of *d*‐π* orbitals. The COHP can provide powerful clues into the activation of NO_3_^−^. As shown in Figure 5d,e, the activation of NO_3_^−^ is thus confirmed because a few antibonding states below *E*_F_ appear after NO_3_^−^ adsorbed on Ti‐N_4_@BPN and Mo‐N_4_@BPN.

During the NO_3_RR process, the intermediates can be divided into three moieties: absorbed intermediates (moiety 1), catalytic site (moiety 2) and BPN substrate (moiety 3), as illustrated in Figure S31a (Supporting Information). The charge variations of three moieties during the process of NO_3_RR on Ti‐N_4_@BPN and Mo‐N_4_@BPN are analyzed, as shown in Figure S31b (Supporting Information). It can be clearly seen that the trends of charge variation are similar for Ti‐N_4_@BPN and Mo‐N_4_@BPN. The Ti and Mo atoms keep positively charged in the whole reaction process with similar trends, which is significant to enhance the adsorption of intermediates and prevent the H poisoning. It should be noted that all calculations related to reaction energies of NO_3_RR are based on the revised Perdew‐Burke‐Ernzerhof (RPBE) functional, which has higher accuracy in modelling molecular adsorption.^[^ ^41^ ^]^ To further verify the reliability and generalization of these calculations, we compared the reaction trends of Ti‐N_4_@BPN and Mo‐N_4_@BPN by using RPBE and more commonly used PBE functionals. As shown in Figure S32 (Supporting Information), the results show that the reactions trends, PDS and overpotential, are almost similar under two functionals.

### 2.6 Universal Descriptor for NO_3_RR Performance

Clearly, a large‐scale screening of SACs would consume a huge computational burden due to the complicated reaction pathway of NO_3_RR. To achieve a rational design of optimal electrocatalysts toward NO_3_RR, it is important to identify a universal descriptor.^[^ ^11^, ^37^, ^42^ ^]^ Therefore, it is urgent to find the simple and generalized descriptors to predict the activity trends of NO_3_RR. Adsorbate scaling relations of intermediates can be used to model the relationships of key steps and the limiting potentials.^[^ ^42^, ^43^, ^44^, ^45^ ^]^ For example, the binding energy of N atoms is found to be a good indicator for NRR catalysts, and those catalysts with Δ*E*_N*_ being close to that of Ru (0001) are often excellent NRR catalysis.^[^ ^44^ ^]^

**Figure 6:**
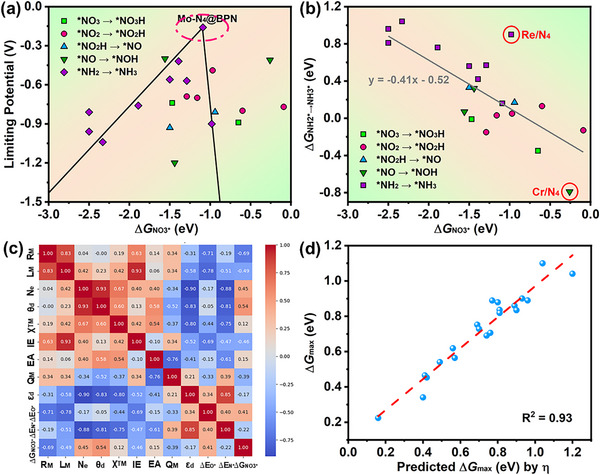
a) NO_3_RR activity plot of TM‐C*_x_* N*_y_* @BPN with a descriptor of Δ*G*_NO3*_. b) Relationship between the Δ*G* of *NH_2_ → *NH_3_ and Δ*G*_NO3*_. c) Pearson correlation heatmap between feature pairs. d) Fitting result between the descriptor and Δ*G*_max_.

Previous studies revealed that the adsorption strengths of O and N atoms (Δ*E*_O*_/Δ*E*_N*_) can act as descriptors for overall activity and selectivity of NO_3_RR electrocatalysts.^[^ ^11^ ^]^ Thus, the relationship between Δ*E*_O*_ and Δ*G*_NO3*_ is explored because NO_3_^−^ anchors on the active sites by O atoms. As shown in Figure S33 (Supporting Information), Δ*G*_NO3*_ is poorly correlated to Δ*E*_O*_ with R^2^ down to 0.17, which is different from the case on metal surfaces.^[^ ^11^ ^]^ In addition, taking Δ*G*_NO3*_ as a descriptor, the relationship between *U*_L_ and Δ*G*_NO3*_ is investigated, as shown in **Figure** **6**a. A volcano plot of *U*_L_ is established on those TM‐C*_x_* N*_y_* @BPN whose PDS is *NH_2_ → * NH_3_. This can be explained by the existing linear relationship between the Δ*G* from *NH_2_ to * NH_3_ and Δ*G*_NO3*_, as shown in Figure 6b. Yet, there are no obvious linear relationships between the free energy changes of other elementary steps and Δ*G*_NO3*_ (see Figure S34a–h, Supporting Information), largely due to the complexity of atomic species and varying coordination environments. Given the complexity of the catalyst configuration, it seems unlikely that a single descriptor would suffice for predicting the NO_3_^−^ adsorption and the overall NO_3_RR activity, necessitating the exploration of additional options.

The aforementioned observations suggest that the prediction of the NO_3_RR activity necessitates more intricate features beyond mere isolated descriptors. To identify the mathematical expressions that accurately describe the inherent features of these catalysts and their corresponding Δ*G*_max_, a symbolic regression fitting approach is developed in this work. Symbolic regression is an advanced machine learning technique, which can find optimal mathematical expressions to model a given dataset.^[^ ^46^, ^47^, ^48^ ^]^ As a supervised and explainable machine learning method, symbolic regression models require high‐quality feature sets. Considering the geometric and electronic properties of active sites and their coordination environments, the target variable Δ*G*_max_ could be determined by iteratively solving genetic operations. Therefore, twelve features were chosen as the input features, which can be divided into three categories: the elemental characteristics of active sites and local environments, the structural and electronic features of active sites and local environments, as well as adsorption energy descriptors. The detailed description of each features can be found in Table S20 (Supporting Information). The redox activity exhibits a strong correlation with the *d*‐orbital occupancy, such that the *d*‐electron count of the central metal (*θ*_d_) serves as a quantitative measure of this phenomenon. It is noteworthy that the *d*‐orbital occupancy is susceptible to influences such as charge transfer, and hence the electronegativity of the TM's coordinating atoms. Here we assume that a better descriptor needs a modification of Δ*G*_NO3*_, and other weakly correlated intermediates are not used in this fitting process. Based on the above twelve characteristics, the Pearson correlation coefficient is explored to reveal the correlations between the individual features. As shown in Figure 6c, the larger the absolute value of the correlation coefficient, the stronger the dependence between the two quantities. To avoid the redundant information, one of the variables with absolute values of correlation coefficients greater than 0.8 should be removed. Therefore, the variables of *L*_M_, *N*_e_, *θ*_d,_ and *ε*_d_ and are not considered in the fitting process. Subsequently, the remaining 8 features were used for symbolic regression fitting. Table S21 (Supporting Information) presents the mathematical operators used in this work. The resulting descriptor η can be expressed as

(2) $$\eta ={\left(Q_{M}^{0.5}-\Delta E_{N*}\times \Delta G_{\mathrm{NO}3*}-0.261\right)}^{0.25}$$

Here, *Q*_M_ represents the charge transfer from TM to substrate, and Δ*E*_N*_ is the adsorption strengths of N atoms. As shown in Figure 6d, the Δ*G*_max_ of twenty‐one TM‐C_x_N_y_@BPN systems based on this identified formula are highly consistent with the DFT calculated results. By four‐fold cross‐validation, the root mean squared errors (RMSE) of symbolic regression are 0.035, 0.072, 0.085, and 0.059 eV (see Figure S35, Supporting Information), implying the robustness of learned model. From this formula, it can be concluded that Δ*G*_NO3*_ requires a correction of *Q*_M_ and Δ*E*_N*_ to better describe Δ*G*_max_ of our studied systems. To further verify the rationality of feature selection and explore the multicollinearity among features, the recursive feature elimination (RFE) based on the least absolute shrinkage and selection operator (LASSO) estimator was used to assist this process. When the number of selected features is set to 4, *Q*_M_, *ε*_d_, Δ*E*_N*_ and Δ*G*_NO3*_ are regarded as the important features by RFE, which supports the learned results from symbolic regression.

### 2.7 Comparison with other Works and Experimental Prospect

In comparison to other related works, recent theoretical studies about the NO_3_RR activities based on SACs are discussed in this section. The Δ*G*_NO3*_, *U*_L_, PDS of these SACs are provided in Table S22 (Supporting Information). We can observe that the limiting potentials of the designed Ti‐N_4_@BPN and Mo‐N_4_@BPN are comparable to, even better than those of Fe‐N_4_/C (−0.30 V),^[^ ^13^ ^]^ Os‐N_4_/C (−0.42 V),^[^ ^27^ ^]^ Os/GDY (−0.37 V),^[^ ^49^ ^]^ Cr/GY (−0.23 V),^[^ ^47^ ^]^ Nb/PP (−0.24 V),^[^ ^50^ ^]^ Ti/g‐CN (−0.39 V),^[^ ^25^ ^]^ Ti/g‐C_3_N_4_ (−0.30 V),^[^ ^51^ ^]^ Hf/g‐C_2_N (−0.27 V),^[^ ^52^ ^]^ V/*h*‐BP (−0.22 V),^[^ ^53^ ^]^ V/GaN (−0.39 V),^[^ ^54^ ^]^ and Al_L_/Co_3_O_4_ (−0.25 V).^[^ ^55^ ^]^ In general, carbon atoms in graphene are intrinsically inert due to the strong π‐interactions of their hexagonal networks. Thus, their catalytic activity can be achieved by introducing foreign dopants or topological defects. 2D BPN can be regarded as graphene with many topological defects, thereby the C atoms in BPN may serve as active sites with inherent activity for certain chemical reactions, as demonstrated below. Furthermore, the electronic thermal conductivity (*k*_e_) of BPN is much higher than that of graphene, indicating that BPN has higher electrical conductivity (*σ*) which is beneficial for chemical reactions (*k*_e_ = *LσT*).^[^ ^56^ ^]^

In the seminal work, Fan et al. synthesized ultraflat BPN on Au(111) surface by the bottom‐up growth.^[^ ^31^ ^]^ Motivated by this work, many researchers explored its chemical activity and catalytic potential.^[^ ^34^, ^35^, ^36^, ^57^, ^58^, ^59^ ^]^ The Δ*G*_H*_ values of C_1_ site on free‐standing BPN and BPN/Al(111) are 0.13 and 0.11 eV, indicating their intrinsically excellent HER activity.^[^ ^57^ ^]^ In addition, the carbon atoms of tetragonal rings on 2D BPN are positively charged, thus resulting in good oxygen reduction activity due to the enhanced binding strength with oxygen intermediates.^[^ ^58^ ^]^ Experimentally, Kalapos et al. reported that BPN structure whose rings with different local (anti)aromaticity could act as the substrate on the synthesis of diarylethene‐based molecular switches.^[^ ^59^ ^]^ Based on above studies, we believe that this work will build the foundation for researchers to have a comprehensive understanding of BPN for NO_3_RR.

## 3 Conclusion

A high‐throughput in silico calculations with thousands of structures, including 15 intermediates of NO_3_RR, on 175 TM‐C*_x_* N*_y_* @BPN were performed in the present work. Taking the possible O‐end, O‐side, N‐end, N‐side, and NO‐dimer pathway into account, a full picture (stability, selectivity, activity, electronic origins and design strategies) of SACs supported on single‐layer BPN as NO_3_RR electrocatalysts is built up. We found that Mo‐N_4_@BPN exhibited great NO_3_RR performance with an outstanding limiting potentials of −0.16 V, exceeding the activity of benchmark Cu (100) surface. These SACs exhibit preferable NO_3_^−^ adsorption and strong orbital hybridization, with electronic backdonation from the metal *d* orbitals to π* orbitals of NO_3_^−^ to form the unoccupied *d*‐π* orbitals. It is anticipated that alongside the high‐throughput calculations with machine‐learning routes, our study sheds light on a new design principle for developing new SACs supported on carbon materials with excellent performance toward NO_3_RR.

## Conflict of Interest

The authors declare no conflict of interest.

## Author Contributions

Z.S. conceived the idea, conducted the simulations, and wrote the original draft; Z.S., H.J., H.X., Z.X., and Z.L. contributed to the computing resources, and reviewed the manuscript; M.‐F.N. and T.L.T. performed analysis and revised the manuscript; F.H. and Y.C. performed supervision, funding acquisition, and edited the manuscript.

## Supporting information



Supporting Information

## Data Availability

The data that support the findings of this study are available from the corresponding author upon reasonable request.
