# Supplementary material for: Design of Single‐Atom Catalysts Anchored in N‐Doped Biphenylene Using Symbolic Regression for Electrocatalytic Nitrate Reduction to Ammonia
Source: Adv Sci (Weinh). 2026 Jan 8;13(11):e12651. doi: 10.1002/advs.202512651 (PMC12931251; doi:10.1002/advs.202512651)
Supplement: Supplementary file 1 — Supporting Information [file ADVS-13-e12651-s001.docx]

**Supplementary Information**

**Design of Single-Atom Catalysts Anchored in N-Doped Biphenylene** **Using Symbolic Regression for Electrocatalytic Nitrate Reduction to Ammonia**

Zheng Shu^1,2^, Zhangsheng Shi^3^, Huaxian Jia^4^, Huifang Xu^1^, Zian Xu^1,5^, Zhongheng Li^1^, Man-Fai Ng^6^, Teck Leong Tan^6^, Fuqiang Huang^2,*^, Yongqing Cai^1,*^

^1^Joint Key Laboratory of the Ministry of Education, Institute of Applied Physics and Materials Engineering, University of Macau, Macau SAR 999078, China

^2^State Key Laboratory of Metal Matrix Composites, School of Materials Science and Engineering, Shanghai Jiao Tong University, Shanghai 200240, China

^3^Department of Chemistry, City University of Hong Kong, Hong Kong SAR 999077, China

^4^Tencent, AI for Life Sciences Lab, Shenzhen 518057, China

^5^Department of Materials Science and Engineering, Southern University of Science and Technology, Shenzhen 518055, China

^6^Institute of High Performance Computing (IHPC), Agency for Science, Technology and Research (A∗STAR), 1 Fusionopolis Way, #16-16 Connexis, 138632, Singapore

Correspondence and requests for materials should be addressed to Y. Cai. (E-mail: [yongqingcai@um.edu.mo](mailto:yongqingcai@um.edu.mo))

**1. DFT computational details**

All *in silico* first-principles calculations were implemented in the Vienna *ab initio* simulation package (VASP) with the projector-augmented wave pseudopotential (PAW) based on the spin-polarized density functional theory (DFT) methods.^[1-4]^ Instead of the Perdew-Burke-Ernzerhof (PBE) exchange-correlation functional, the revised PBE (RPBE) functional using the generalized gradient approximation (GGA) was performed to depict the exchange-correlation interaction, which has higher accuracy in modeling molecular adsorption on catalysts’ surfaces.^[5,6]^ The cut-offs of plane-wave basis set and the broadening width of Gaussian smearing were set to 450 eV and 0.05 eV, respectively. The DFT-D3 scheme of dispersion correction with the Becke-Jonson damping function was adopted to describe the van der Waals (vdW) interactions.^[7]^ The Brillouin-zone integration was sampled with a single Γ-point for the structural optimization, while a *k*-point grid of 2 × 2 × 1 was set for calculating total energies. The cell shape and atomic coordinates were allowed to fully relax in a fixed volume box until the Hellmann-Feynman forces acting on each atom converged to 0.02 eV Å^-1^. The single-layer BPN nanosheet including 96 carbon atoms was established as the substrate within a 4 × 3 × 1 supercell (*a* = 15.04 Å, *b* = 13.56 Å). The Cu (100) was modeled as a 4-layered (4 × 4) slab to benchmark the NO_3_RR performance. To avoid spurious interaction between the adjacent periodical images, the BPN sheets were separated by a vacuum of 20 Å along the *Z*-direction. The robust mixture of the Davidson and RMM-DIIS algorithms was employed to run iterative steps for these complex structures and all structures can reach the required accuracy. In addition, the energy of TM single atom was optimized in an asymmetric box (19 Å × 20 Å × 21 Å) for a better description of their degeneracy states using the single gamma point.^[6]^ The Bader charge analysis was conducted to investigate the charge transfer and spin states of active sites.^[8]^ The solvation effect was not considered since the solvation-induced overpotential changes can be negligible.^[9-11]^ To verify the thermal stability of these catalysts, the *ab initio* molecular dynamics (AIMD) simulations were conducted with an NVT ensemble of 10 ps.^[12]^ With the help of LOBSTER 3.1.0 packages,^[13]^ the bonding/antibonding population between N and O atoms can be obtained based on the projected crystal orbital Hamiltonian population (pCOHP).

**2. Stability calculations of the SACs**

One evaluation of catalyst thermodynamic stability is the difference between the binding energies of the metal atoms (*E*_b_) on substrates and their bulk cohesive energy (*E*_coh_), which can be calculated by:

$E_{b}=(E_{TM+sub}-{nE}_{\mathrm{TM}}-E_{\mathrm{sub}})/n$ (S1)

$E_{\mathrm{coh}}=(E_{TM-bulk}-{mE}_{\mathrm{TM}})/m$ (S2)

where *E*_TM+sub_, *E*_TM_, and *E*_sub_ denote the energies of TM-doped substrates, isolated TM single atom, and the substrates, respectively. *E*_TM-bulk_ is the total energy of metal bulk. *n* and *m* are the numbers of TM in doped systems and bulk phase. The formation energy (*E*_form_) of SACs equals to *E*_b_ − *E*_coh_. If *E*_form_ < 0, it is expected that SAC embedded into the substrates is more favorable than either the metal aggregation or the metal being leached. In addition, the dissolution potential (*U*_diss_) is another reference to confirm the electrochemical stability of the catalyst, and the calculation formula is given as follows:

$U_{\mathrm{diss}}=U_{\mathrm{diss}}^{^{\circ}}(metal,bulk)-\frac{E_{\mathrm{form}}}{ne}$ (S3)

where *U*^°^_diss_ (metal, bulk) and *E*_form_ are the standard metal dissolution potential and the formation energy given above, respectively. *n* represents the number of electrons involved in the dissolution process. If *U*_diss_ > 0, it is expected that SAC embedded into the substrates retains electrochemical stability under operating conditions.

**3. Gibbs free energy of NO_3_^¯^ adsorption**

The Gibbs free energy change of NO_3_^¯^ adsorption on the catalyst surface in aqueous solution (Δ*G*_NO3*_) can be calculated by the thermodynamic cycle.^[14]^ To avoid directly calculating the electronic energy of charged aqueous NO_3(l)_^¯^, the Gibbs free energy of gaseous HNO_3(g)_ can be used as the reference. The Gibbs free energy change of aqueous NO_3(l)_^¯^ adsorption can be described as:

${\Delta G}_{\mathrm{NO}_{3}*}=G_{\mathrm{NO}_{3}*}+\frac{1}{2}G_{H_{2}(g)}-G_{*}-G_{\mathrm{HNO}_{3}\left( g \right)}+{\Delta G}_{\mathrm{correct}}$ (S4)

where *G*_NO3*_, *G*_H2(g)_, *G*_*_ , *G*_HNO3(g)_ and ∆*G*_correct_ are the Gibbs free energy of NO_3_^¯^ adsorbed on TM-C_x_N_y_@BPN, H_2_ in the gas phase, TM-C_x_N_y_@BPN substrate, HNO_3_ in the gas phase, and the free energy correction of adsorption process, respectively. In this process, three steps should be considered: the formation of aqueous HNO_3(l)_, the vaporization of aqueous HNO_3(l)_ and the adsorption of gaseous HNO_3(g)_, as shown in Figure S13. According to the process of the thermodynamic cycle, ∆*G*_correct_ = ∆*G*_1_ + ∆*G*_2_. The change of Gibbs free energy of the vaporization of aqueous HNO_3(l)_ is 0.075 eV, while that of the formation of aqueous HNO_3(l)_ in solution is 0.317 eV. These Gibbs free energies can be obtained from the CRC handbook of chemistry and physics.^[15]^ Due to ∆*G*_1_ = 0.075 eV and ∆*G*_2_ = 0.317 eV, therefore ∆*G*_correct_ = 0.075 eV + 0.317 eV = 0.392 eV, which is consistent with previous studies.^[9-11,16-21]^

**4. Elementary steps in HER and NO_3_RR**

For the proton-coupled electron transfer (PCET) reactions, the reaction Gibbs free energies can be calculated according to the computational hydrogen electrode model developed by Nørskov and his co-workers,^[22,23]^ which can be expressed using the following equation:

$\Delta G=\Delta E+\Delta ZPE+-T\Delta S$ (S5)

where Δ*E* represents the energy difference between reactants and products. Δ*ZPE* and *T*Δ*S* are the difference between reactants and products in zero-point energy and entropic contribution at the temperature *T*.

The HER performance can be evaluated by computing the reaction free energy (Δ*G*_H*_) for hydrogen adsorption based on the proposed CHE model at 0 V versus RHE, that is, the equilibrium potential of the HER. The reaction Gibbs free energies of atomic H adsorption can be calculated using the following expression:

$\Delta G_{H^{*}}=\Delta E_{H^{*}}+\Delta E_{\mathrm{ZPE}}-T\Delta S_{H^{*}}$ (S6)

where Δ*E*_H*_, Δ*E*_ZPE_ and *T*Δ*S*_H*_ are the adsorption energy of atomic H, the correction of zero-point energy and the entropic contribution at the temperature *T*, respectively.

For NO_3_RR, the hydrogenation processes considered in this work for the search of minimum energy reaction pathways are presented here:

For N-end pathway:

*NO_3_ + H^+^ + *e*^-^ → *NO_3_H (R1)

*NO_3_H + H^+^ + *e*^-^ → *NO_2_ + H_2_O (R2)

*NO_2_ + H^+^ + *e*^-^ → *NO_2_H (R3)

*NO_2_H + H^+^ + *e*^-^ → *NO + H_2_O (R4)

*NO + H^+^ + *e*^-^ → *NOH (R5)

*NOH + H^+^ + *e*^-^ → *N + H_2_O (R6)

*N + H^+^ + *e*^-^ → *NH (R7)

*NH + H^+^ + *e*^-^ → *NH_2_ (R8)

*NH_2_ + H^+^ + *e*^-^ → *NH_3_ (R9)

*NH_3_ → * + NH_3_ (R10)

For N-side pathway, there are several different elementary reactions:

*NO_2_H + H^+^ + *e*^-^ → *N-*O + H_2_O (R11)

*N-*O + H^+^ + *e*^-^ → *NH-*O (R12)

*NH-*O + H^+^ + *e*^-^ → *NH_2_-*O (R13)

*NH_2_-*O + H^+^ + *e*^-^ → *O + NH_3_ (R14)

*O + H^+^ + *e*^-^ → *OH (R15)

*OH + H^+^ + *e*^-^ → *OH_2_ (R16)

*OH_2_ → * + H_2_O (R17)

For NO-dimer pathway:

*NO + *NO → *NO-*NO (R18)

*NO-*NO + H^+^ + *e*^-^ → *NO-*NOH (R19)

*NO-*NOH + H^+^ + *e*^-^ → *NO-*N + H_2_O (R20)

*NO-*N + H^+^ + *e*^-^ → *NOH-*N (R21)

*NOH-*N + H^+^ + *e*^-^ → *N-*N + H_2_O (R22)

*N-*N → * + N_2_ (R23)

**5. Potential-dependent selectivity for NO_3_RR vs HER**

The applied potential can aggravate the selectivity problem of NO_3_RR, because its role for Δ*G*_H*_ and Δ*G*_NO3*_ is different. Remarkably, the Δ*G*_NO3*_ becomes less favorable when the electrode potential becomes more negative, while the value of Δ*G*_H*_ presents the opposite situation.^[9,14]^ This is because one electron transfers from nitrate ions to the substrate, when **NO_3_⁻ adsorbs on** the substrate. **Therefore, the adsorption strength of NO_3_⁻ needs to be strong enough to overcome the** selectivity problem **of applied potential.**

**6. Implementation and fitting process of symbolic regression**

The symbolic regression based on the genetic algorithm was performed using scikit-learn and gplearn packages.^[21,24]^ The gplearn code extends the scikit-learn package to implement genetic algorithm with symbolic regression. It seeks to find a mathematical formula to represent a relationship between known feature space (*Φ*_0_) and their dependent targeting variable for a given dataset, beginning by building a set of naive random formulas. In each of these formulas, we have a mix of variables, constants and functional operators. The functional operators can be addition, subtraction, multiplication, even trigonometric function. As listed in Table S20, a set of algebraic and functional operations were iteratively applied to the initial *Φ*_0_ to construct augmented feature spaces with increased dimensionalities. Subsequently, the difference between target and predicted variables should be maximized or minimized using certain metric (such as mean squared error, root mean squared error and so on) in order to select the fittest individuals in this set of random formulas. By iteratively successive generation of this process, it can evolve their expression towards the truth. Finally, the evolution process will stop when it reaches the maximum number of generations or convergence threshold.

**Table S1.** The computed lattice constants, the binding energies of single metal atoms (*E*_b_), the cohesive energies of metal bulk (*E*_coh_), the formation energies of single metal atoms (*E*_form_), the dissolution potentials of SACs (*U*_diss_), the charge transfers (*δ*_TM_) and the magnetic moments (*M*_TM_) of single metal atoms on TM-C_4_@BPN coordination environments.

|  | *a* (Å) | *b* (Å) | *E*_b_ (eV) | *E*_coh_ (eV) | *E*_form_ (eV) | *U*^°^_diss_ (V) | *N*_e_ | *U*_diss_ (V) | *δ*_TM_ (*e*) | *M*_TM_ (μB) |
| --- | --- | --- | --- | --- | --- | --- | --- | --- | --- | --- |
| Substrate | 18.19 | 15.08 |  |  |  |  |  |  |  |  |
| Ti | 18.22 | 15.07 | -3.83 | -5.41 | +1.58 | -1.63 | 2 | -2.42 | +1.27 | 1.46 |
| V | 18.19 | 15.09 | -3.61 | -6.06 | +2.45 | -1.18 | 2 | -2.41 | +1.02 | 2.88 |
| Cr | 18.19 | 15.09 | -2.27 | -4.36 | +2.09 | -0.91 | 2 | -1.96 | +0.87 | 4.04 |
| Mn | 18.21 | 15.07 | -1.74 | -3.72 | +1.98 | -1.19 | 2 | -2.18 | +0.74 | 4.48 |
| Fe | 18.19 | 15.09 | -1.21 | -5.02 | +3.81 | -0.45 | 2 | -2.36 | +0.77 | -0.06 |
| Co | 18.17 | 15.09 | -2.27 | -5.31 | +3.04 | -0.28 | 2 | -1.80 | +0.61 | -0.03 |
| Ni | 18.15 | 15.09 | -3.14 | -5.13 | +1.99 | -0.26 | 2 | -1.26 | +0.54 | 0.00 |
| Cu | 18.20 | 15.08 | -1.48 | -3.48 | +2.00 | +0.34 | 2 | -0.66 | +0.52 | 0.00 |
| Zn | 18.19 | 15.08 | -0.38 | -1.10 | +0.72 | -0.76 | 2 | -1.12 | +0.07 | 0.00 |
| Zr | 18.23 | 15.06 | -4.76 | -6.30 | +1.54 | -1.45 | 4 | -1.84 | +1.41 | 0.72 |
| Nb | 18.18 | 15.09 | -3.62 | -6.92 | +3.30 | -1.10 | 3 | -2.20 | +1.12 | 2.10 |
| Mo | 18.20 | 15.08 | -2.24 | -6.32 | +4.08 | -0.20 | 3 | -1.56 | +0.81 | 3.49 |
| Ru | 18.10 | 15.10 | -4.17 | -6.78 | +2.61 | +0.46 | 2 | -0.85 | +0.59 | 0.00 |
| Rh | 18.09 | 15.09 | -4.03 | -5.84 | +1.81 | +0.60 | 2 | -0.31 | +0.40 | 0.00 |
| Pd | 18.19 | 15.09 | -2.32 | -3.75 | +1.43 | +0.95 | 2 | +0.24 | +0.27 | 0.00 |
| Ag | 18.26 | 15.07 | -0.90 | -2.51 | +1.61 | +0.80 | 1 | -0.81 | +0.40 | 0.00 |
| Cd | 18.19 | 15.08 | -0.24 | -0.73 | +0.49 | -0.40 | 2 | -0.65 | +0.03 | 0.00 |
| Hf | 18.24 | 15.06 | -4.10 | -6.41 | +2.31 | -1.55 | 4 | -2.13 | +1.51 | 0.54 |
| Ta | 18.12 | 15.11 | -5.27 | -8.24 | +2.97 | -0.60 | 3 | -1.59 | +1.36 | 0.68 |
| W | 18.17 | 15.09 | -3.06 | -8.39 | +5.33 | +0.10 | 3 | -1.68 | +0.74 | 2.79 |
| Re | 18.05 | 15.08 | -4.76 | -7.82 | +3.06 | +0.30 | 3 | -0.72 | +1.09 | 1.57 |
| Os | 18.02 | 15.07 | -5.55 | -8.33 | +2.78 | +0.84 | 8 | +0.49 | +0.48 | 0.27 |
| Ir | 18.04 | 15.07 | -5.10 | -7.35 | +2.25 | +1.16 | 3 | +0.41 | +0.34 | 0.00 |
| Pt | 18.18 | 15.09 | -3.55 | -5.58 | +2.03 | +1.18 | 2 | +0.17 | +0.00 | 0.02 |
| Au | 18.18 | 15.08 | -1.96 | -3.04 | +1.08 | +1.50 | 3 | +1.14 | -0.07 | 0.04 |

**Table S2.** The computed lattice constants, the binding energies of single metal atoms (*E*_b_), the cohesive energies of metal bulk (*E*_coh_), the formation energies of single metal atoms (*E*_form_), the dissolution potentials of SACs (*U*_diss_), the charge transfers (*δ*_TM_) and the magnetic moments (*M*_TM_) of single metal atoms on TM-C_3_N_1_@BPN coordination environments.

|  | *a* (Å) | *b* (Å) | *E*_b_ (eV) | *E*_coh_ (eV) | *E*_form_ (eV) | *U*^°^_diss_ (V) | *N*_e_ | *U*_diss_ (V) | *δ*_TM_ (*e*) | *M*_TM_ (μB) |
| --- | --- | --- | --- | --- | --- | --- | --- | --- | --- | --- |
| Substrate | 18.20 | 15.06 |  |  |  |  |  |  |  |  |
| Ti | 18.15 | 15.09 | -3.78 | -5.41 | +1.63 | -1.63 | 2 | -2.45 | +1.29 | 1.39 |
| V | 18.11 | 15.06 | -4.25 | -6.06 | +1.81 | -1.18 | 2 | -2.09 | +1.05 | 2.65 |
| Cr | 18.13 | 15.09 | -2.76 | -4.36 | +1.60 | -0.91 | 2 | -1.71 | +0.84 | 3.98 |
| Mn | 18.15 | 15.08 | -2.32 | -3.72 | +1.40 | -1.19 | 2 | -1.89 | +0.89 | 4.27 |
| Fe | 18.14 | 15.09 | -1.82 | -5.02 | +3.20 | -0.45 | 2 | -2.05 | +0.64 | 0.23 |
| Co | 18.12 | 15.08 | -2.85 | -5.31 | +2.46 | -0.28 | 2 | -1.51 | +0.63 | 0.00 |
| Ni | 18.20 | 15.06 | -3.13 | -5.13 | +2.00 | -0.26 | 2 | -1.26 | +0.59 | 0.06 |
| Cu | 18.21 | 15.05 | -1.47 | -3.48 | +2.01 | +0.34 | 2 | -0.66 | +0.36 | 0.00 |
| Zn | 18.20 | 15.06 | -0.34 | -1.10 | +0.76 | -0.76 | 2 | -1.14 | +0.05 | 0.00 |
| Zr | 18.19 | 15.05 | -5.08 | -6.30 | +1.22 | -1.45 | 4 | -1.76 | +1.41 | 0.67 |
| Nb | 18.11 | 15.06 | -4.40 | -6.92 | +2.52 | -1.10 | 3 | -1.94 | +1.09 | 2.03 |
| Mo | 18.12 | 15.08 | -3.29 | -6.32 | +3.03 | -0.20 | 3 | -1.21 | +0.92 | 2.58 |
| Ru | 18.12 | 15.08 | -4.50 | -6.78 | +2.28 | +0.46 | 2 | -0.68 | +0.61 | 0.14 |
| Rh | 18.07 | 15.07 | -4.54 | -5.84 | +1.30 | +0.60 | 2 | -0.05 | +0.39 | 0.00 |
| Pd | 18.20 | 15.07 | -2.27 | -3.75 | +1.48 | +0.95 | 2 | +0.21 | +0.25 | 0.03 |
| Ag | 18.21 | 15.05 | -0.84 | -2.51 | +1.67 | +0.80 | 1 | -0.87 | +0.31 | 0.00 |
| Cd | 18.20 | 15.05 | -0.25 | -0.73 | +0.48 | -0.40 | 2 | -0.64 | +0.03 | 0.00 |
| Hf | 18.17 | 15.08 | -4.91 | -6.41 | +1.50 | -1.55 | 4 | -1.93 | +1.49 | 0.42 |
| Ta | 18.14 | 15.09 | -5.38 | -8.24 | +2.86 | -0.60 | 3 | -1.55 | +1.32 | 1.12 |
| W | 18.12 | 15.09 | -4.41 | -8.39 | +3.98 | +0.10 | 3 | -1.23 | +0.92 | 2.13 |
| Re | 18.07 | 15.08 | -4.40 | -7.82 | +3.42 | +0.30 | 3 | -0.84 | +1.05 | 2.31 |
| Os | 18.04 | 15.07 | -5.30 | -8.33 | +3.03 | +0.84 | 8 | +0.46 | +0.44 | 0.97 |
| Ir | 18.04 | 15.07 | -5.62 | -7.35 | +1.73 | +1.16 | 3 | +0.58 | +0.31 | 0.00 |
| Pt | 18.18 | 15.06 | -3.62 | -5.58 | +1.96 | +1.18 | 2 | +0.20 | -0.02 | 0.03 |
| Au | 18.18 | 15.06 | -1.98 | -3.04 | +1.06 | +1.50 | 3 | +1.15 | -0.08 | 0.01 |

**Table S3.** The computed lattice constants, the binding energies of single metal atoms (*E*_b_), the cohesive energies of metal bulk (*E*_coh_), the formation energies of single metal atoms (*E*_form_), the dissolution potentials of SACs (*U*_diss_), the charge transfers (*δ*_TM_) and the magnetic moments (*M*_TM_) of single metal atoms on TM-C_2_N_2_^a^@BPN coordination environments.

|  | *a* (Å) | *b* (Å) | *E*_b_ (eV) | *E*_coh_ (eV) | *E*_form_ (eV) | *U*^°^_diss_ (V) | *N*_e_ | *U*_diss_ (V) | *δ*_TM_ (*e*) | *M*_TM_ (μB) |
| --- | --- | --- | --- | --- | --- | --- | --- | --- | --- | --- |
| Substrate | 18.20 | 15.04 |  |  |  |  |  |  |  |  |
| Ti | 18.08 | 15.11 | -5.19 | -5.41 | +0.22 | -1.63 | 2 | -1.74 | +1.37 | 1.10 |
| V | 18.00 | 15.10 | -6.70 | -6.06 | -0.64 | -1.18 | 2 | -0.86 | +1.25 | 1.34 |
| Cr | 18.10 | 15.08 | -3.29 | -4.36 | +1.07 | -0.91 | 2 | -1.45 | +0.79 | -3.90 |
| Mn | 18.11 | 15.08 | -2.87 | -3.72 | +0.85 | -1.19 | 2 | -1.62 | +0.90 | 4.19 |
| Fe | 18.12 | 15.07 | -2.65 | -5.02 | +2.37 | -0.45 | 2 | -1.64 | +0.65 | 0.02 |
| Co | 18.08 | 15.06 | -3.58 | -5.31 | +1.72 | -0.28 | 2 | -1.14 | +0.63 | 0.97 |
| Ni | 18.06 | 15.08 | -4.37 | -5.13 | +0.76 | -0.26 | 2 | -0.64 | +0.47 | 0.00 |
| Cu | 18.12 | 15.06 | -2.39 | -3.48 | +1.09 | +0.34 | 2 | -0.21 | +0.39 | 0.00 |
| Zn | 18.18 | 15.03 | -0.30 | -1.10 | +0.80 | -0.76 | 2 | -1.16 | +0.04 | 0.00 |
| Zr | 18.09 | 15.11 | -5.97 | -6.30 | +0.33 | -1.45 | 4 | -1.53 | +1.45 | 0.69 |
| Nb | 18.03 | 15.10 | -7.56 | -6.92 | -0.64 | -1.10 | 3 | -0.89 | +1.29 | 0.92 |
| Mo | 17.99 | 15.10 | -6.11 | -6.32 | +0.21 | -0.20 | 3 | -0.27 | +1.09 | 1.23 |
| Ru | 18.10 | 15.07 | -4.83 | -6.78 | +1.95 | +0.46 | 2 | -0.52 | +0.62 | 0.57 |
| Rh | 18.13 | 15.06 | -4.03 | -5.84 | +1.81 | +0.60 | 2 | -0.31 | +0.39 | 0.01 |
| Pd | 18.07 | 15.08 | -3.51 | -3.75 | +0.24 | +0.95 | 2 | +0.83 | +0.26 | 0.00 |
| Ag | 18.20 | 15.04 | -0.42 | -2.51 | +2.09 | +0.80 | 1 | -1.29 | +0.06 | 0.32 |
| Cd | 18.19 | 15.03 | -0.24 | -0.73 | +0.49 | -0.40 | 2 | -0.65 | +0.04 | 0.00 |
| Hf | 18.10 | 15.11 | -5.85 | -6.41 | +0.56 | -1.55 | 4 | -1.69 | +1.58 | 0.42 |
| Ta | 18.02 | 15.10 | -8.56 | -8.24 | -0.32 | -0.60 | 3 | -0.49 | +1.36 | 0.62 |
| W | 17.99 | 15.09 | -7.43 | -8.39 | +0.96 | +0.10 | 3 | -0.22 | +0.98 | 1.24 |
| Re | 18.08 | 15.08 | -4.57 | -7.82 | +3.25 | +0.30 | 3 | -0.78 | +1.04 | 1.07 |
| Os | 18.07 | 15.08 | -5.37 | -8.33 | +2.96 | +0.84 | 8 | +0.47 | +0.40 | 0.73 |
| Ir | 18.08 | 15.07 | -5.23 | -7.35 | +2.12 | +1.16 | 3 | +0.45 | +0.32 | 0.23 |
| Pt | 18.04 | 15.06 | -5.51 | -5.58 | +0.07 | +1.18 | 2 | +1.15 | +0.11 | 0.00 |
| Au | 18.15 | 15.05 | -2.32 | -3.04 | +0.72 | +1.50 | 3 | +1.26 | -0.06 | 0.00 |

**Table S4.** The computed lattice constants, the binding energies of single metal atoms (*E*_b_), the cohesive energies of metal bulk (*E*_coh_), the formation energies of single metal atoms (*E*_form_), the dissolution potentials of SACs (*U*_diss_), the charge transfers (*δ*_TM_) and the magnetic moments (*M*_TM_) of single metal atoms on TM-C_2_N_2_^b^@BPN coordination environments.

|  | *a* (Å) | *b* (Å) | *E*_b_ (eV) | *E*_coh_ (eV) | *E*_form_ (eV) | *U*^°^_diss_ (V) | *N*_e_ | *U*_diss_ (V) | *δ*_TM_ (*e*) | *M*_TM_ (μB) |
| --- | --- | --- | --- | --- | --- | --- | --- | --- | --- | --- |
| Substrate | 18.20 | 15.04 |  |  |  |  |  |  |  |  |
| Ti | 17.93 | 15.05 | -10.68 | -5.41 | -5.27 | -1.63 | 2 | +1.01 | +1.76 | 0.04 |
| V | 18.06 | 15.06 | -4.90 | -6.06 | +1.16 | -1.18 | 2 | -1.76 | +1.00 | 2.66 |
| Cr | 18.07 | 15.07 | -3.48 | -4.36 | +0.88 | -0.91 | 2 | -1.35 | +0.83 | -3.89 |
| Mn | 18.06 | 15.10 | -3.11 | -3.72 | +0.61 | -1.19 | 2 | -1.49 | +0.99 | 4.12 |
| Fe | 18.06 | 15.09 | -3.33 | -5.02 | +1.69 | -0.45 | 2 | -1.30 | +0.76 | 2.99 |
| Co | 18.03 | 15.07 | -3.71 | -5.31 | +1.60 | -0.28 | 2 | -1.08 | +0.62 | 1.50 |
| Ni | 18.03 | 15.05 | -4.36 | -5.13 | +0.77 | -0.26 | 2 | -0.65 | +0.49 | 0.00 |
| Cu | 18.14 | 15.06 | -2.19 | -3.48 | +1.29 | +0.34 | 2 | -0.31 | +0.27 | 0.00 |
| Zn | 18.11 | 15.06 | -0.69 | -1.10 | +0.41 | -0.76 | 2 | -0.97 | +0.26 | 0.02 |
| Zr | 18.01 | 15.06 | -7.91 | -6.30 | -1.61 | -1.45 | 4 | -1.05 | +1.59 | 0.00 |
| Nb | 17.99 | 15.06 | -6.75 | -6.92 | +0.17 | -1.10 | 3 | -1.16 | +1.36 | 0.46 |
| Mo | 18.06 | 15.07 | -4.05 | -6.32 | +2.27 | -0.20 | 3 | -0.96 | +0.78 | 3.35 |
| Ru | 18.02 | 15.05 | -4.71 | -6.78 | +2.07 | +0.46 | 2 | -0.58 | +0.63 | 0.17 |
| Rh | 18.05 | 15.03 | -4.70 | -5.84 | +1.14 | +0.60 | 2 | +0.03 | +0.39 | 0.28 |
| Pd | 18.05 | 15.05 | -3.42 | -3.75 | +0.33 | +0.95 | 2 | +0.78 | +0.26 | 0.00 |
| Ag | 18.18 | 15.04 | -0.37 | -2.51 | +2.14 | +0.80 | 1 | -1.34 | +0.05 | 0.33 |
| Cd | 18.20 | 15.04 | -0.31 | -0.73 | +0.42 | -0.40 | 2 | -0.61 | +0.06 | 0.00 |
| Hf | 18.01 | 15.06 | -7.45 | -6.41 | -1.04 | -1.55 | 4 | -1.29 | +1.75 | 0.00 |
| Ta | 17.93 | 15.05 | -12.59 | -8.24 | -4.35 | -0.60 | 3 | +0.85 | +1.82 | 0.34 |
| W | 18.05 | 15.07 | -5.06 | -8.39 | +3.33 | +0.10 | 3 | -1.01 | +0.79 | 2.82 |
| Re | 18.05 | 15.08 | -4.25 | -7.82 | +3.57 | +0.30 | 3 | -0.89 | +1.00 | 2.99 |
| Os | 18.03 | 15.05 | -5.27 | -8.33 | +3.06 | +0.84 | 8 | +0.46 | +0.40 | 1.53 |
| Ir | 18.02 | 15.03 | -5.65 | -7.35 | +1.70 | +1.16 | 3 | +0.59 | +0.34 | 0.45 |
| Pt | 18.00 | 15.05 | -5.65 | -5.58 | -0.07 | +1.18 | 2 | +1.21 | +0.13 | 0.00 |
| Au | 18.19 | 15.07 | -2.62 | -3.04 | +0.42 | +1.50 | 3 | +1.36 | -0.01 | 0.00 |

**Table S5.** The computed lattice constants, the binding energies of single metal atoms (*E*_b_), the cohesive energies of metal bulk (*E*_coh_), the formation energies of single metal atoms (*E*_form_), the dissolution potentials of SACs (*U*_diss_), the charge transfers (*δ*_TM_) and the magnetic moments (*M*_TM_) of single metal atoms on TM-C_2_N_2_^c^@BPN coordination environments.

|  | *a* (Å) | *b* (Å) | *E*_b_ (eV) | *E*_coh_ (eV) | *E*_form_ (eV) | *U*^°^_diss_ (V) | *N*_e_ | *U*_diss_ (V) | *δ*_TM_ (*e*) | *M*_TM_ (μB) |
| --- | --- | --- | --- | --- | --- | --- | --- | --- | --- | --- |
| Substrate | 18.17 | 15.08 |  |  |  |  |  |  |  |  |
| Ti | 18.09 | 15.11 | -6.65 | -5.41 | -1.24 | -1.63 | 2 | -1.01 | +1.64 | 0.00 |
| V | 18.03 | 15.07 | -6.99 | -6.06 | -0.93 | -1.18 | 2 | -0.72 | +1.32 | 2.12 |
| Cr | 18.05 | 15.07 | -4.96 | -4.36 | -0.60 | -0.91 | 2 | -0.61 | +1.19 | 3.24 |
| Mn | 18.14 | 15.09 | -1.95 | -3.72 | +1.77 | -1.19 | 2 | -2.08 | +0.78 | 4.53 |
| Fe | 18.07 | 15.07 | -4.85 | -5.02 | +0.17 | -0.45 | 2 | -0.54 | +0.95 | 2.10 |
| Co | 18.01 | 15.07 | -4.72 | -5.31 | +0.59 | -0.28 | 2 | -0.58 | +0.87 | 1.22 |
| Ni | 18.05 | 15.06 | -5.06 | -5.13 | +0.07 | -0.26 | 2 | -0.30 | +0.79 | 0.00 |
| Cu | 18.14 | 15.08 | -1.71 | -3.48 | +1.77 | +0.34 | 2 | -0.54 | +0.58 | 0.00 |
| Zn | 18.12 | 15.09 | -0.39 | -1.10 | +0.71 | -0.76 | 2 | -1.12 | +0.08 | 0.00 |
| Zr | 18.08 | 15.10 | -8.40 | -6.30 | -2.10 | -1.45 | 4 | -0.92 | +1.78 | 0.00 |
| Nb | 18.02 | 15.08 | -7.29 | -6.92 | -0.37 | -1.10 | 3 | -0.98 | +1.42 | 1.29 |
| Mo | 17.92 | 15.05 | -9.77 | -6.32 | -3.45 | -0.20 | 3 | +0.95 | +1.42 | 0.99 |
| Ru | 17.96 | 15.07 | -6.61 | -6.78 | +0.17 | +0.46 | 2 | +0.38 | +0.99 | 0.00 |
| Rh | 18.01 | 15.06 | -5.23 | -5.84 | +0.61 | +0.60 | 2 | +0.30 | +0.73 | 0.31 |
| Pd | 18.13 | 15.10 | -2.35 | -3.75 | +1.40 | +0.95 | 2 | +0.25 | +0.28 | 0.00 |
| Ag | 18.15 | 15.08 | -1.00 | -2.51 | +1.51 | +0.80 | 1 | -0.71 | +0.45 | 0.00 |
| Cd | 18.16 | 15.08 | -0.28 | -0.73 | +0.45 | -0.40 | 2 | -0.63 | +0.04 | 0.00 |
| Hf | 18.06 | 15.10 | -8.34 | -6.41 | -1.93 | -1.55 | 4 | -1.07 | +1.95 | 0.00 |
| Ta | 17.94 | 15.05 | -12.00 | -8.24 | -3.10 | -0.60 | 3 | +0.43 | +1.76 | 0.58 |
| W | 17.94 | 15.04 | -11.72 | -8.39 | -3.76 | +0.10 | 3 | +1.35 | +1.56 | 0.96 |
| Re | 17.93 | 15.05 | -11.21 | -7.82 | -3.39 | +0.30 | 3 | +1.43 | +2.15 | 0.00 |
| Os | 17.94 | 15.06 | -7.49 | -8.33 | +0.84 | +0.84 | 8 | +0.74 | +0.88 | 0.00 |
| Ir | 17.99 | 15.06 | -6.25 | -7.35 | +1.10 | +1.16 | 3 | +0.79 | +0.80 | 0.41 |
| Pt | 18.11 | 15.10 | -3.64 | -5.58 | +1.94 | +1.18 | 2 | +0.21 | +0.00 | 0.00 |
| Au | 18.13 | 15.08 | -2.01 | -3.04 | +1.03 | +1.50 | 3 | +1.16 | -0.06 | 0.03 |

**Table S6.** The computed lattice constants, the binding energies of single metal atoms (*E*_b_), the cohesive energies of metal bulk (*E*_coh_), the formation energies of single metal atoms (*E*_form_), the dissolution potentials of SACs (*U*_diss_), the charge transfers (*δ*_TM_) and the magnetic moments (*M*_TM_) of single metal atoms on TM-C_1_N_3_@BPN coordination environments.

|  | *a* (Å) | *b* (Å) | *E*_b_ (eV) | *E*_coh_ (eV) | *E*_form_ (eV) | *U*^°^_diss_ (V) | *N*_e_ | *U*_diss_ (V) | *δ*_TM_ (*e*) | *M*_TM_ (μB) |
| --- | --- | --- | --- | --- | --- | --- | --- | --- | --- | --- |
| Substrate | 18.14 | 15.07 |  |  |  |  |  |  |  |  |
| Ti | 17.89 | 15.12 | -10.15 | -5.41 | -4.74 | -1.63 | 2 | +0.74 | +1.80 | 0.03 |
| V | 17.89 | 15.12 | -9.60 | -6.06 | -3.54 | -1.18 | 2 | +0.59 | +1.58 | 1.26 |
| Cr | 17.88 | 15.12 | -7.52 | -4.36 | -3.16 | -0.91 | 2 | +0.67 | +1.34 | 2.72 |
| Mn | 18.09 | 15.09 | -2.71 | -3.72 | +1.01 | -1.19 | 2 | -1.69 | +0.95 | 4.33 |
| Fe | 18.09 | 15.08 | -2.84 | -5.02 | +2.18 | -0.45 | 2 | -1.54 | +0.75 | 3.08 |
| Co | 18.09 | 15.07 | -2.38 | -5.31 | +2.93 | -0.28 | 2 | -1.75 | +0.61 | -1.89 |
| Ni | 18.07 | 15.06 | -3.44 | -5.13 | +1.69 | -0.26 | 2 | -1.11 | +0.52 | 0.00 |
| Cu | 18.08 | 15.08 | -2.29 | -3.48 | +1.19 | +0.34 | 2 | -0.26 | +0.34 | 0.00 |
| Zn | 18.10 | 15.08 | -0.58 | -1.10 | +0.52 | -0.76 | 2 | -1.02 | +0.20 | 0.04 |
| Zr | 17.89 | 15.11 | -11.07 | -6.30 | -4.77 | -1.45 | 4 | -0.26 | +1.96 | 0.00 |
| Nb | 17.91 | 15.01 | -11.06 | -6.92 | -4.14 | -1.10 | 3 | +0.28 | +1.64 | 0.91 |
| Mo | 17.87 | 15.13 | -8.92 | -6.32 | -2.60 | -0.20 | 3 | +0.67 | +1.44 | 0.99 |
| Ru | 17.95 | 15.06 | -6.92 | -6.78 | -0.14 | +0.46 | 2 | +0.53 | +1.00 | 0.01 |
| Rh | 18.07 | 15.07 | -3.63 | -5.84 | +2.21 | +0.60 | 2 | -0.51 | +0.38 | 0.38 |
| Pd | 18.13 | 15.06 | -2.29 | -3.75 | +1.46 | +0.95 | 2 | +0.22 | +0.30 | 0.03 |
| Ag | 18.10 | 15.07 | -1.53 | -2.51 | +0.98 | +0.80 | 1 | -0.18 | +0.30 | 0.00 |
| Cd | 18.12 | 15.05 | -0.20 | -0.73 | +0.53 | -0.40 | 2 | -0.67 | +0.03 | 0.00 |
| Hf | 17.88 | 15.11 | -11.02 | -6.41 | -4.61 | -1.55 | 4 | -0.40 | +2.16 | 0.00 |
| Ta | 18.01 | 15.09 | -8.82 | -8.24 | -0.58 | -0.60 | 3 | -0.41 | +1.61 | 0.85 |
| W | 17.89 | 15.02 | -11.84 | -8.39 | -3.45 | +0.10 | 3 | +1.25 | +1.62 | 1.13 |
| Re | 18.08 | 15.09 | -2.95 | -7.82 | +4.87 | +0.30 | 3 | -1.32 | +0.79 | 3.77 |
| Os | 17.95 | 15.07 | -7.58 | -8.33 | +0.75 | +0.84 | 8 | +0.75 | +0.84 | 0.07 |
| Ir | 17.96 | 15.04 | -6.69 | -7.35 | +0.66 | +1.16 | 3 | +0.94 | +0.83 | 0.16 |
| Pt | 18.13 | 15.06 | -3.49 | -5.58 | +2.09 | +1.18 | 2 | +0.14 | +0.05 | 0.07 |
| Au | 18.09 | 15.08 | -2.36 | -3.04 | +0.68 | +1.50 | 3 | +1.27 | -0.04 | 0.00 |

**Table S7.** The computed lattice constants, the binding energies of single metal atoms (*E*_b_), the cohesive energies of metal bulk (*E*_coh_), the formation energies of single metal atoms (*E*_form_), the dissolution potentials of SACs (*U*_diss_), the charge transfers (*δ*_TM_) and the magnetic moments (*M*_TM_) of single metal atoms on TM-N_4_@BPN coordination environments.

|  | *a* (Å) | *b* (Å) | *E*_b_ (eV) | *E*_coh_ (eV) | *E*_form_ (eV) | *U*^°^_diss_ (V) | *N*_e_ | *U*_diss_ (V) | *δ*_TM_ (*e*) | *M*_TM_ (μB) |
| --- | --- | --- | --- | --- | --- | --- | --- | --- | --- | --- |
| Substrate | 18.12 | 15.07 |  |  |  |  |  |  |  |  |
| Ti | 17.90 | 15.05 | -11.12 | -5.41 | -5.71 | -1.63 | 2 | +1.23 | +1.90 | 0.00 |
| V | 17.90 | 15.02 | -10.67 | -6.06 | -4.61 | -1.18 | 2 | +1.13 | +1.59 | 1.62 |
| Cr | 17.89 | 15.05 | -8.85 | -4.36 | -4.49 | -0.91 | 2 | +1.34 | +1.44 | 2.63 |
| Mn | 18.08 | 15.07 | -2.05 | -3.72 | +1.67 | -1.19 | 2 | -2.03 | +0.81 | 4.58 |
| Fe | 17.88 | 15.06 | -8.95 | -5.02 | -3.93 | -0.45 | 2 | +1.52 | +1.19 | 1.70 |
| Co | 17.86 | 15.08 | -9.13 | -5.31 | -3.82 | -0.28 | 2 | +1.63 | +1.05 | 0.59 |
| Ni | 17.86 | 15.09 | -8.97 | -5.13 | -3.84 | -0.26 | 2 | +1.66 | +1.07 | 0.00 |
| Cu | 18.07 | 15.08 | -2.27 | -3.48 | +1.21 | +0.34 | 2 | -0.26 | +0.62 | 0.00 |
| Zn | 18.08 | 15.07 | -0.23 | -1.10 | +0.87 | -0.76 | 2 | -1.20 | +0.03 | 0.00 |
| Zr | 17.91 | 15.04 | -11.97 | -6.30 | -5.67 | -1.45 | 4 | -0.03 | +2.07 | 0.00 |
| Nb | 17.89 | 15.03 | -11.06 | -6.92 | -4.14 | -1.10 | 3 | +0.28 | +1.75 | 0.97 |
| Mo | 17.89 | 15.03 | -9.93 | -6.32 | -3.61 | -0.20 | 3 | +1.00 | +1.59 | 1.61 |
| Ru | 17.88 | 15.02 | -9.94 | -6.78 | -3.16 | +0.46 | 2 | +2.04 | +1.34 | 0.00 |
| Rh | 17.88 | 15.03 | -8.65 | -5.84 | -2.81 | +0.60 | 2 | +2.01 | +1.10 | 0.07 |
| Pd | 18.07 | 15.07 | -0.48 | -3.75 | +3.27 | +0.95 | 2 | -0.69 | +0.21 | -0.10 |
| Ag | 18.08 | 15.07 | -1.13 | -2.51 | +1.38 | +0.80 | 1 | -0.58 | +0.49 | 0.00 |
| Cd | 18.12 | 15.07 | -0.25 | -0.73 | +0.48 | -0.40 | 2 | -0.64 | +0.04 | 0.00 |
| Hf | 17.91 | 15.02 | -11.03 | -6.41 | -4.62 | -1.55 | 4 | -0.40 | +2.13 | 0.16 |
| Ta | 17.90 | 15.03 | -11.80 | -8.24 | -3.56 | -0.60 | 3 | +0.59 | +1.93 | 0.64 |
| W | 17.89 | 15.04 | -10.60 | -8.39 | -2.21 | +0.10 | 3 | +0.84 | +1.66 | 1.34 |
| Re | 17.87 | 15.03 | -10.86 | -7.82 | -3.04 | +0.30 | 3 | +1.31 | +2.10 | 0.51 |
| Os | 17.87 | 15.03 | -11.26 | -8.33 | -2.93 | +0.84 | 8 | +1.21 | +1.24 | 0.00 |
| Ir | 17.88 | 15.04 | -10.36 | -7.35 | -3.01 | +1.16 | 3 | +2.16 | +1.30 | 0.00 |
| Pt | 17.88 | 15.05 | -8.82 | -5.58 | -3.24 | +1.18 | 2 | +2.80 | +1.07 | 0.00 |
| Au | 18.07 | 15.35 | -0.98 | -3.04 | +2.06 | +1.50 | 3 | +0.81 | +0.02 | 0.14 |

**Table S8.** Formation energies of TM single atoms embedded in six-atom ring (*E*_form-N4@C6_) and eight-atom ring (*E*_form-N4@C8_) with N_4_ coordination environments. The unit is eV.

| Elemental type | *E*_form-N4@C6_ | *E*_form-N4@C8_ |
| --- | --- | --- |
| Ti | -0.33 | -5.71 |
| V | +0.69 | -4.61 |
| Cr | +4.55 | -4.49 |
| Mn | +1.21 | +1.67 |
| Fe | +2.66 | -3.93 |
| Co | +2.44 | -3.82 |
| Ni | +1.76 | -3.84 |
| Cu | +1.07 | +1.21 |
| Zn | +0.81 | +0.87 |
| Zr | -0.16 | -5.67 |
| Nb | +1.31 | -4.14 |
| Mo | +2.27 | -3.61 |
| Ru | +1.30 | -3.16 |
| Rh | +0.84 | -2.81 |
| Pd | +1.03 | +3.27 |
| Ag | +0.69 | +1.38 |
| Cd | +0.44 | +0.48 |
| Hf | +1.44 | -4.62 |
| Ta | +3.29 | -3.56 |
| W | +4.75 | -2.21 |
| Re | +3.62 | -3.04 |
| Os | +6.18 | -2.93 |
| Ir | +1.65 | -3.01 |
| Pt | -0.02 | -3.24 |
| Au | +0.41 | +2.06 |

**Table S9.** The Gibbs free energy change of NO_3_^¯^ and H adsorption on TM-C_4_@BPN *via* 1-O and 2-O patterns, and the corresponding length of TM-O bonds.

| 1-O pattern 2-O pattern | | | | | | H adsorption | |
| --- | --- | --- | --- | --- | --- | --- | --- |
|  | $\Delta G_{*NO3}$(eV) | *d*_TM-O_ (Å) | $\Delta G_{*NO3}$(eV) | *d*_TM-O1_ (Å) | *d*_TM-O2_ (Å) | $\Delta G_{*H}$(eV) | *d*_TM-H_ (Å) |
| Ti | **/** | **/** | -1.91 | 2.06 | 2.11 | -0.74 | 1.75 |
| V | -0.47 | 1.83 | -1.55 | 2.05 | 2.05 | -0.44 | 1.69 |
| Cr | **/** | **/** | +0.46 | 2.05 | 2.05 | -0.24 | 1.67 |
| Mn | **/** | **/** | -1.03 | 2.11 | 2.11 | -0.76 | 1.64 |
| Fe | **/** | **/** | -0.90 | 2.05 | 2.07 | -1.51 | 1.59 |
| Co | **/** | **/** | -1.80 | 2.10 | 2.11 | -0.29 | 1.50 |
| Ni | **/** | **/** | -0.35 | 2.00 | 2.10 | -0.09 | 1.52 |
| Cu | **/** | **/** | -0.58 | 2.05 | 2.06 | -0.71 | 1.52 |
| Zn |  |  |  |  |  |  |  |
| Zr | **/** | **/** | -2.29 | 2.18 | 2.18 | -1.07 | 1.87 |
| Nb | **/** | **/** | -1.75 | 2.14 | 2.17 | -0.72 | 1.80 |
| Mo | **/** | **/** | -1.32 | 2.14 | 2.17 | -0.53 | 1.80 |
| Ru | **/** | **/** | -0.07 | 2.08 | 2.09 | -0.17 | 1.62 |
| Rh | **/** | **/** | -0.05 | 2.11 | 2.11 | -0.04 | 1.55 |
| Pd | **/** | **/** | -0.33 | 2.20 | 2.21 | -0.19 | 1.62 |
| Ag | **/** | **/** | +0.02 | 2.26 | 2.40 | -0.48 | 1.65 |
| Cd |  |  |  |  |  |  |  |
| Hf | **/** | **/** | -2.71 | 2.13 | 2.13 | -1.43 | 1.85 |
| Ta | **/** | **/** | -2.31 | 2.14 | 2.19 | -1.39 | 1.82 |
| W | **/** | **/** | -2.11 | 2.04 | 2.11 | -1.06 | 1.74 |
| Re | **/** | **/** | -1.38 | 2.12 | 2.13 | -1.10 | 1.70 |
| Os | **/** | **/** | -1.03 | 2.20 | 2.22 | -0.85 | 1.68 |
| Ir | **/** | **/** | -0.47 | 2.09 | 2.10 | -0.59 | 1.61 |
| Pt | **/** | **/** | -0.70 | 2.17 | 2.17 | -1.16 | 1.62 |
| Au | +0.67 | 2.06 | **/** | **/** | **/** | -0.59 | 1.60 |

**Table S10.** The Gibbs free energy change of NO_3_^¯^ and H adsorption on TM-C_3_N_1_@BPN *via* 1-O and 2-O patterns, and the corresponding length of TM-O bonds.

| 1-O pattern 2-O pattern | | | | | | H adsorption | |
| --- | --- | --- | --- | --- | --- | --- | --- |
|  | $\Delta G_{*NO3}$(eV) | *d*_TM-O_ (Å) | $\Delta G_{*NO3}$(eV) | *d*_TM-O1_ (Å) | *d*_TM-O2_ (Å) | $\Delta G_{*H}$(eV) | *d*_TM-H_ (Å) |
| Ti | **/** | **/** | -2.09 | 2.06 | 2.13 | -0.74 | 1.75 |
| V | **/** | **/** | -1.73 | 2.05 | 2.08 | -0.73 | 1.69 |
| Cr | **/** | **/** | -1.16 | 2.04 | 2.04 | -0.27 | 1.67 |
| Mn | **/** | **/** | +0.99 | 2.09 | 2.12 | -0.58 | 1.63 |
| Fe | **/** | **/** | -1.11 | 2.02 | 2.07 | -0.31 | 1.59 |
| Co | **/** | **/** | -1.97 | 2.00 | 2.02 | -0.42 | 1.55 |
| Ni | **/** | **/** | -0.94 | 2.01 | 2.01 | -0.50 | 1.53 |
| Cu | **/** | **/** | -0.53 | 2.05 | 2.06 | -0.74 | 1.51 |
| Zn |  |  |  |  |  |  |  |
| Zr | **/** | **/** | -2.34 | 2.18 | 2.19 | -1.08 | 1.87 |
| Nb | **/** | **/** | -2.37 | 2.18 | 2.21 | -1.48 | 1.81 |
| Mo | **/** | **/** | -2.20 | 2.16 | 2.16 | -0.67 | 1.75 |
| Ru | -0.32 | 1.95 | -0.86 | 2.09 | 2.10 | -0.67 | 1.61 |
| Rh | **/** | **/** | -0.37 | 2.11 | 2.12 | +0.04 | 1.59 |
| Pd | **/** | **/** | -0.29 | 2.21 | 2.22 | -0.22 | 1.63 |
| Ag | **/** | **/** | +0.11 | 2.29 | 2.30 | -0.35 | 1.65 |
| Cd |  |  |  |  |  |  |  |
| Hf | **/** | **/** | -3.81 | 2.17 | 2.20 | -1.33 | 1.85 |
| Ta | **/** | **/** | -6.10 | 1.76 | 1.89 | -1.37 | 1.80 |
| W | **/** | **/** | -2.94 | 2.14 | 2.14 | -1.07 | 1.75 |
| Re | **/** | **/** | -1.10 | 2.11 | 2.29 | -0.95 | 1.70 |
| Os | **/** | **/** | -0.93 | 2.11 | 2.20 | -1.17 | 1.60 |
| Ir | **/** | **/** | -0.42 | 2.06 | 2.19 | -0.69 | 1.59 |
| Pt | **/** | **/** | -0.65 | 2.17 | 2.18 | -1.11 | 1.62 |
| Au | **/** | **/** | +0.56 | 2.21 | 2.37 | -0.67 | 1.59 |

**Table S11.** The Gibbs free energy change of NO_3_^¯^ and H adsorption on TM-C_2_N_2_^a^@BPN *via* 1-O and 2-O patterns, and the corresponding length of TM-O bonds.

| 1-O pattern 2-O pattern | | | | | | H adsorption | |
| --- | --- | --- | --- | --- | --- | --- | --- |
|  | $\Delta G_{*NO3}$(eV) | *d*_TM-O_ (Å) | $\Delta G_{*NO3}$(eV) | *d*_TM-O1_ (Å) | *d*_TM-O2_ (Å) | $\Delta G_{*H}$(eV) | *d*_TM-H_ (Å) |
| Ti | **/** | **/** | -2.77 | 2.12 | 2.16 | -0.63 | 1.73 |
| V | -0.85 | 1.84 | -1.58 | 2.08 | 2.08 | +0.21 | 1.72 |
| Cr | **/** | **/** | -1.22 | 2.05 | 2.06 | -0.38 | 1.66 |
| Mn | **/** | **/** | -0.82 | 2.11 | 2.11 | -0.56 | 1.62 |
| Fe | **/** | **/** | -0.12 | 2.02 | 2.21 | -0.62 | 1.57 |
| Co | -0.38 | 1.82 | -0.68 | 1.96 | 2.22 | -0.55 | 1.51 |
| Ni | **/** | **/** | -0.38 | 2.08 | 2.09 | -0.40 | 1.50 |
| Cu | **/** | **/** | -0.08 | 2.06 | 2.07 | -0.75 | 1.51 |
| Zn |  |  |  |  |  |  |  |
| Zr | **/** | **/** | -2.94 | 2.23 | 2.23 | -1.04 | 1.87 |
| Nb | -0.70 | 2.00 | -1.55 | 2.23 | 2.23 | -0.55 | 1.80 |
| Mo | -0.75 | 1.92 | -1.67 | 2.16 | 2.16 | -0.60 | 1.78 |
| Ru | -0.30 | 1.94 | -0.79 | 2.11 | 2.11 | -0.71 | 1.64 |
| Rh | +0.06 | 2.00 | -0.51 | 2.11 | 2.16 | -0.61 | 1.59 |
| Pd | **/** | **/** | +0.17 | 2.20 | 2.20 | -0.20 | 1.80 |
| Ag |  |  |  |  |  |  |  |
| Cd |  |  |  |  |  |  |  |
| Hf | **/** | **/** | -2.37 | 2.19 | 2.19 | -1.34 | 1.85 |
| Ta | -1.46 | 1.94 | -2.18 | 2.19 | 2.19 | -1.18 | 1.85 |
| W | -1.35 | 1.93 | -2.22 | 2.15 | 2.15 | -1.22 | 1.80 |
| Re | **/** | **/** | -2.24 | 2.11 | 2.11 | -1.53 | 1.76 |
| Os | **/** | **/** | -1.41 | 2.09 | 2.09 | -1.26 | 1.67 |
| Ir | -0.45 | 1.95 | -1.00 | 2.10 | 2.11 | -1.26 | 1.60 |
| Pt | +0.60 | 2.06 | -0.12 | 2.16 | 2.16 | -0.46 | 1.58 |
| Au | +0.77 | 2.09 | / | / | / | -0.42 | 1.59 |

**Table S12.** The Gibbs free energy change of NO_3_^¯^ and H adsorption on TM-C_2_N_2_^b^@BPN *via* 1-O and 2-O patterns, and the corresponding length of TM-O bonds.

| 1-O pattern 2-O pattern | | | | | | H adsorption | |
| --- | --- | --- | --- | --- | --- | --- | --- |
|  | $\Delta G_{*NO3}$(eV) | *d*_TM-O_ (Å) | $\Delta G_{*NO3}$(eV) | *d*_TM-O1_ (Å) | *d*_TM-O2_ (Å) | $\Delta G_{*H}$(eV) | *d*_TM-H_ (Å) |
| Ti | **/** | **/** | -1.47 | 2.13 | 2.13 | -0.31 | 1.77 |
| V | **/** | **/** | -1.51 | 2.06 | 2.07 | -0.44 | 1.69 |
| Cr | **/** | **/** | -0.90 | 2.03 | 2.10 | -0.18 | 1.65 |
| Mn | **/** | **/** | -0.79 | 2.10 | 2.10 | -0.36 | 1.64 |
| Fe | **/** | **/** | -0.60 | 2.04 | 2.05 | +0.69 | 1.59 |
| Co | **/** | **/** | -0.56 | 2.00 | 2.00 | -0.41 | 1.48 |
| Ni | **/** | **/** | -0.18 | 2.05 | 2.05 | -0.05 | 1.48 |
| Cu | **/** | **/** | -0.22 | 2.04 | 2.05 | -0.48 | 1.51 |
| Zn |  |  |  |  |  |  |  |
| Zr | **/** | **/** | -1.86 | 2.23 | 2.25 | -0.55 | 1.90 |
| Nb | **/** | **/** | -1.92 | 2.18 | 2.19 | -0.79 | 1.81 |
| Mo | **/** | **/** | -0.84 | 2.21 | 2.35 | -0.63 | 1.73 |
| Ru | **/** | **/** | -0.54 | 2.08 | 2.10 | -0.80 | 1.60 |
| Rh | **/** | **/** | -0.31 | 2.06 | 2.21 | -0.75 | 1.55 |
| Pd | **/** | **/** | +0.37 | 2.25 | 2.25 | -0.13 | 1.61 |
| Ag |  |  |  |  |  |  |  |
| Cd |  |  |  |  |  |  |  |
| Hf | -1.26 | 1.97 | -2.23 | 2.19 | 2.21 | -0.93 | 1.89 |
| Ta | / | / | -2.50 | 2.18 | 2.18 | -0.84 | 1.82 |
| W | / | / | -1.32 | 2.05 | 2.05 | -0.95 | 1.73 |
| Re | / | / | -0.92 | 2.18 | 2.19 | -1.02 | 1.68 |
| Os | / | / | -0.36 | 2.22 | 2.23 | -1.02 | 1.62 |
| Ir | -0.17 | 1.96 | -0.69 | 2.04 | 2.21 | -1.30 | 1.57 |
| Pt | **/** | / | +0.06 | 2.19 | 2.20 | -0.13 | 1.58 |
| Au | -0.45 | 2.09 | **/** | **/** | **/** | -1.52 | 1.59 |

**Table S13.** The Gibbs free energy change of NO_3_^¯^ and H adsorption on TM-C_2_N_2_^c^@BPN *via* 1-O and 2-O patterns, and the corresponding length of TM-O bonds.

| 1-O pattern 2-O pattern | | | | | | H adsorption | |
| --- | --- | --- | --- | --- | --- | --- | --- |
|  | $\Delta G_{*NO3}$(eV) | *d*_TM-O_ (Å) | $\Delta G_{*NO3}$(eV) | *d*_TM-O1_ (Å) | *d*_TM-O2_ (Å) | $\Delta G_{*H}$(eV) | *d*_TM-H_ (Å) |
| Ti | **/** | **/** | -1.68 | 2.06 | 2.14 | -0.66 | 1.74 |
| V | **/** | **/** | -1.44 | 2.05 | 2.08 | -0.49 | 1.69 |
| Cr | **/** | **/** | -0.93 | 2.03 | 2.06 | -0.31 | 1.67 |
| Mn | **/** | **/** | -2.09 | 2.05 | 2.06 | -1.36 | 1.64 |
| Fe | +0.19 | 1.84 | -0.52 | 2.00 | 2.00 | -0.21 | 1.61 |
| Co | -1.03 | 1.80 | -1.87 | 1.98 | 1.98 | -0.07 | 1.53 |
| Ni | +0.22 | 1.80 | -0.59 | 1.95 | 1.95 | +0.10 | 1.50 |
| Cu | **/** | **/** | -0.37 | 2.06 | 2.06 | -0.54 | 1.50 |
| Zn |  |  |  |  |  |  |  |
| Zr | **/** | **/** | -1.91 | 2.20 | 2.27 | -0.63 | 1.88 |
| Nb | **/** | **/** | -1.91 | 2.20 | 2.23 | -1.04 | 1.83 |
| Mo | **/** | **/** | -0.94 | 2.20 | 2.20 | -0.69 | 1.77 |
| Ru | **/** | **/** | -0.56 | 2.15 | 2.15 | -0.08 | 1.62 |
| Rh | +0.07 | 1.95 | -0.75 | 2.13 | 2.13 | -0.37 | 1.62 |
| Pd | **/** | **/** | -0.21 | 2.19 | 2.20 | -0.13 | 1.61 |
| Ag | **/** | **/** | +0.17 | 2.29 | 2.31 | -0.27 | 1.63 |
| Cd |  |  |  |  |  |  |  |
| Hf | **/** | **/** | -2.13 | 2.16 | 2.23 | -0.84 | 1.87 |
| Ta | **/** | **/** | -1.89 | 2.20 | 2.20 | -1.16 | 1.82 |
| W | **/** | **/** | -1.29 | 2.17 | 2.17 | -1.10 | 1.77 |
| Re | **/** | **/** | -0.60 | 2.13 | 2.13 | -0.37 | 1.72 |
| Os | **/** | **/** | -0.99 | 2.15 | 2.15 | -2.13 | 1.66 |
| Ir | -0.42 | 1.94 | -1.19 | 2.13 | 2.13 | -1.14 | 1.64 |
| Pt | **/** | **/** | -0.61 | 2.16 | 2.16 | -1.06 | 1.62 |
| Au | +0.68 | 2.14 | **/** | **/** | **/** | -0.61 | 1.59 |

**Table S14.** The Gibbs free energy change of NO_3_^¯^ and H adsorption on TM-C_1_N_3_@BPN *via* 1-O and 2-O patterns, and the corresponding length of TM-O bonds.

| 1-O pattern 2-O pattern | | | | | | H adsorption | |
| --- | --- | --- | --- | --- | --- | --- | --- |
|  | $\Delta G_{*NO3}$(eV) | *d*_TM-O_ (Å) | $\Delta G_{*NO3}$(eV) | *d*_TM-O1_ (Å) | *d*_TM-O2_ (Å) | $\Delta G_{*H}$(eV) | *d*_TM-H_ (Å) |
| Ti | **/** | **/** | -1.29 | 2.13 | 2.16 | **/** | 2.24 |
| V | -0.68 | 1.86 | -1.16 | 2.08 | 2.11 | -0.43 | 1.66 |
| Cr | **/** | **/** | -1.18 | 2.06 | 2.06 | -0.04 | 1.61 |
| Mn | **/** | **/** | -0.86 | 2.09 | 2.11 | -0.58 | 1.64 |
| Fe | **/** | **/** | -0.84 | 2.04 | 2.09 | -2.38 | 1.77 |
| Co | **/** | **/** | -1.58 | 1.99 | 2.07 | -0.28 | 1.54 |
| Ni | **/** | **/** | -0.62 | 1.93 | 2.14 | -0.49 | 1.52 |
| Cu | **/** | **/** | -0.14 | 2.04 | 2.06 | -0.39 | 1.51 |
| Zn |  |  |  |  |  |  |  |
| Zr | **/** | **/** | -1.62 | 2.26 | 2.28 | -0.45 | 1.90 |
| Nb | **/** | **/** | -1.39 | 2.23 | 2.23 | -0.64 | 1.82 |
| Mo | **/** | **/** | -1.44 | 2.16 | 2.19 | -0.75 | 1.73 |
| Ru | **/** | **/** | -0.65 | 2.03 | 2.46 | -0.20 | 1.68 |
| Rh | **/** | **/** | -0.75 | 2.07 | 2.10 | -0.67 | 1.55 |
| Pd | **/** | **/** | -0.27 | 2.20 | 2.20 | -0.19 | 1.61 |
| Ag | **/** | **/** | +0.43 | 2.24 | 2.33 | +0.00 | 1.64 |
| Cd |  |  |  |  |  |  |  |
| Hf | **/** | **/** | -1.89 | 2.22 | 2.23 | -0.78 | 1.88 |
| Ta | **/** | **/** | -3.92 | 2.17 | 2.18 | -1.19 | 1.80 |
| W | **/** | **/** | -1.50 | 2.14 | 2.18 | -1.14 | 1.77 |
| Re | **/** | **/** | -3.28 | 2.10 | 2.39 | -1.12 | 1.69 |
| Os | **/** | **/** | -1.13 | 2.21 | 2.34 | -0.93 | 1.69 |
| Ir | **/** | **/** | -0.99 | 2.00 | 2.41 | -1.22 | 1.66 |
| Pt | -0.44 | 2.00 | -0.86 | 2.17 | 2.17 | -1.14 | 1.61 |
| Au | +0.85 | 2.09 | +0.95 | 2.16 | 2.44 | -0.27 | 1.59 |

**Table S15.** The Gibbs free energy change of NO_3_^¯^ and H adsorption on TM-N_4_@BPN *via* 1-O and 2-O patterns, and the corresponding length of TM-O bonds.

| 1-O pattern 2-O pattern | | | | | | H adsorption | |
| --- | --- | --- | --- | --- | --- | --- | --- |
|  | $\Delta G_{*NO3}$(eV) | *d*_TM-O_ (Å) | $\Delta G_{*NO3}$(eV) | *d*_TM-O1_ (Å) | *d*_TM-O2_ (Å) | $\Delta G_{*H}$(eV) | *d*_TM-H_ (Å) |
| Ti | **/** | **/** | -1.56 | 2.13 | 2.13 | -0.54 | 1.75 |
| V | **/** | **/** | -0.97 | 2.09 | 2.09 | -0.48 | 1.69 |
| Cr | **/** | **/** | -0.87 | 2.27 | 2.28 | -0.03 | 1.63 |
| Mn | **/** | **/** | -1.22 | 2.10 | 2.11 | -0.85 | 1.64 |
| Fe | **/** | **/** | +0.68 | 2.01 | 2.01 | +0.21 | 1.51 |
| Co | **/** | **/** | +1.20 | 2.22 | 2.22 | +0.20 | 1.46 |
| Ni | +1.76 | 2.03 | **/** | **/** | **/** | +0.99 | 1.50 |
| Cu | **/** | **/** | -0.02 | 2.06 | 2.09 | -0.51 | 1.50 |
| Zn |  |  |  |  |  |  |  |
| Zr | **/** | **/** | -1.77 | 2.28 | 2.28 | -0.69 | 1.91 |
| Nb | -0.60 | 1.98 | -1.50 | 2.23 | 2.24 | -0.65 | 1.83 |
| Mo | -0.61 | 2.02 | -1.09 | 2.16 | 2.17 | -0.50 | 1.75 |
| Ru | **/** | **/** | +0.12 | 2.13 | 2.13 | +0.07 | 1.63 |
| Rh | **/** | **/** | +0.70 | 2.12 | 2.12 | -0.06 | 1.55 |
| Pd |  |  |  |  |  |  |  |
| Ag | **/** | **/** | +0.35 | 2.34 | 2.34 | -0.19 | 1.63 |
| Cd |  |  |  |  |  |  |  |
| Hf | **/** | **/** | -3.04 | 2.24 | 2.24 | -1.20 | 1.89 |
| Ta | **/** | **/** | -2.50 | 2.17 | 2.17 | -1.04 | 1.80 |
| W | -1.58 | 1.99 | -2.33 | 2.16 | 2.16 | -1.10 | 1.76 |
| Re | **/** | **/** | -0.98 | 2.12 | 2.12 | -0.58 | 1.72 |
| Os | **/** | **/** | -0.10 | 2.12 | 2.12 | -0.35 | 1.65 |
| Ir | **/** | **/** | +0.66 | 2.12 | 2.12 | -0.26 | 1.60 |
| Pt | +2.06 | 2.22 | **/** | **/** | **/** | +0.67 | 1.59 |
| Au |  |  |  |  |  |  |  |

**Table S16.** The Gibbs free energy change of NO adsorption with different configurations (N-end, O-end and NO-side) on Cu(100) surface and TM-C*_x_*N*_y_*@BPN, which is calculated by Δ*G*_*NO_ = *G*_*NO_ - *G*_*_ - *G*_NO(g)_. The unit is eV.

|  | N-end | O-end | NO-side |  | N-end | O-end | NO-side |
| --- | --- | --- | --- | --- | --- | --- | --- |
| Cu(100) | -1.13 | +0.20 | -0.83 | Mo/C_1_N_3_ | -2.69 | -0.98 | / |
| Ti/C_2_N_2_^b^ | -1.55 | -0.72 | -1.37 | Ru/C_1_N_3_ | -3.45 | -0.75 | / |
| Ta/C_2_N_2_^b^ | -2.27 | -1.45 | -2.33 | W/C_1_N_3_ | -3.01 | -0.99 | -2.76 |
| Mo/C_2_N_2_^c^ | -0.99 | -0.99 | -1.95 | Ti/N_4_ | -1.84 | -0.88 | -1.72 |
| Ta/C_2_N_2_^c^ | -2.49 | -1.52 | -2.37 | V/N_4_ | -1.94 | -1.17 | -1.98 |
| W/C_2_N_2_^c^ | -3.02 | -1.30 | -2.46 | Cr/N_4_ | -2.45 | -0.93 | -1.88 |
| Re/C_2_N_2_^c^ | -2.51 | -0.58 | -1.89 | Nb/N_4_ | -1.73 | -1.09 | -2.06 |
| Ti/C_1_N_3_ | -1.78 | -1.14 | -1.63 | Mo/N_4_ | -2.34 | -0.67 | -2.27 |
| V/C_1_N_3_ | -2.26 | -1.04 | -1.73 | Ta/N_4_ | -2.48 | -1.59 | -2.69 |
| Cr/C_1_N_3_ | -2.60 | -0.85 | / | W/N_4_ | -2.75 | -1.54 | -3.26 |
| Nb/C_1_N_3_ | -2.08 | -1.09 | -1.99 | Re/N_4_ | -2.16 | -0.34 | -1.91 |

**Table S17.** The vibrational frequencies, zero-point energy (*ZPE*) and entropic contributions (*TS*) of intermediate species on Cu(100) surface at room temperature (298.15 K).

| Species | Vibrational frequencies  (cm^-1^) | | | | | | *ZPE*  (eV) | *TS*  (eV) |
| --- | --- | --- | --- | --- | --- | --- | --- | --- |
| *NO_3_ | 1516.99 | 1029.55 | 899.78 | 674.44 | 664.82 | 639.67 | 0.38 | 0.22 |
|  | 185.25 | 179.34 | 173.00 | 95.18 | 61.93 | 50.14 |  |  |
| *NO_3_H | 3652.24 | 1283.73 | 803.17 | 720.98 | 672.14 | 632.82 | 0.63 | 0.26 |
|  | 540.03 | 501.10 | 303.50 | 281.52 | 261.75 | 193.75 |  |  |
|  | 183.75 | 97.77 | 30.95 |  |  |  |  |  |
| *NO_2_ | 1075.69 | 1065.13 | 755.02 | 238.79 | 201.57 | 156.31 | 0.23 | 0.17 |
|  | 131.39 | 113.88 | 92.44 |  |  |  |  |  |
| *NO_2_H | 3704.40 | 1664.49 | 644.13 | 566.51 | 370.52 | 281.80 | 0.48 | 0.22 |
|  | 233.58 | 126.77 | 126.06 | 77.31 | 58.84 | 44.34 |  |  |
| *NO | 1319.51 | 362.74 | 358.79 | 254.83 | 117.75 | 106.92 | 0.16 | 0.13 |
| *NOH | 3630.76 | 1266.91 | 767.87 | 431.95 | 419.06 | 272.47 | 0.44 | 0.18 |
|  | 148.73 | 143.27 | 34.53 |  |  |  |  |  |
| *N | 494.90 | 486.69 | 328.68 |  |  |  | 0.08 | 0.03 |
| *NH | 3405.50 | 773.16 | 762.27 | 402.48 | 385.01 | 379.31 | 0.38 | 0.05 |
| *NH_2_ | 3520.20 | 3407.94 | 1482.38 | 665.48 | 633.60 | 570.11 | 0.69 | 0.08 |
|  | 451.08 | 363.99 | 112.44 |  |  |  |  |  |
| *NH_3_ | 3512.28 | 3477.39 | 3358.29 | 1603.33 | 1595.18 | 1120.39 | 1.02 | 0.16 |
|  | 550.52 | 532.13 | 328.45 | 178.22 | 94.29 | 70.57 |  |  |

**Table S18.** The Gibbs free energy changes towards NO_3_RR for each elementary step on Cu(100) surface and TM-C_x_N_y_@BPN through the N-side pathway. The unit is eV.

|  | $\boldsymbol{\Delta}\boldsymbol{G}_{\mathbf{1}}$ | $\boldsymbol{\Delta}\boldsymbol{G}_{\mathbf{2}}$ | $\boldsymbol{\Delta}\boldsymbol{G}_{\mathbf{3}}$ | $\boldsymbol{\Delta}\boldsymbol{G}_{\mathbf{4}}$ | $\boldsymbol{\Delta}\boldsymbol{G}_{\mathbf{5}}$ | $\boldsymbol{\Delta}\boldsymbol{G}_{\mathbf{6}}$ | $\boldsymbol{\Delta}\boldsymbol{G}_{\mathbf{7}}$ | $\boldsymbol{\Delta}\boldsymbol{G}_{\mathbf{8}}$ | $\boldsymbol{\Delta}\boldsymbol{G}_{\mathbf{9}}$ | $\boldsymbol{\Delta}\boldsymbol{G}_{\mathbf{10}}$ | $\boldsymbol{\Delta}\boldsymbol{G}_{\boldsymbol{11}}$ |
| --- | --- | --- | --- | --- | --- | --- | --- | --- | --- | --- | --- |
| Ta/C_2_N_2_^b^ | -2.50 | -0.02 | -1.45 | -0.37 | +0.01 | -1.08 | -0.47 | -1.11 | -0.59 | +1.48 | +0.32 |
| Mo/C_2_N_2_^c^ | -0.94 | +0.09 | -1.78 | -1.15 | -0.16 | -0.61 | -0.42 | -1.82 | -0.68 | +1.54 | +0.17 |
| V/N_4_ | -0.97 | +0.21 | -2.35 | +0.49 | -1.35 | +0.32 | -1.80 | -1.16 | -0.79 | +1.45 | +0.19 |
| Nb/N_4_ | -1.50 | -0.93 | -0.76 | -1.47 | +0.59 | -1.01 | -0.34 | -1.70 | -0.43 | +1.32 | +0.45 |
| Ta/N_4_ | -2.50 | -0.39 | -1.29 | -0.50 | -0.00 | -1.16 | -0.10 | -1.26 | -0.56 | +1.94 | +0.06 |
| W/N_4_ | -2.33 | -0.21 | -1.30 | -0.12 | -1.29 | -0.31 | -0.28 | -1.93 | -0.15 | +1.93 | +0.23 |

**Table S19.** The Gibbs free energy changes towards NO_3_RR for each elementary step on Cu(100) surface and TM-C_x_N_y_@BPN through the N-end pathway. The unit is eV.

|  | $\boldsymbol{\Delta}\boldsymbol{G}_{\mathbf{1}}$ | $\boldsymbol{\Delta}\boldsymbol{G}_{\mathbf{2}}$ | $\boldsymbol{\Delta}\boldsymbol{G}_{\mathbf{3}}$ | $\boldsymbol{\Delta}\boldsymbol{G}_{\mathbf{4}}$ | $\boldsymbol{\Delta}\boldsymbol{G}_{\mathbf{5}}$ | $\boldsymbol{\Delta}\boldsymbol{G}_{\mathbf{6}}$ | $\boldsymbol{\Delta}\boldsymbol{G}_{\mathbf{7}}$ | $\boldsymbol{\Delta}\boldsymbol{G}_{\mathbf{8}}$ | $\boldsymbol{\Delta}\boldsymbol{G}_{\mathbf{9}}$ | $\boldsymbol{\Delta}\boldsymbol{G}_{\mathbf{10}}$ | $\boldsymbol{\Delta}\boldsymbol{G}_{\boldsymbol{11}}$ |
| --- | --- | --- | --- | --- | --- | --- | --- | --- | --- | --- | --- |
| Cu(100) | -0.16 | -0.02 | -2.00 | +0.26 | -1.54 | -0.23 | -1.69 | -0.83 | -0.39 | -0.63 | +0.62 |
| Ti/C_2_N_2_^b^ | -1.47 | +0.74 | -2.37 | +0.60 | -1.06 | +0.53 | +0.13 | -2.40 | -1.71 | -0.01 | +1.25 |
| Ta/C_2_N_2_^b^ | -2.50 | -0.02 | -1.45 | -0.37 | +0.07 | +0.18 | -0.20 | -2.17 | -1.12 | +0.81 | +0.99 |
| Mo/C_2_N_2_^c^ | -0.94 | +0.09 | -1.78 | -1.15 | +0.81 | -0.37 | -1.52 | -0.06 | -2.36 | +0.17 | +1.36 |
| Ta/C_2_N_2_^c^ | -1.89 | -1.02 | -0.70 | -0.59 | -0.29 | +0.05 | -0.24 | -2.23 | -0.96 | +0.76 | +1.33 |
| W/C_2_N_2_^c^ | -1.29 | -0.27 | -1.51 | -1.46 | -0.49 | +0.30 | -1.04 | -0.03 | -1.98 | +0.57 | +1.43 |
| Re/C_2_N_2_^c^ | -0.60 | -0.01 | -1.94 | +0.80 | -2.75 | +0.21 | -1.00 | -1.03 | -0.51 | +0.13 | +0.94 |
| Ti/C_1_N_3_ | -1.29 | +0.49 | -2.20 | +0.69 | -1.46 | +0.39 | +0.19 | -2.45 | -1.36 | -0.15 | +1.38 |
| V/C_1_N_3_ | -1.16 | +0.50 | -2.22 | +0.70 | -2.08 | +0.32 | -0.99 | -1.30 | -0.88 | +0.03 | +1.31 |
| Cr/C_1_N_3_ | -1.18 | -0.01 | -1.58 | +0.74 | -2.56 | +0.34 | -1.06 | -0.93 | -0.41 | +0.02 | +0.88 |
| Nb/C_1_N_3_ | -1.39 | -0.14 | -1.62 | -0.58 | -0.35 | +0.16 | -0.57 | -1.83 | -0.94 | +0.42 | +1.07 |
| Mo/C_1_N_3_ | -1.44 | +0.23 | -1.96 | -1.24 | -0.28 | +1.20 | -1.85 | -1.28 | -0.67 | +0.32 | +1.21 |
| Ru/C_1_N_3_ | -0.65 | +0.89 | -2.65 | +0.48 | -3.53 | +0.34 | -1.37 | -0.28 | -0.01 | -0.35 | +1.36 |
| W/C_1_N_3_ | -1.50 | -0.12 | -1.63 | -0.42 | -1.36 | +0.34 | -1.08 | -1.46 | -0.58 | +0.56 | +1.48 |
| Ti/N_4_ | -1.56 | -1.54 | -0.03 | -0.24 | -0.46 | +0.40 | +0.06 | -2.46 | -1.38 | +0.07 | +1.38 |
| V/N_4_ | -0.97 | +0.21 | -2.35 | +0.49 | -1.31 | +0.07 | -0.94 | -1.41 | -0.95 | +0.05 | +1.34 |
| Cr/N_4_ | -0.87 | -0.01 | -1.57 | +0.29 | -2.29 | +0.41 | -1.14 | -0.83 | +0.16 | -0.71 | +0.79 |
| Nb/N_4_ | -1.50 | -0.93 | -0.76 | -1.47 | +0.93 | -0.16 | -0.71 | -1.85 | -0.78 | +0.33 | +1.13 |
| Mo/N_4_ | -1.09 | -0.08 | -1.62 | -0.22 | -1.33 | -0.10 | -1.11 | -1.29 | -0.14 | +0.16 | +1.06 |
| Ta/N_4_ | -2.50 | -0.39 | -1.29 | -0.50 | +0.20 | -0.18 | -0.10 | -2.24 | -0.70 | +0.96 | +0.97 |
| W/N_4_ | -2.33 | -0.21 | -1.30 | -0.12 | -0.78 | -0.15 | -0.90 | -1.57 | -0.35 | +1.04 | +0.91 |
| Re/N_4_ | -0.98 | -0.21 | -1.56 | -0.23 | -1.18 | -0.12 | -1.33 | -1.02 | -0.36 | +0.90 | +0.30 |

**Table S20.** Twelve input features related to elements, structure, electronic and adsorption properties.

| Symbol | Description |
| --- | --- |
| *R*_M_ | atomic radius of active sites |
| *L*_M_ | electron shell number of active sites |
| *N*_e_ | valence electron number of active sites |
| *θ*_d_ | *d*-electron number of active sites |
| *χ*_M_ | electronegativity of active sites |
| IE | the first ionization energy of active sites |
| EA | the electron affinity of active sites |
| *Q*_M_ | charge transfer from active sites to the substrates |
| *ε*_d_ | *d*-band center of active sites |
| ∆*E*_O*_ | the atomic O adsorption energy |
| ∆*E*_N*_ | the atomic N adsorption energy |
| ∆*G*_NO3*_ | the NO_3_^−^ adsorption free energy |

**Table S21.** Mathematical operators employed in symbolic regression fitness in this work.

| Mathematical operators | Description |
| --- | --- |
| + | addition, arity=2 |
| - | subtraction, arity=2 |
| × | multiplication, arity=2 |
| / | division, arity=2 |
| √ | square root, arity=1 |
| ^2^ | square power, arity=1 |
| ^3^ | cube root, arity=1 |

**Table S22.** Comparison for NO_3_RR of this work and other catalytic systems.

| Catalyst | Δ*G*_NO*_  (eV) | *U*_L_  (V) | Potential-determining step | Reference |
| --- | --- | --- | --- | --- |
| Ti/N_4_@BPN | -1.56 | -0.40 | *NO → *NOH | this work |
| Mo/N_4_@BPN | -1.09 | -0.16 | *NH_2_ → *NH_3_ | this work |
| Fe-N_4_/C | -0.13 | -0.53 | *NO → *NOH | [10] |
| Os-N_4_/C | -0.83 | -0.42 | *N → *NH | [10] |
| Os/GDY | -1.57 | -0.37 | *NO_3_ → *NO_3_H | [25] |
| Cr/GY | -0.88 | -0.23 | *NO_3_ → *NO_3_H | [21] |
| Mo/GY | -1.43 | -0.28 | *NO_3_ → *NO_3_H | [21] |
| Nb/PP | -1.62 | -0.24 | *NO_2_ → *NO_2_H | [26] |
| Ta/PP | -1.57 | -0.28 | *NH_2_ → *NH_3_ | [26] |
| W/PP | -0.82 | -0.33 | *NH_2_ → *NH_3_ | [26] |
| Ti/g-CN | -1.54 | -0.39 | *NO → *NOH | [9] |
| Zr/g-CN | -1.90 | -0.41 | *NH_2_ → *NH_3_ | [9] |
| Ru/g-C_3_N_4_ (triazine) | -2.44 | -0.34 | *NO → *NOH | [27] |
| Ti/g-C_3_N_4_  (tri-s-triazine) | -3.11 | -0.30 | *NO → *NOH | [28] |
| Zr/g-C_2_N | -1.28 | -0.28 | *NO → *NOH | [29] |
| Hf/g-C_2_N | -1.47 | -0.27 | *NO → *NOH | [29] |
| V/*h*-BP | -0.99 | -0.22 | *NO_2_H → *NO | [30] |
| V/GaN | -1.08 | -0.39 | *NH_2_ → *NH_3_ | [31] |
| Al_L_/Co_3_O_4_ | -1.79 | -0.25 | *NO → *NOH | [32] |

**Table S23.** The calculated total energies and the Gibbs free energies with correction of vibrational frequencies of relevant molecules with and without solvent effect.

| Species | Pressure  (bar) | Temperature  (K) | *E*_DFT_  (eV) | *E*_DFT+Sol_  (eV) | *G*_DFT_  (eV) | *G*_DFT+Sol_  (eV) |
| --- | --- | --- | --- | --- | --- | --- |
| HNO_3_ (g) | 1 | 298.15 | -28.63 | -28.96 | -28.64 | -28.98 |
| H_2_ (g) | 1 | 298.15 | -6.69 | -6.69 | -6.74 | -6.74 |
| H_2_O (l) | 0.035 | 298.15 | -14.22 | -14.54 | -14.22 | -14.55 |
| NH_3_ (g) | 1 | 298.15 | -19.52 | -19.70 | -19.10 | -19.29 |
| N_2_ (g) | 1 | 298.15 | -16.60 | -16.59 | -16.95 | -16.94 |
| NO (g) | 1 | 298.15 | -12.29 | -12.30 | -12.70 | -12.71 |
| NO_2_ (g) | 1 | 298.15 | -18.38 | -18.40 | -18.77 | -18.79 |


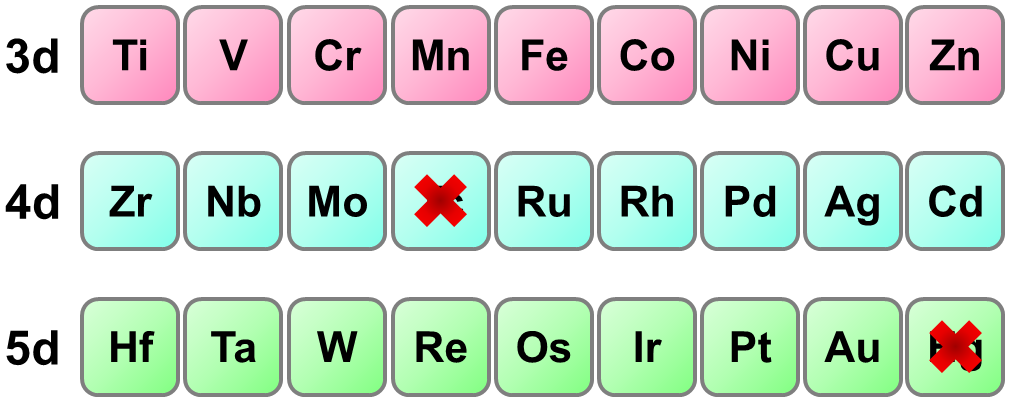


**Figure S1.** The screened TM atoms (from Ti to Au) in this work.


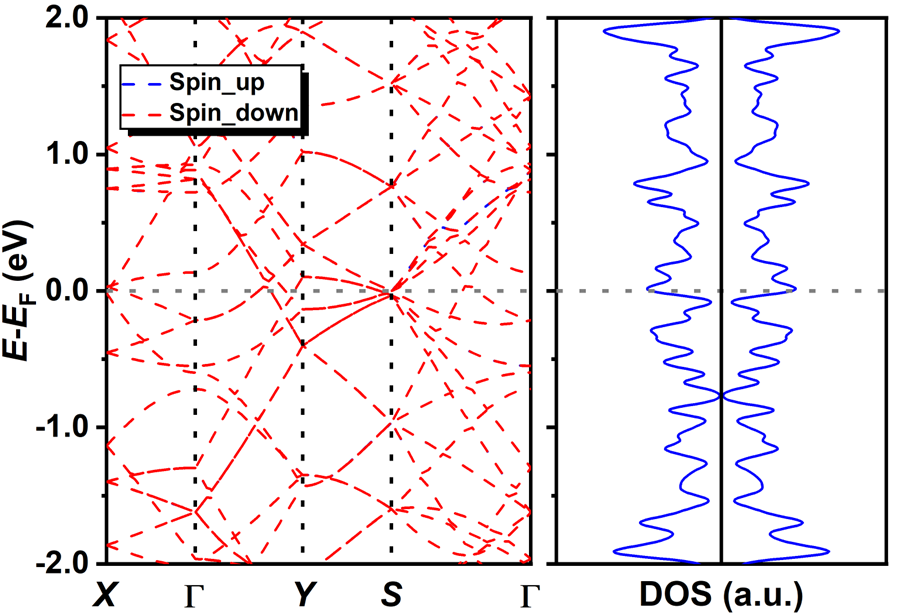


**Figure S2.** Band structure and DOS of pristine BPN.


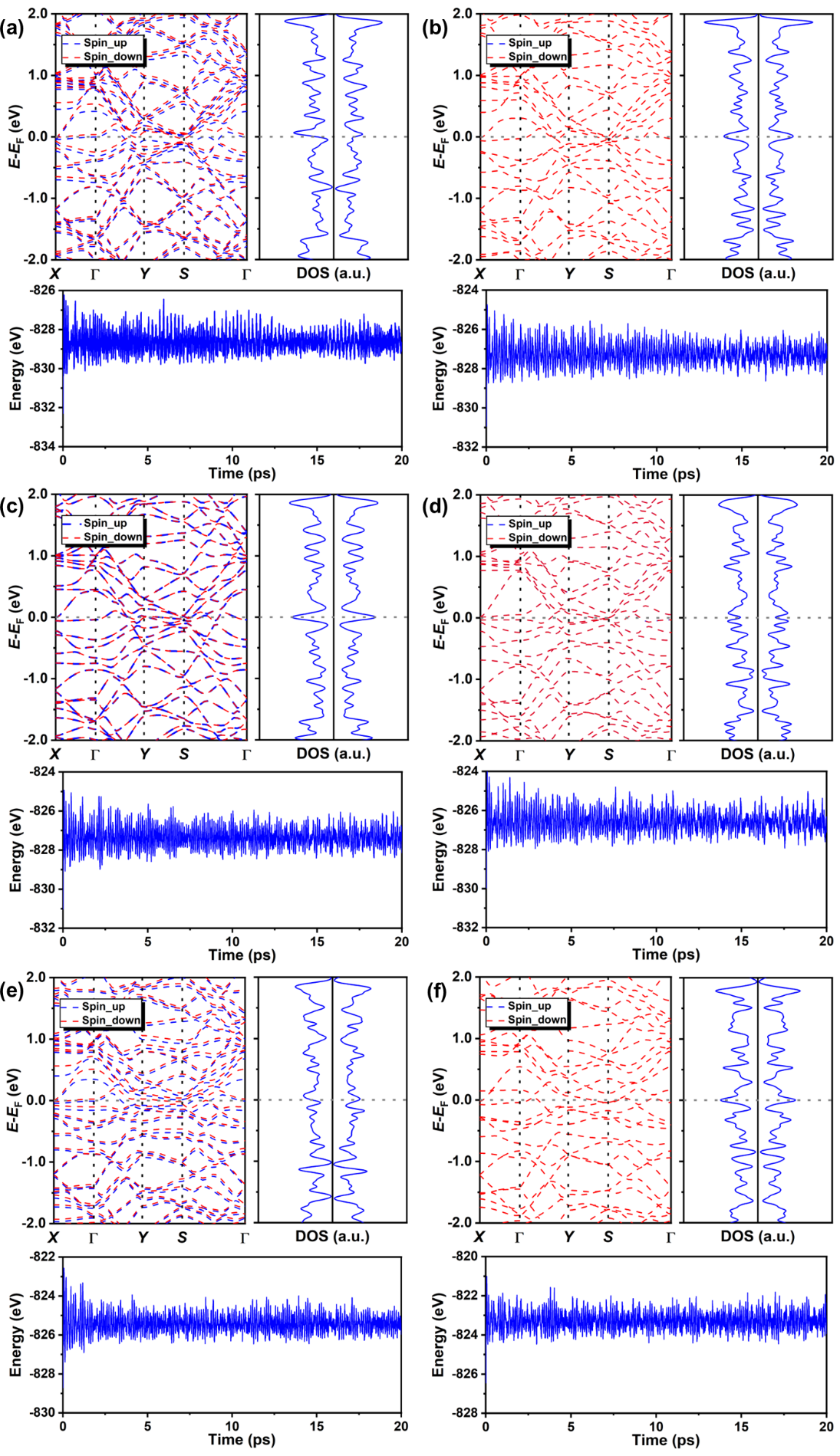


**Figure S3.** Electronic structures and fluctuations of energy with corresponding structures after AIMD simulations at room temperature for (a) C_3_N_1_@BPN, (b) C_2_N_2_^a^@BPN, (c) C_2_N_2_^b^@BPN, (d) C_2_N_2_^c^@BPN, (e) C_1_N_3_@BPN and (f) N_4_@BPN. respectively.


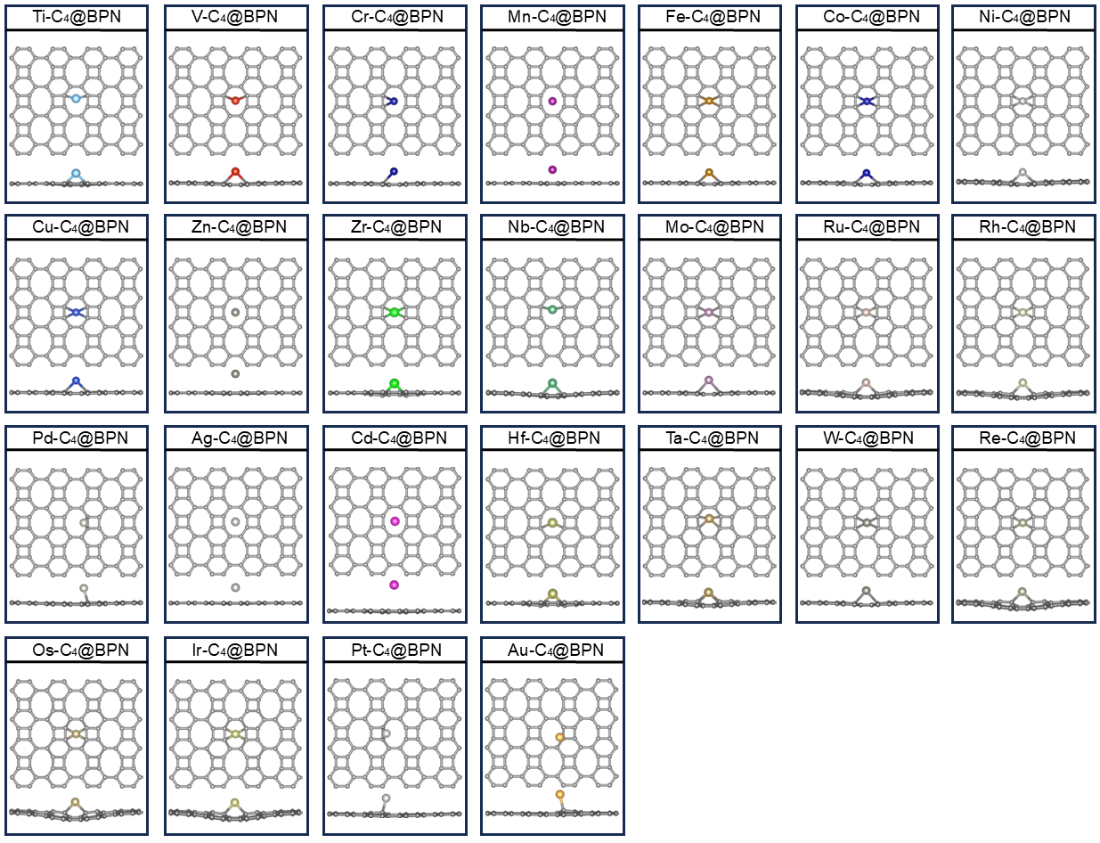


**Figure S4.** Optimized configurations of TM-C_4_@BPN.


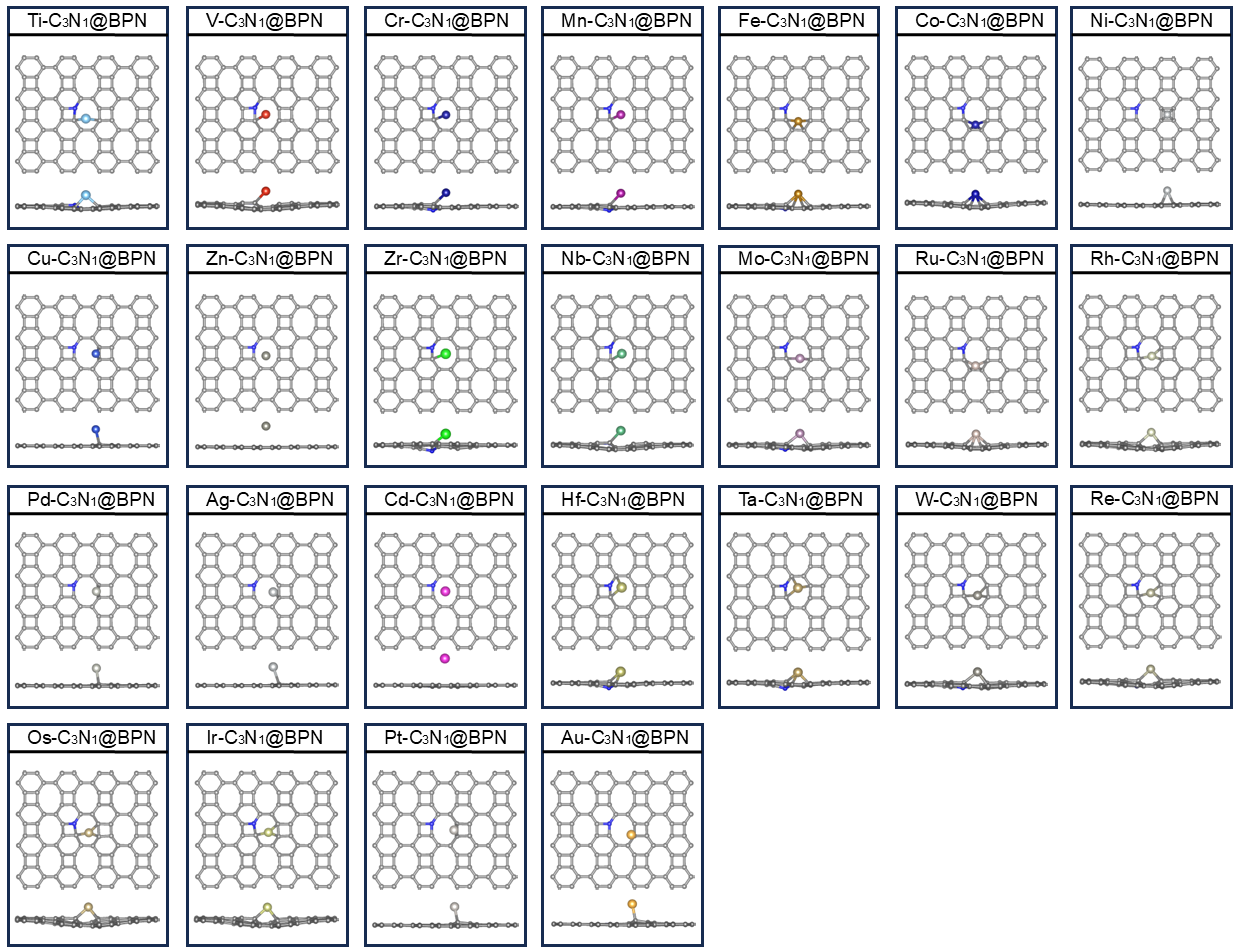


**Figure S5.** Optimized configurations of TM-C_3_N_1_@BPN.


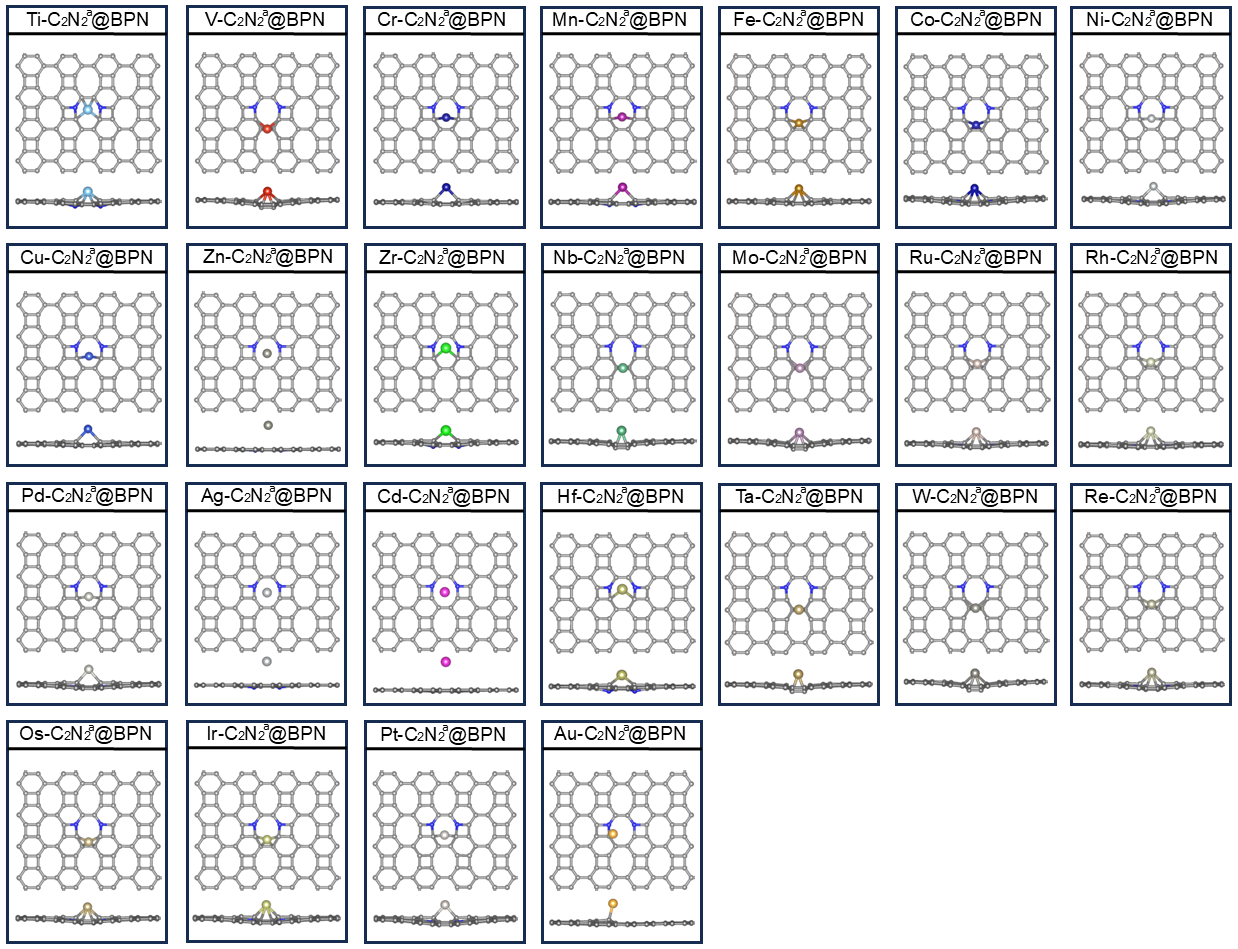


**Figure S6.** Optimized configurations of TM-C_2_N_2_^a^@BPN.


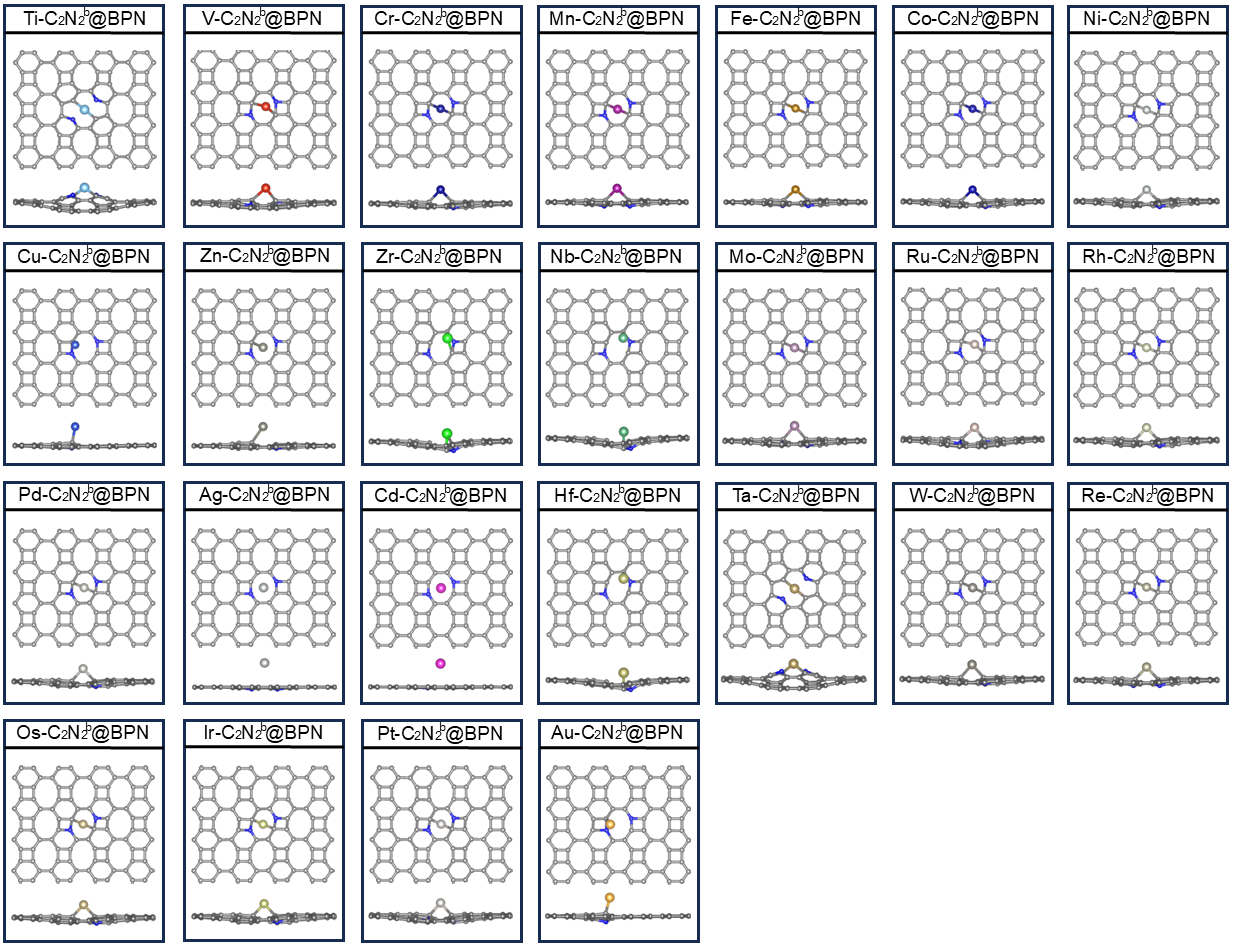


**Figure S7.** Optimized configurations of TM-C_2_N_2_^b^@BPN.


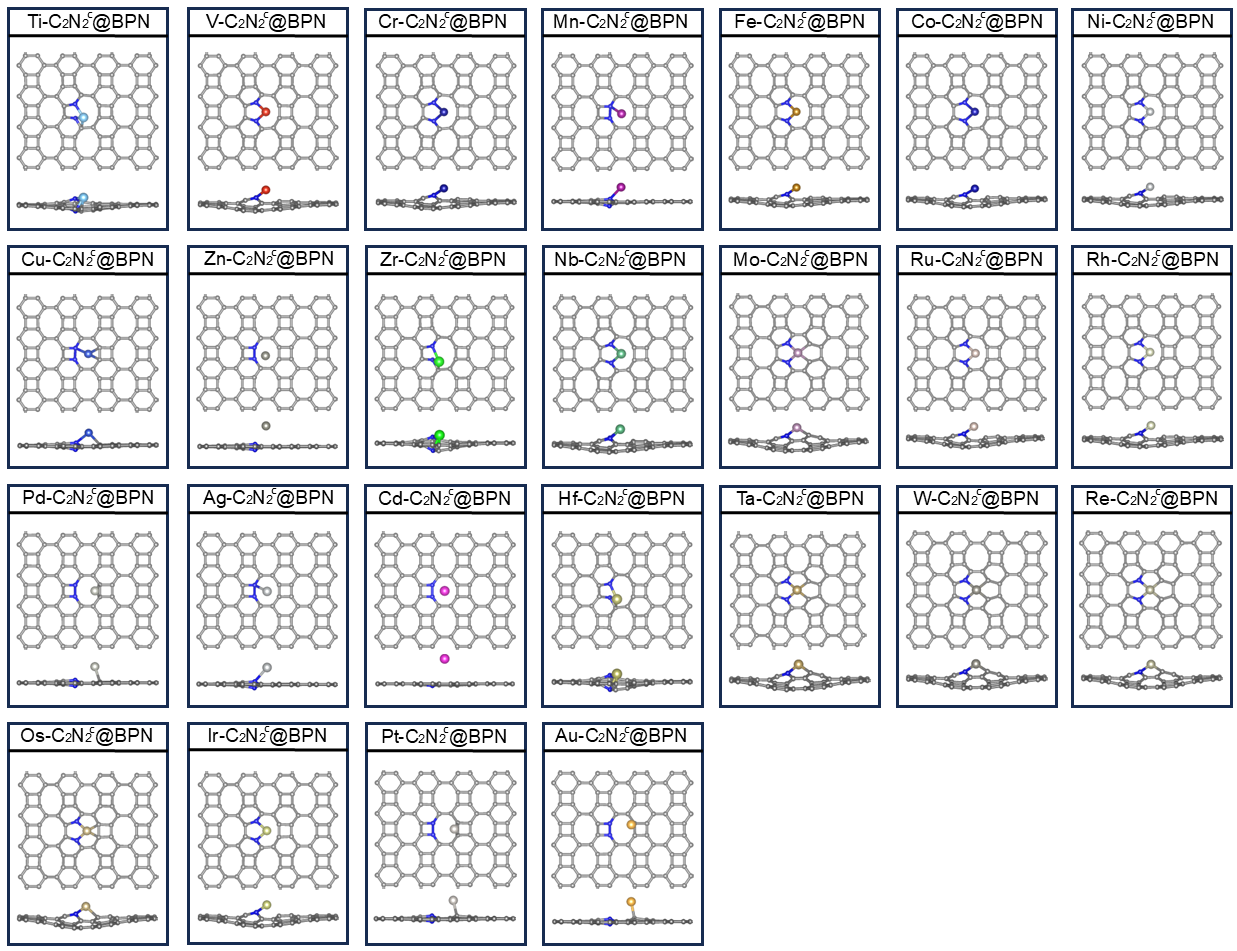


**Figure S8.** Optimized configurations of TM-C_2_N_2_^c^@BPN.


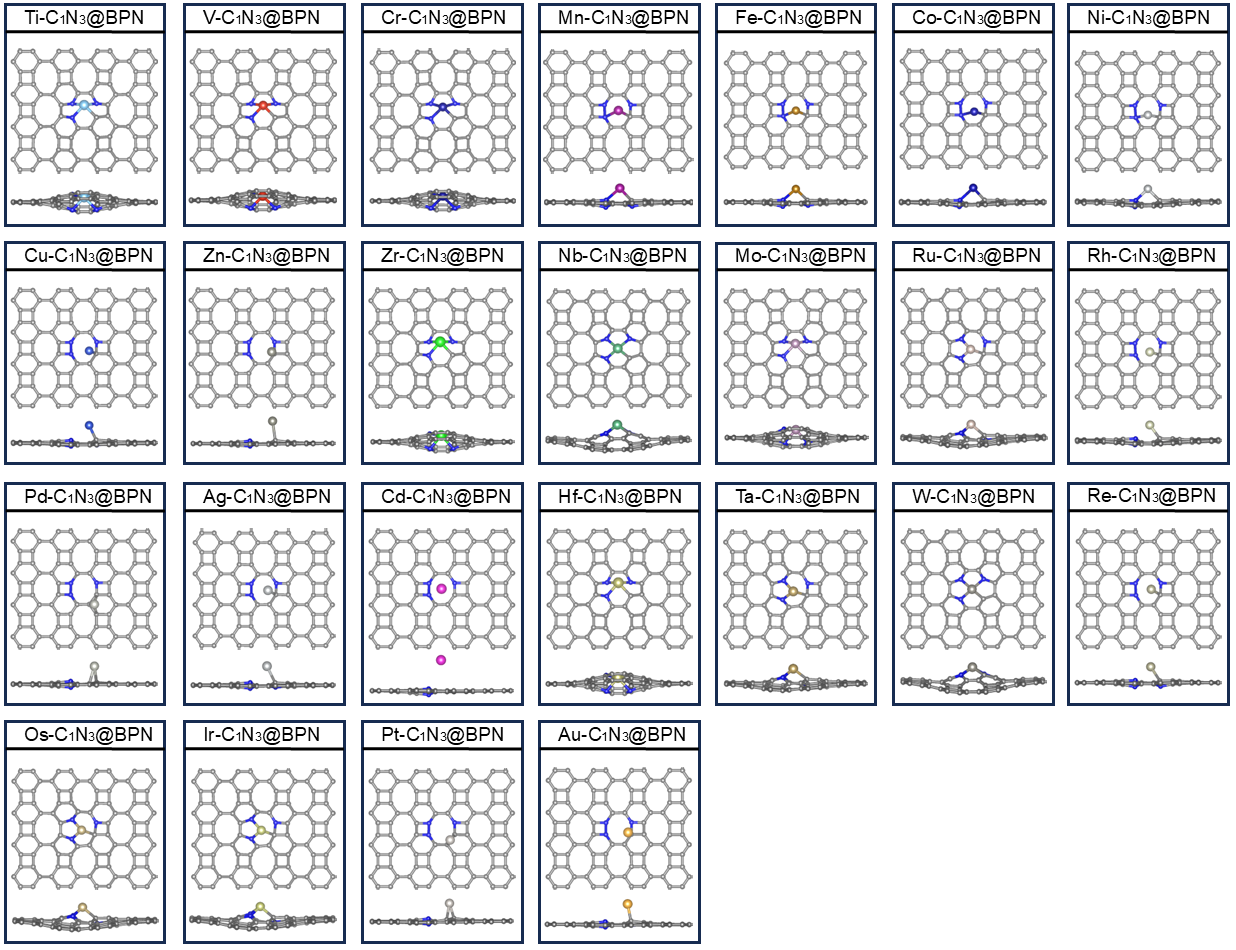


**Figure S9.** Optimized configurations of TM-C_1_N_3_@BPN.


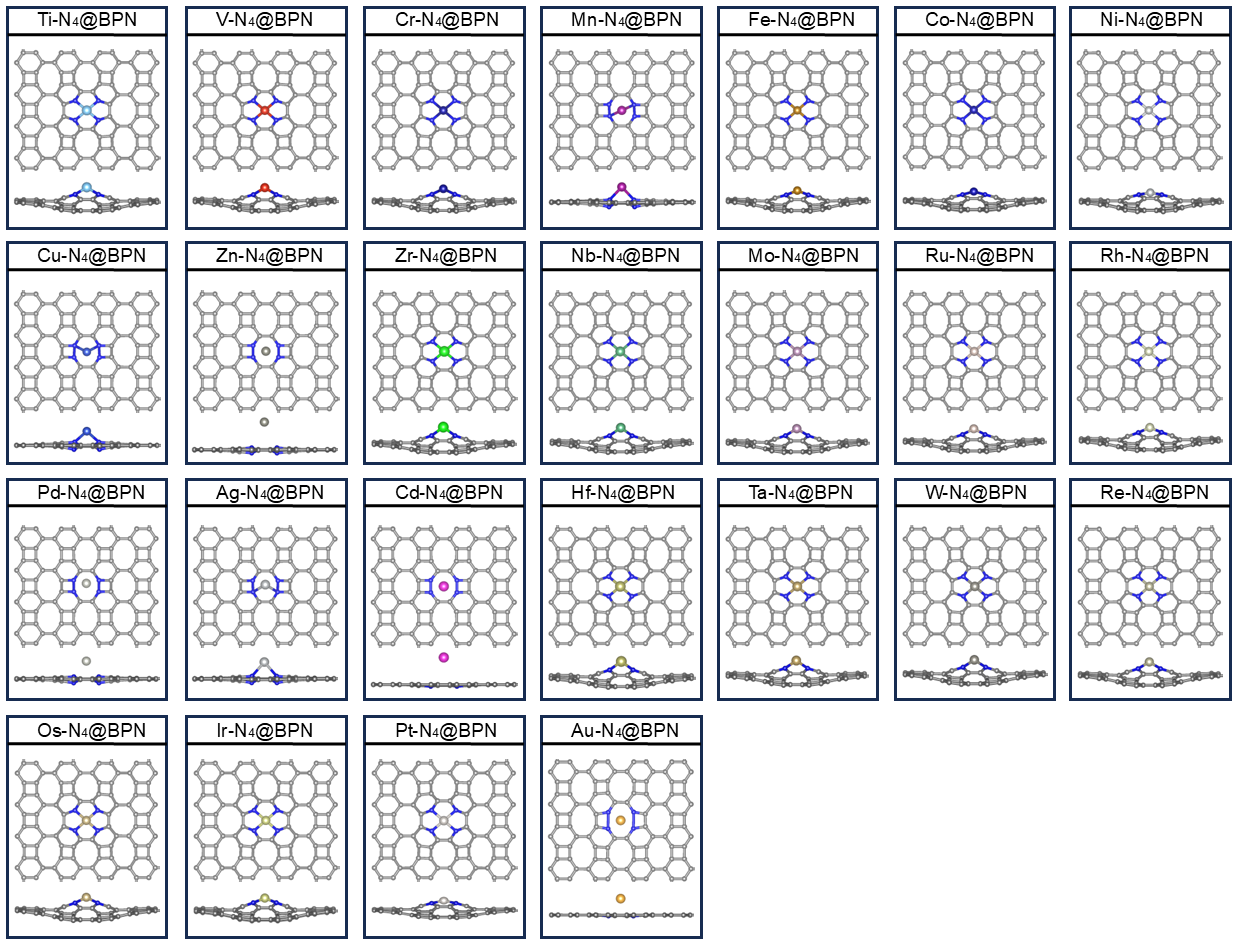


**Figure S10.** Optimized configurations of TM-N_4_@BPN.


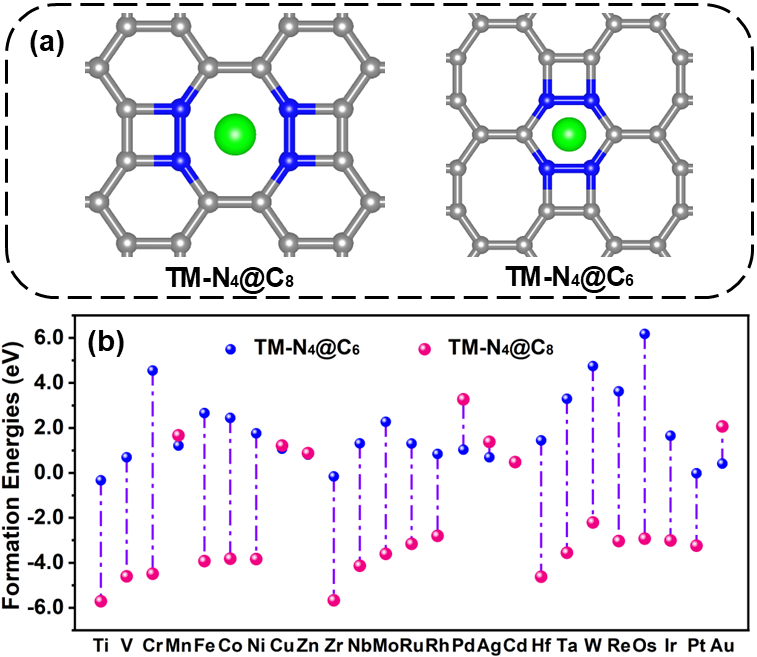


**Figure S11.** (a) Schematic configurations of TM single atoms embedded in six-atom ring and eight-atom ring with N_4_ coordination environments, namely TM-N_4_@C_6_ and TM-N_4_@C_8_. (b) Formation energies of TM-N_4_@C_6_ and TM-N_4_@C_8_.


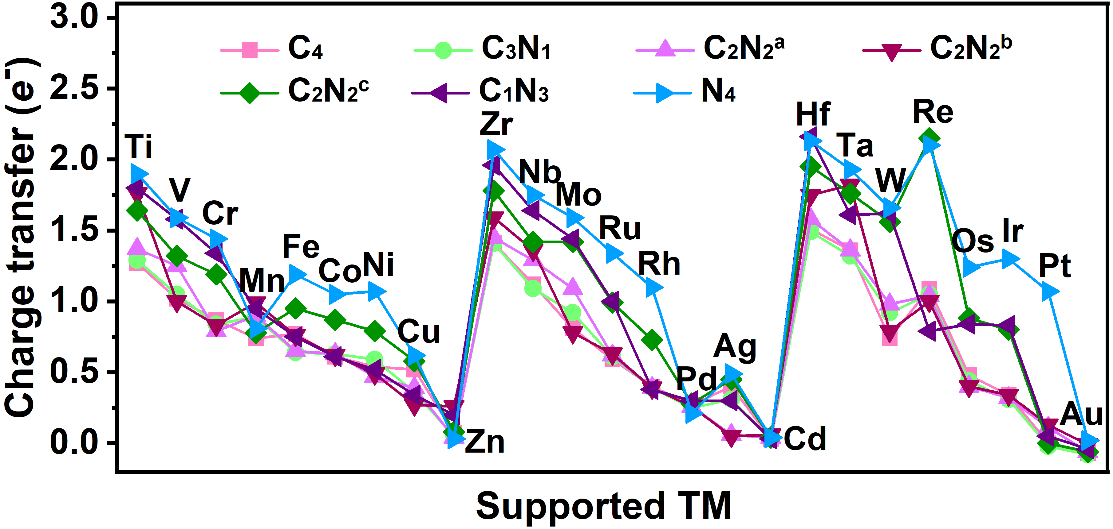


**Figure S12.** Charge transfer *δ* as a function of atomic number.


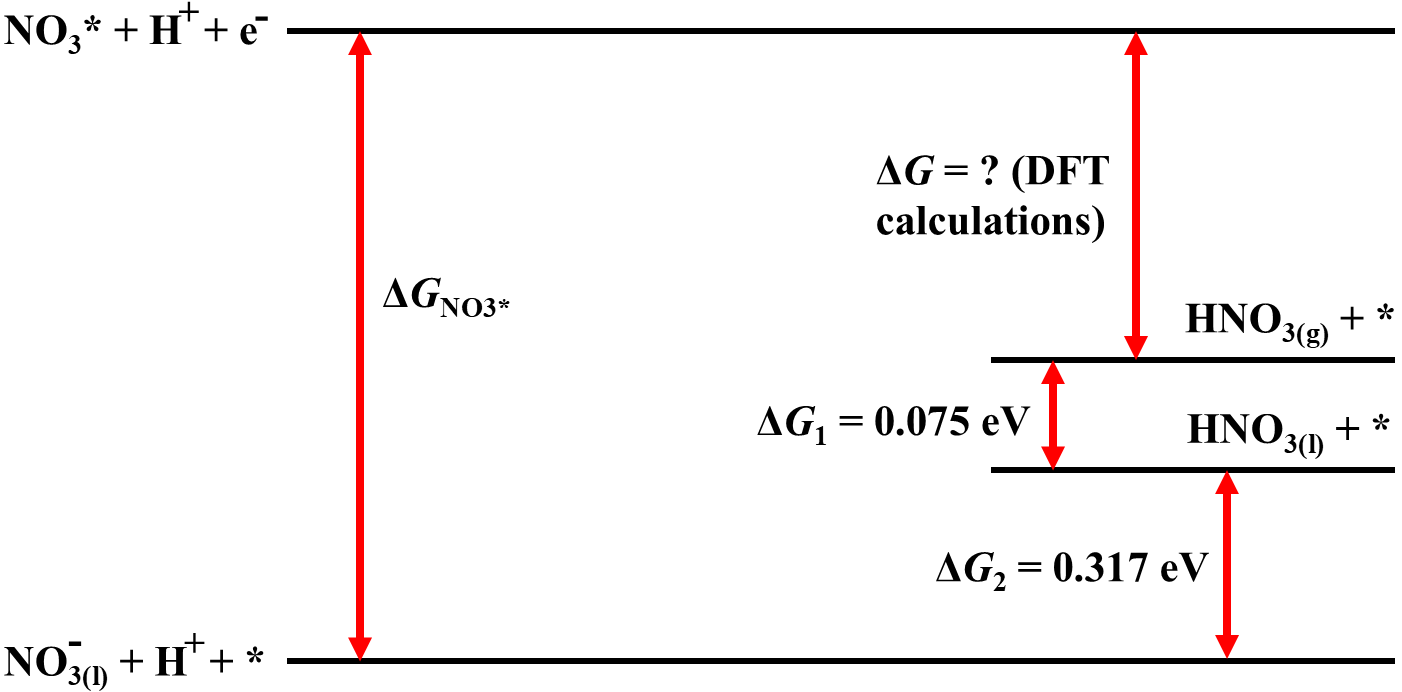


**Figure S13.** The thermodynamic cycle for the Gibbs free energy of NO_3_^¯^ adsorption in the aqueous phase.


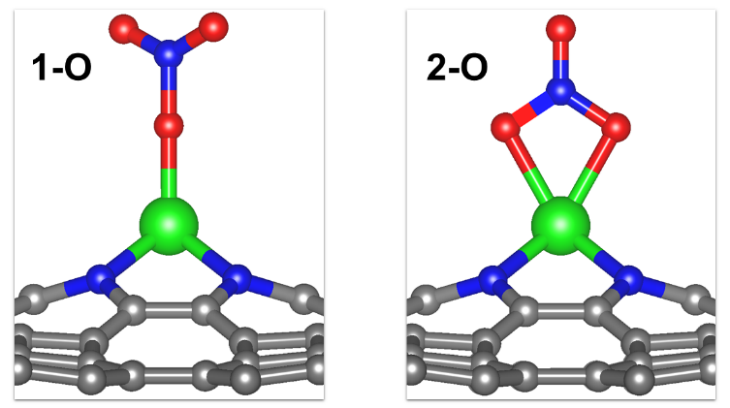


**Figure S14.** The 1-O pattern (one oxygen atom adsorbed on catalytic sites) and 2-O pattern (two oxygen atoms adsorbed on catalytic sites) configurations of NO_3_^¯^ adsorption. Nb-N_4_@BPN is taken as a representative sample.


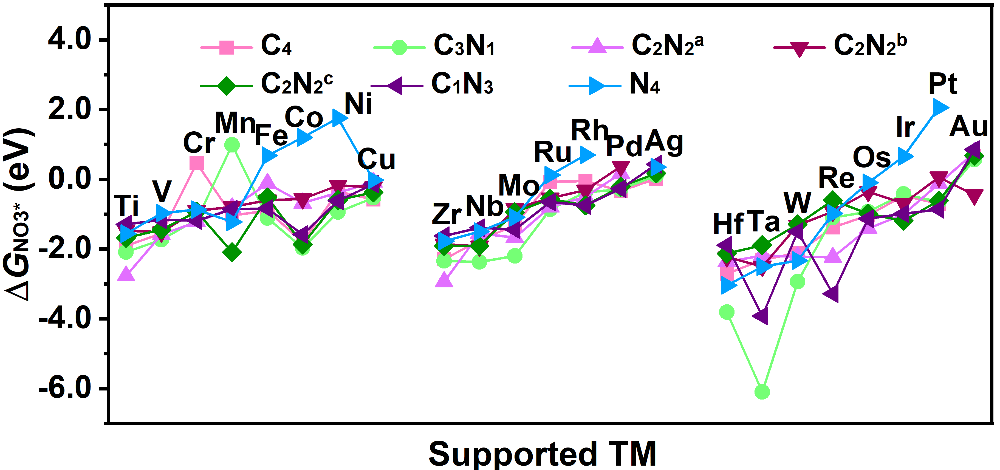


**Figure S15.** Adsorption free energies of NO_3_^¯^ as a function of atomic number.


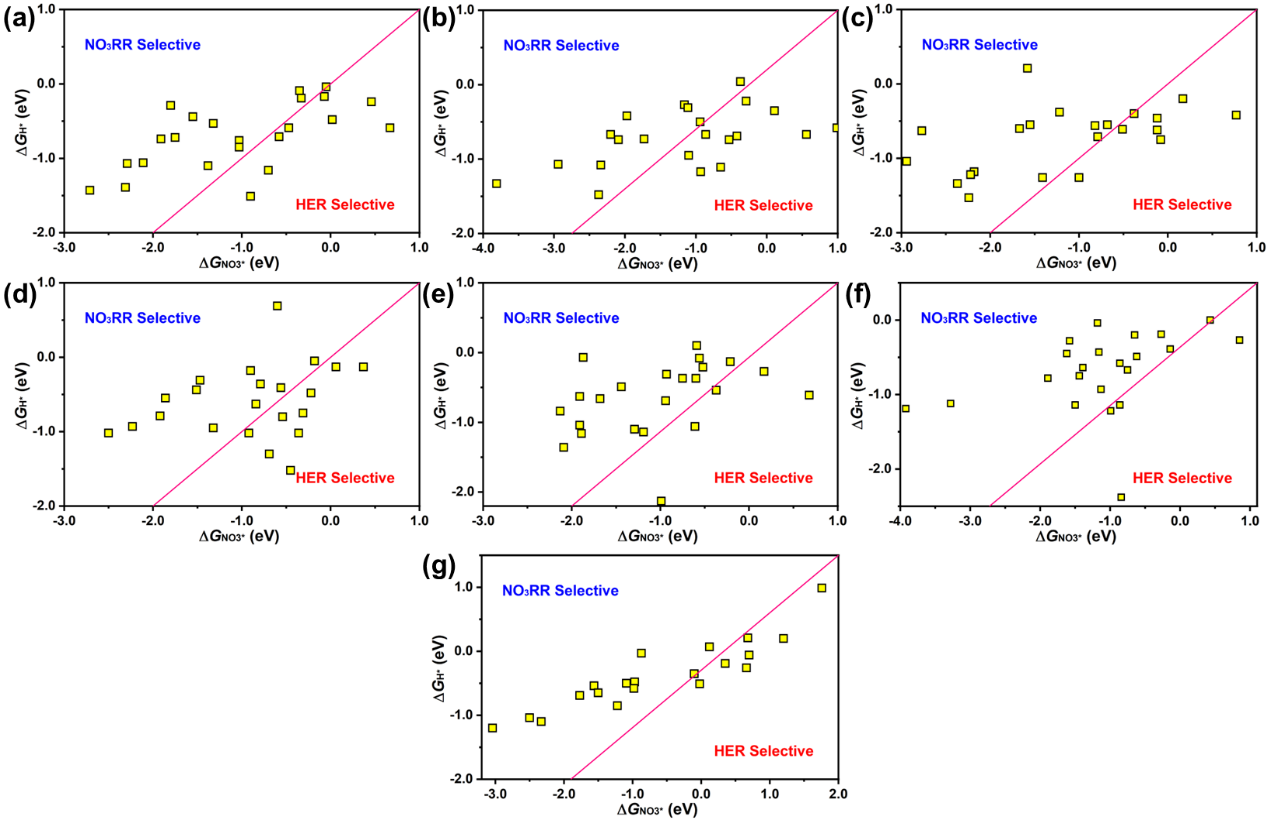


**Figure S16.** Gibbs free energy changes of initial protonation of NO_3_RR *versus* HER on (a) C_4_@BPN, (b) C_3_N_1_@BPN, (c) C_2_N_2_^a^@BPN, (d) C_2_N_2_^b^@BPN, (e) C_2_N_2_^c^@BPN and (f) C_1_N_3_@BPN and (g) N_4_@BPN, respectively.


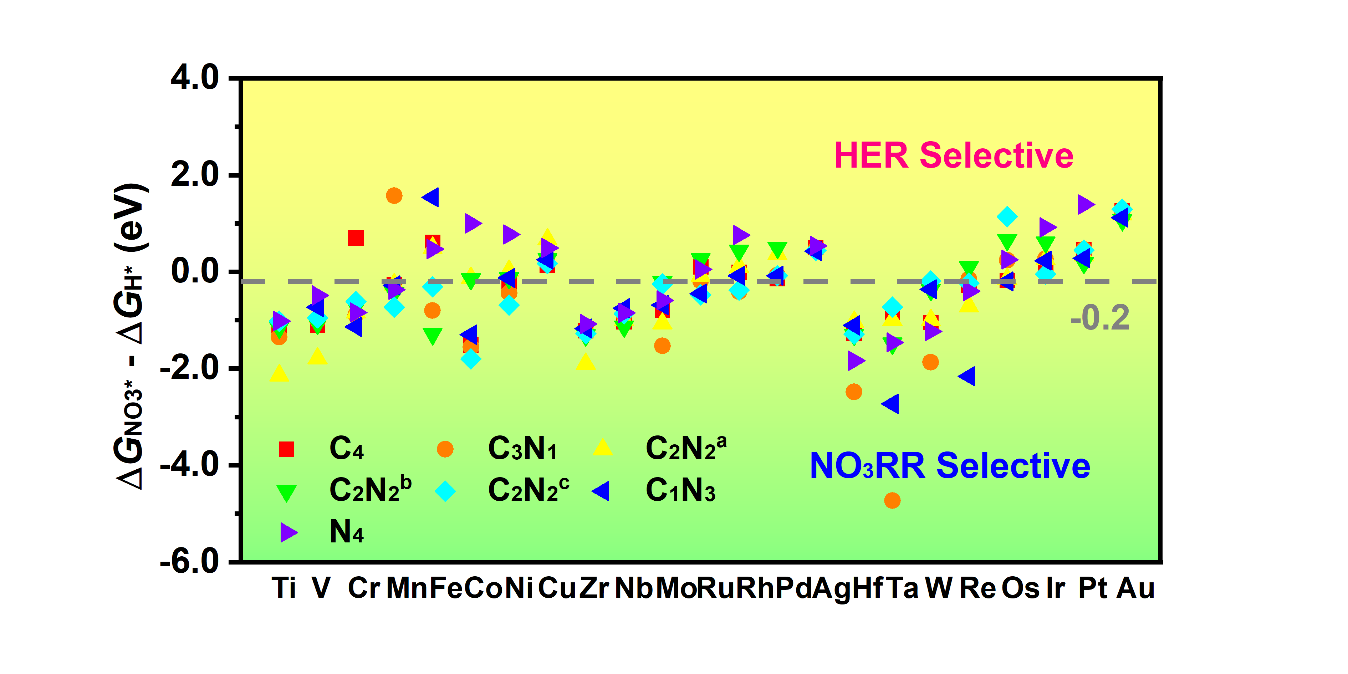


**Figure S17.** Scatter plot for Δ*G*_NO3*_ - Δ*G*_H*_ *versus* different TM-C_x_N_y_@BPN.


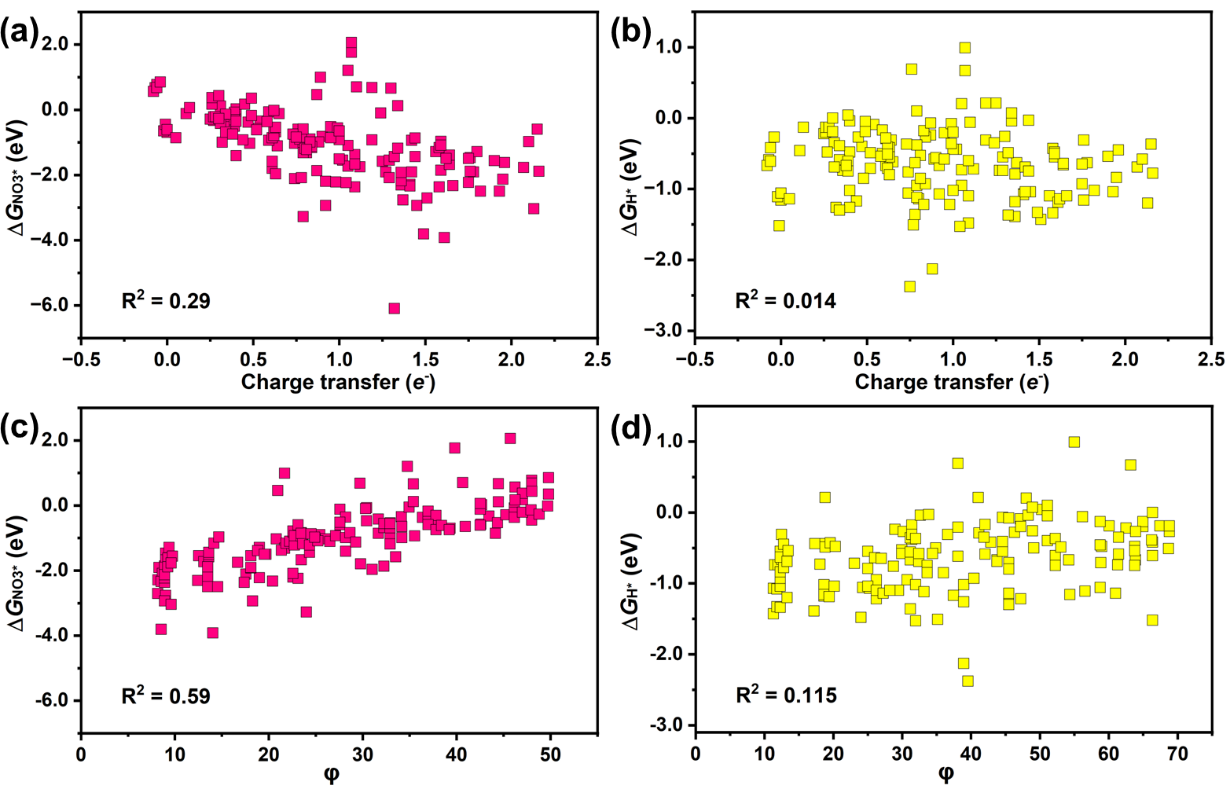


**Figure S18.** Scatter plot of adsorption free energy of (a) NO_3_^−^ and (b) H *versus* charge transfer *δ* for all TM-C_x_N_y_@BPN. Scatter plot of adsorption free energy of (c) NO_3_^−^ and (d) H *versus* descriptor 𝜑 for all TM-C_x_N_y_@BPN.


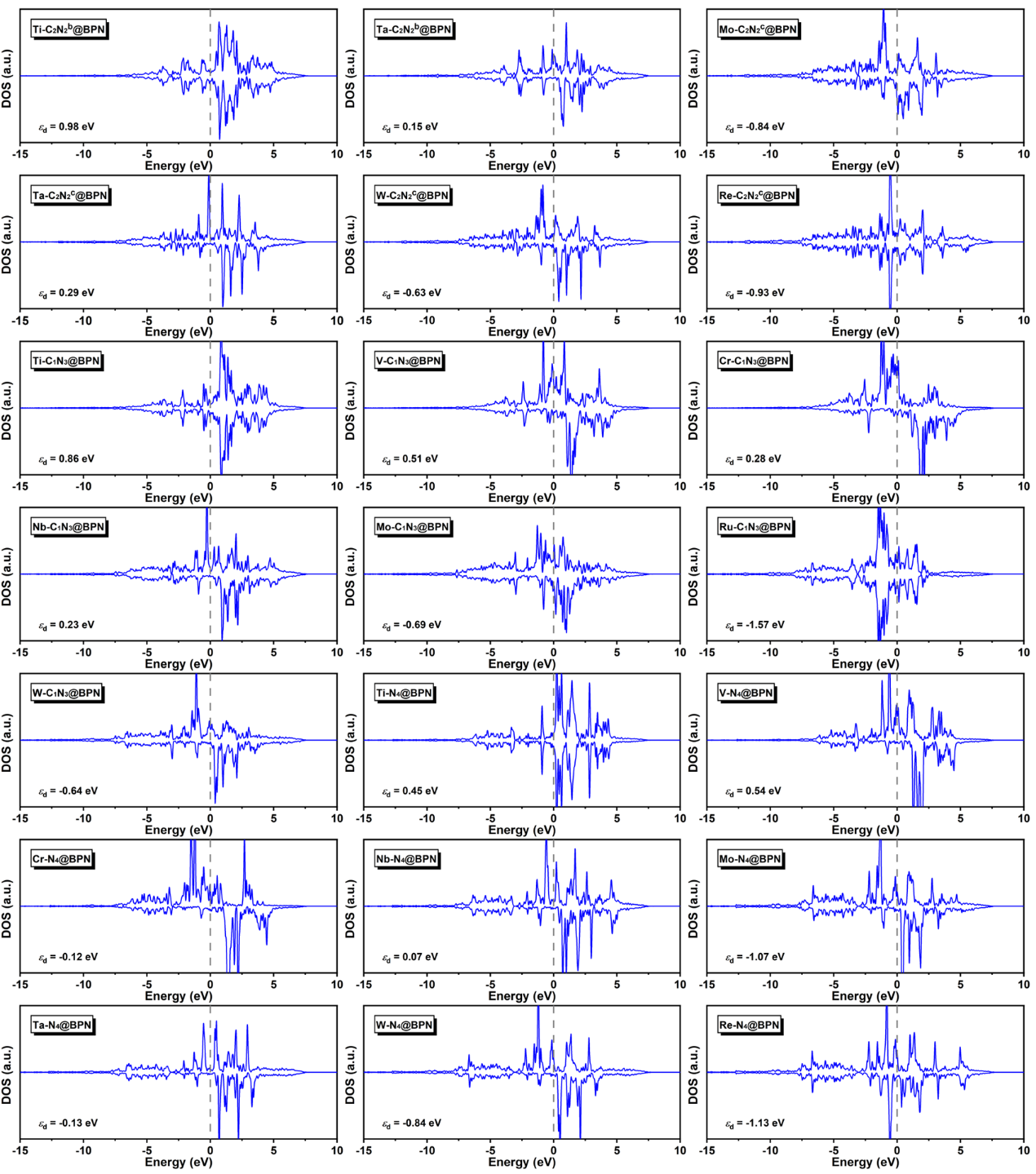


**Figure S19.** The projected density of states (PDOS) of TM-C_x_N_y_@BPN. The Fermi level was set to zero.


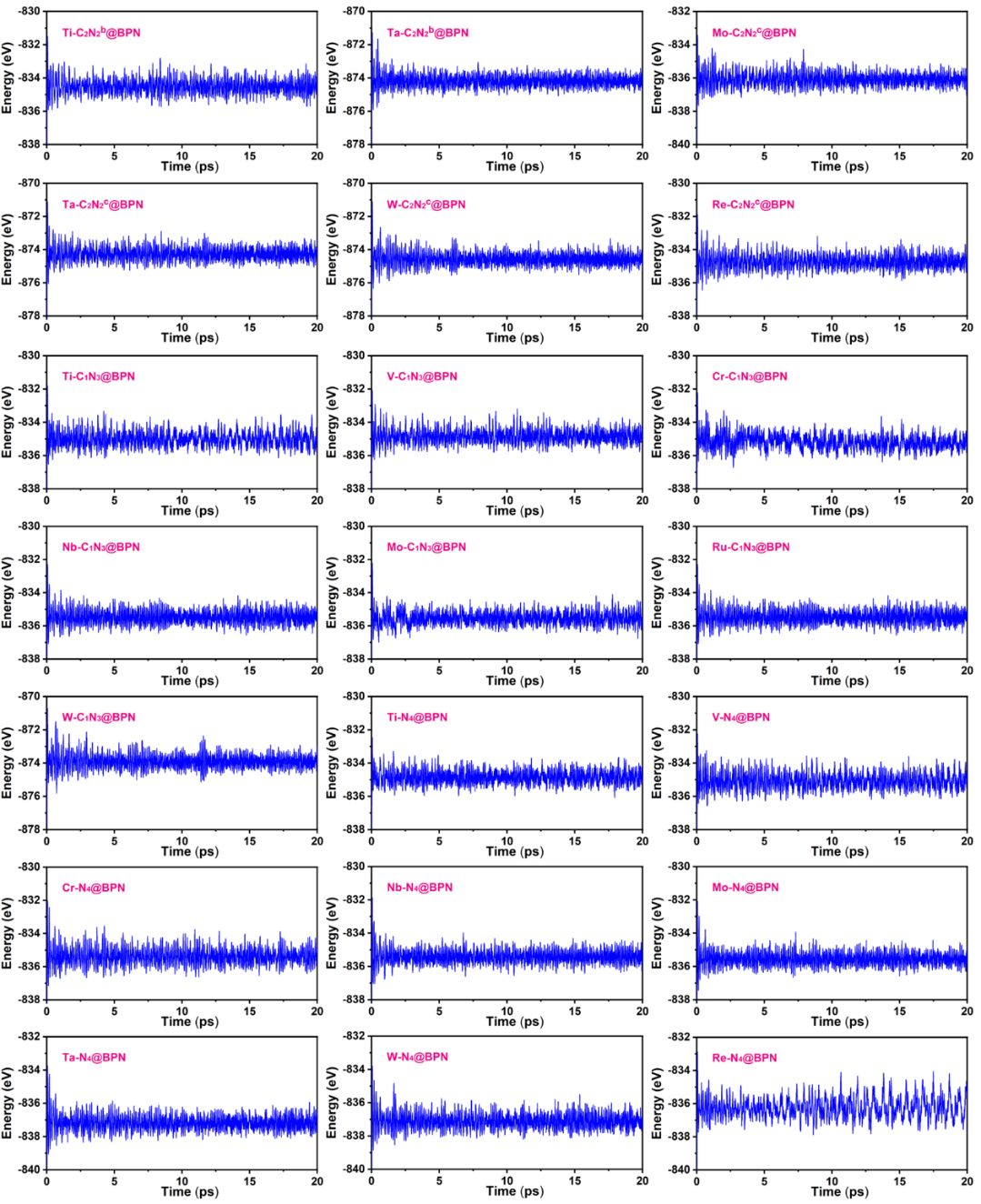


**Figure S20.** Fluctuations of energy with respect to time for TM-C_x_N_y_@BPN with *E*_f_ < 0 eV and *U*_diss_ > 0 and their corresponding structures after AIMD simulations at room temperature.


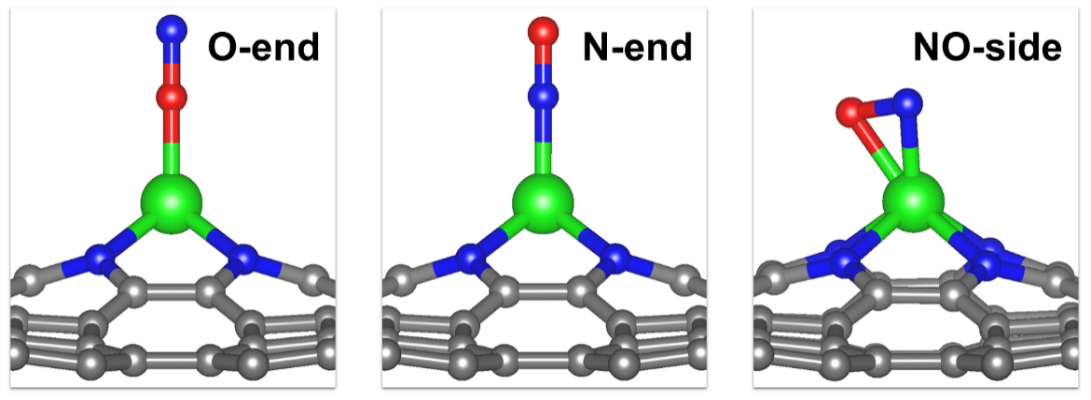


**Figure S21.** The O-end, N-end and NO-side configurations of NO adsorption. Nb-N_4_@BPN is taken as a representative sample.


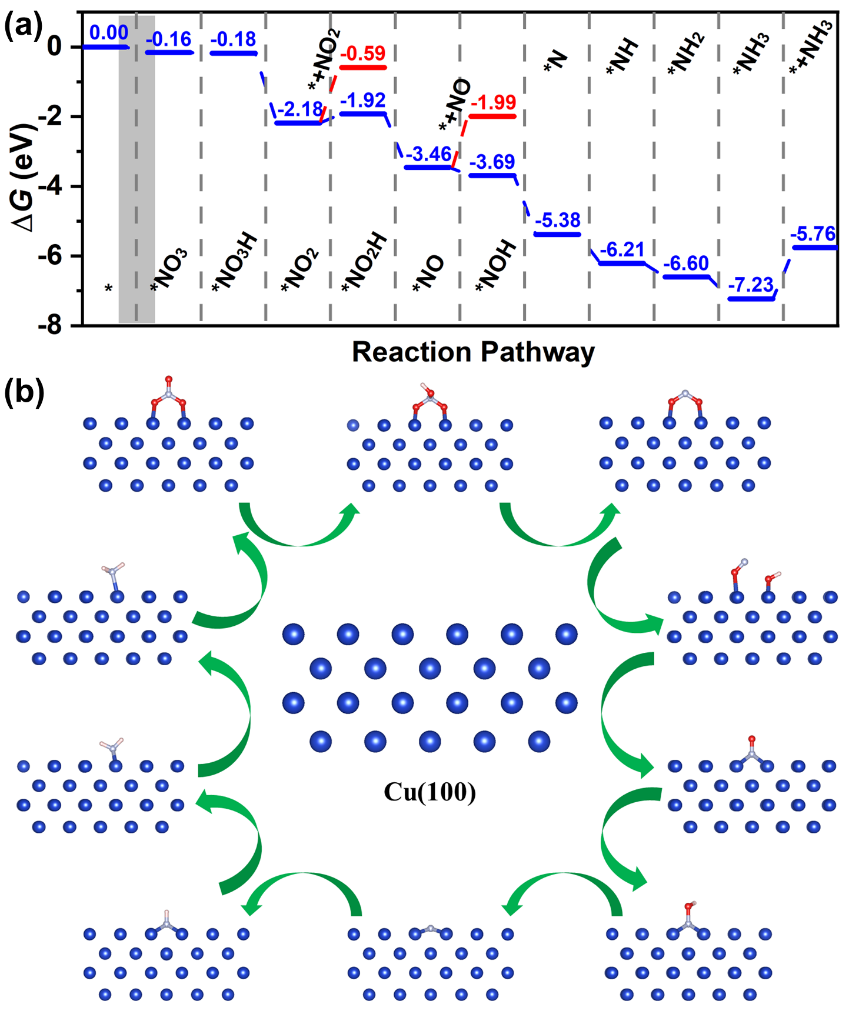


**Figure S22.** (a) The Gibbs free energy diagram for NO_3_RR on Cu(100) surface and (b) corresponding optimized configurations.


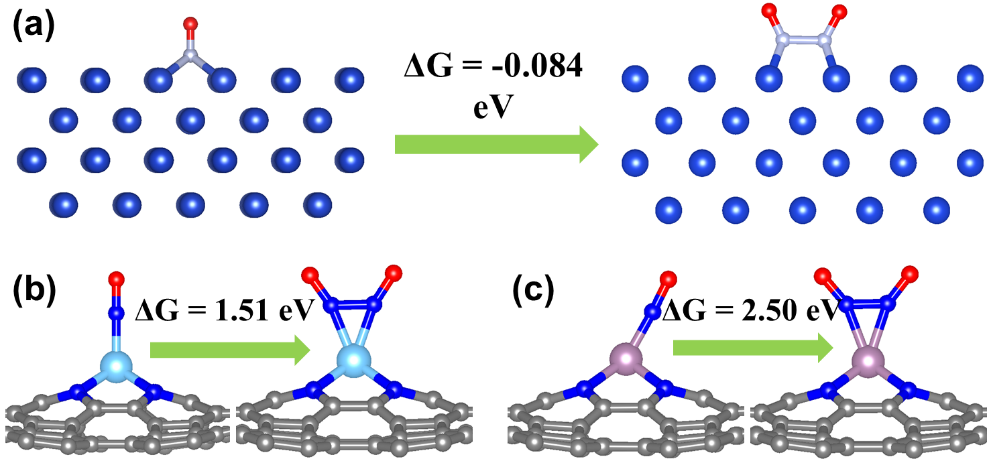


**Figure S23.** Gibbs free energy change of N-N coupling step for (a) Cu(100) surface, (b) Ti-N_4_@BPN, and (c) Mo-N_4_@BPN, respectively.


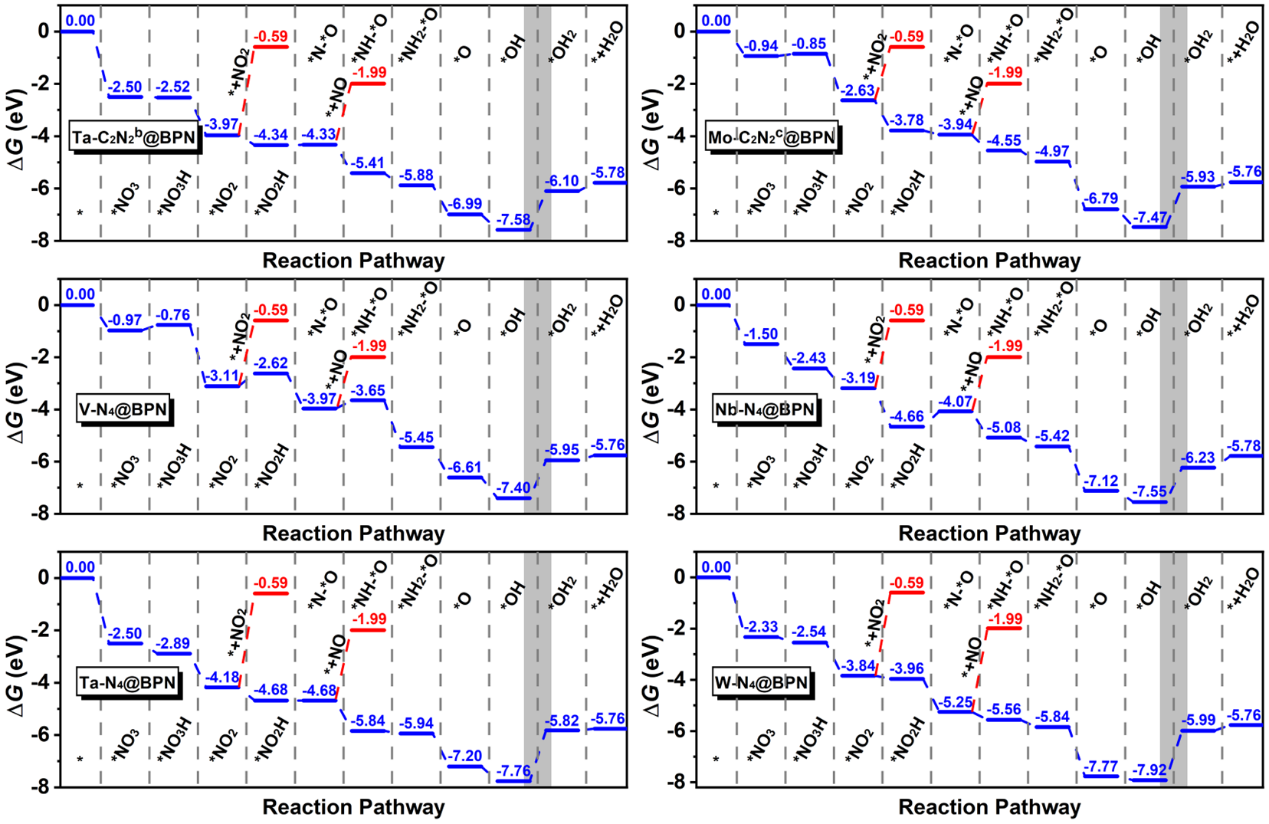


**Figure S24.** Gibbs free energy diagrams for the NO_3_RR on various TM-C*_x_*N*_y_*@BPN through the N-side pathway.


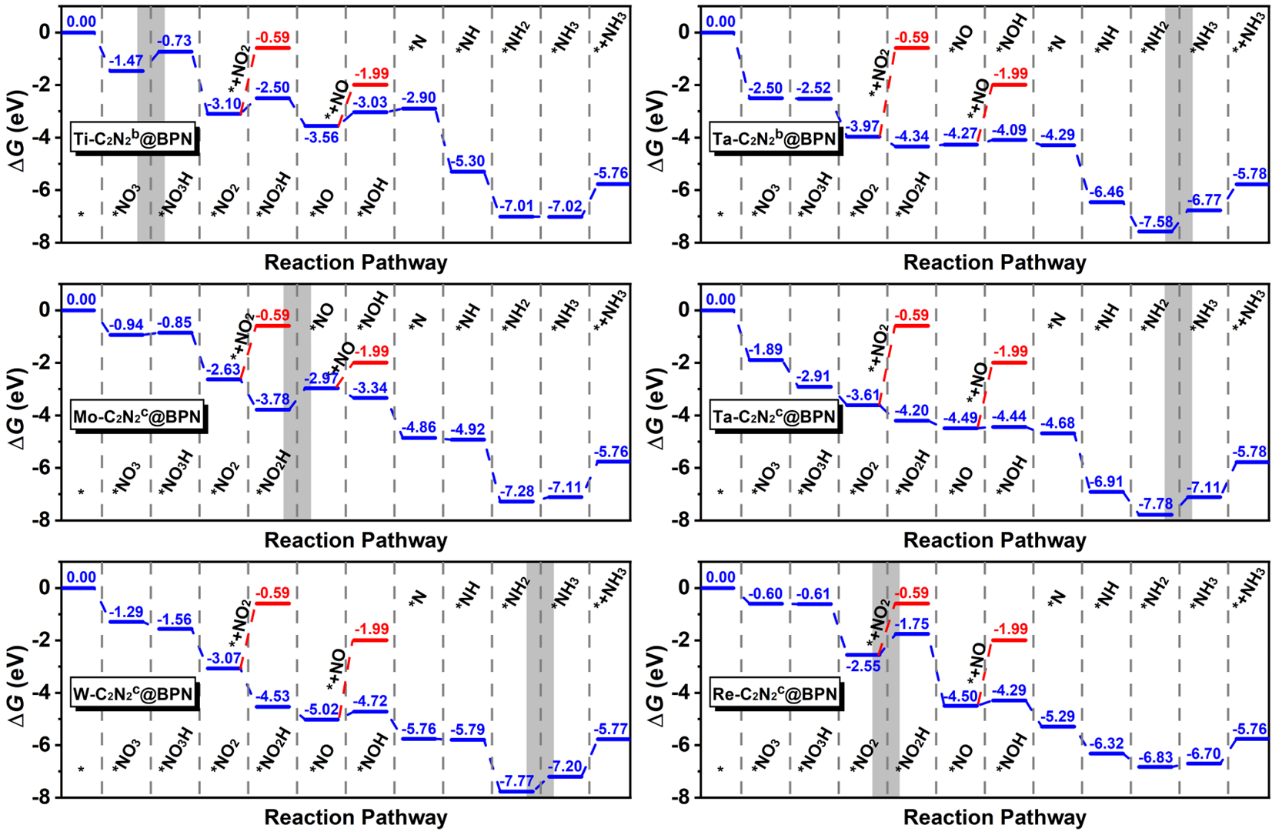


**Figure S25.** Gibbs free energy diagrams for the NO_3_RR on various TM-C_2_N_2_^b^@BPN and TM-C_2_N_2_^c^@BPN through the N-end pathway.


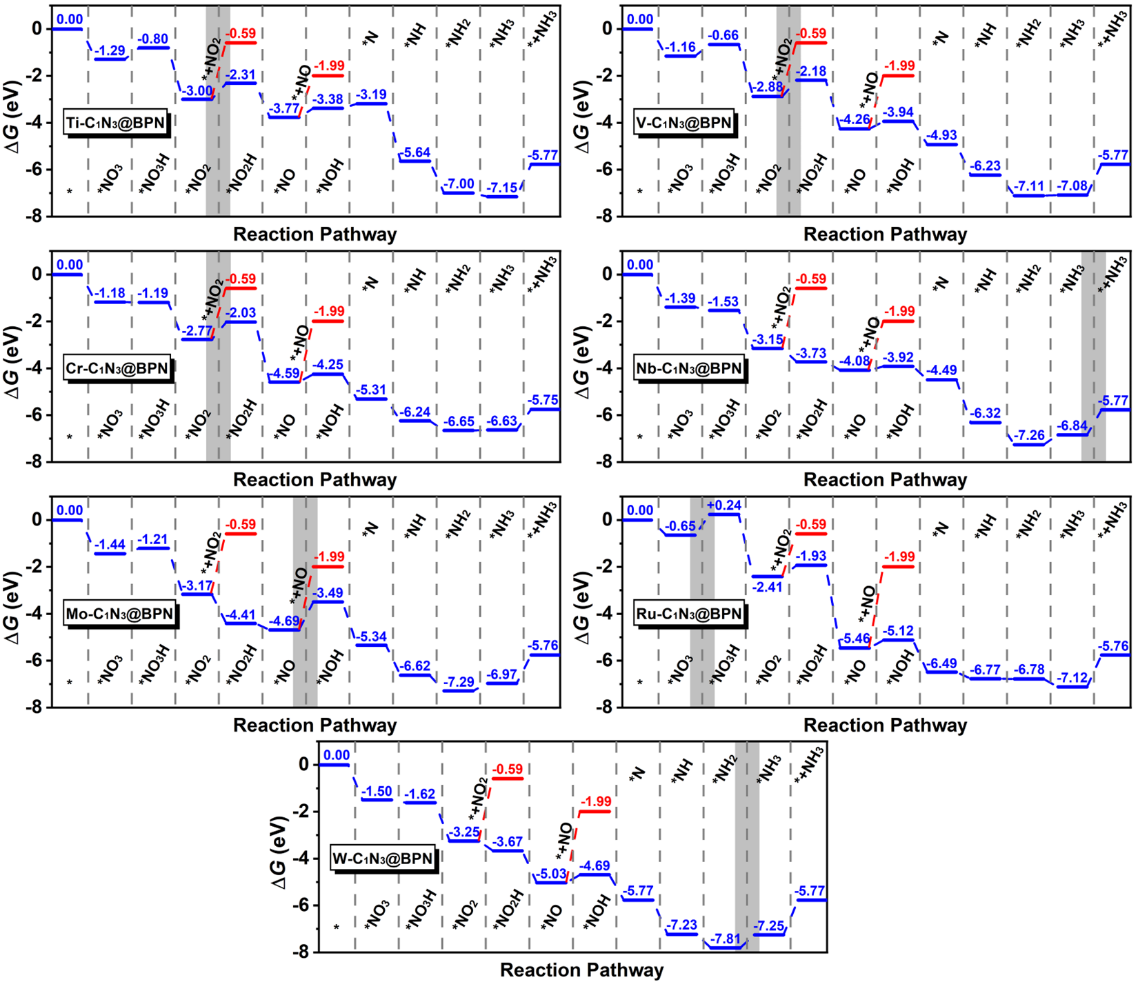


**Figure S26.** Gibbs free energy diagrams for the NO_3_RR on various TM-C_1_N_3_@BPN through the N-end pathway.


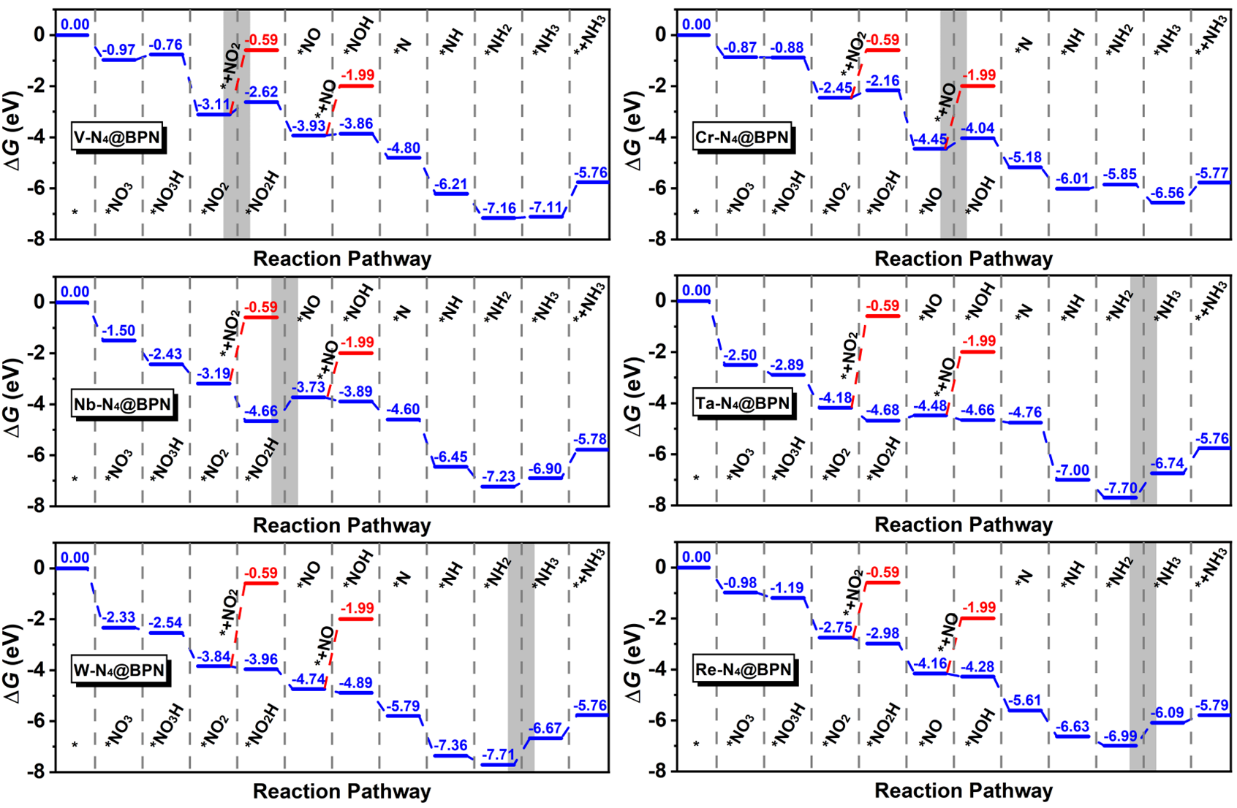


**Figure S27.** Gibbs free energy diagrams for the NO_3_RR on various TM-N_4_@BPN through the N-end pathway.


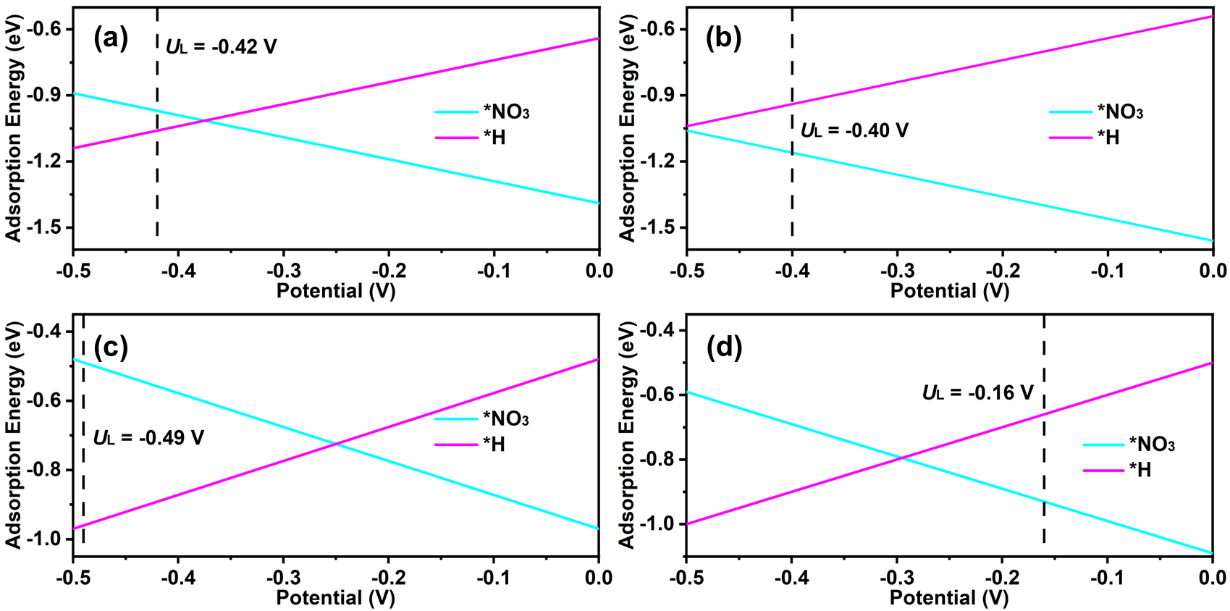


**Figure S28.** The adsorption energies of *NO_3_ and *H versus electrode potential from -0.5 to 0 V on (a) Nb-C_1_N_3_@BPN, (b) Ti-N_4_@BPN, (c) V-N_4_@BPN, and (d) Mo-N_4_@BPN respectively. The limiting potentials are labeled with dash lines.


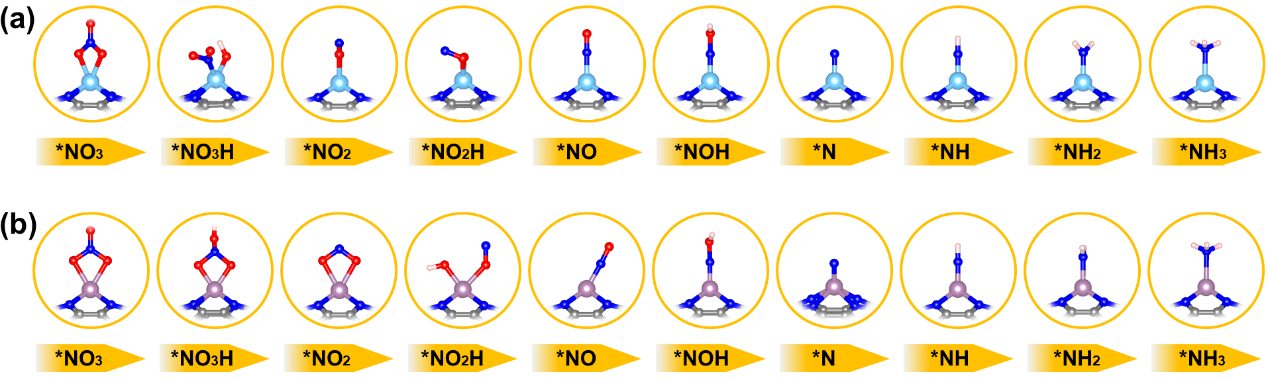


**Figure S29.** Optimized structures of NO_3_RR intermediates on (a) Ti-N_4_@BPN and (b) Mo-N_4_@BPN, respectively.


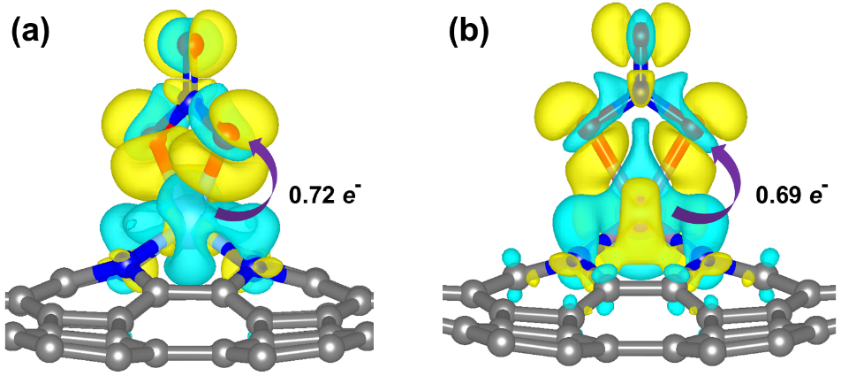


**Figure S30.** Iso-surface of charge density difference for NO_3_^−^ adsorption at (a) Ti-N_4_@BPN and (b) Mo-N_4_@BPN, respectively. The iso-surface level is 0.0015 e Å^−3^.


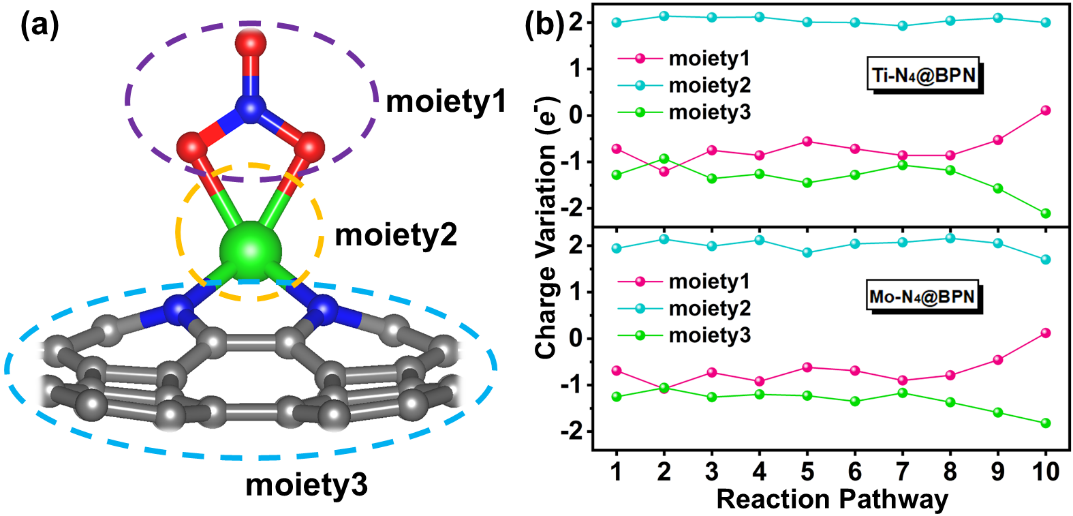


**Figure S31.** (a) Three moieties during NO_3_RR (moiety1: absorbed intermediates, moiety2: catalytic site; moiety3: BPN substrate). Nb-N_4_@BPN is taken as a representative sample. (b) Charge variation during the NO_3_RR on Ti-N_4_@BPN and Mo-N_4_@BPN.


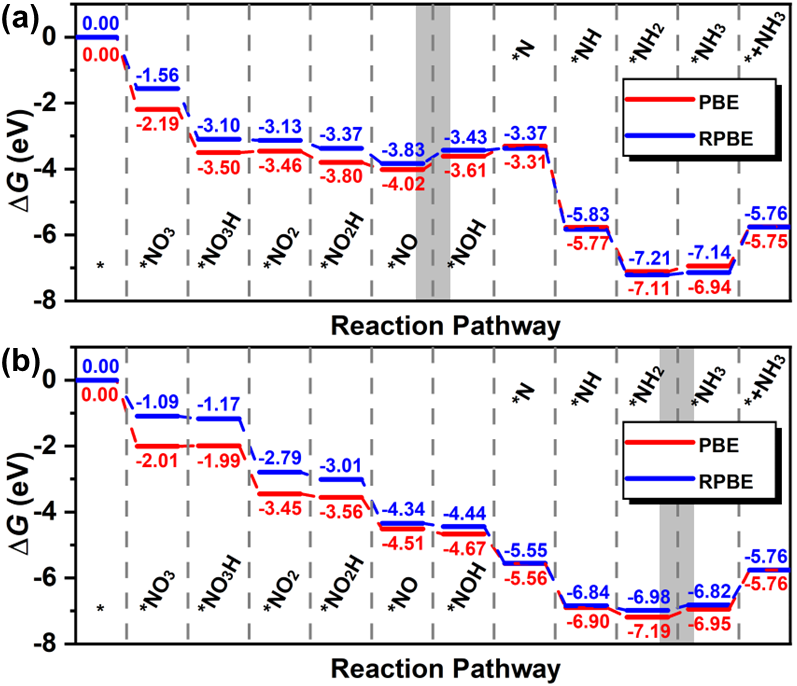


**Figure S32.** Comparison of NO_3_RR Gibbs free energy profiles for (a) Ti-N_4_@BPN and (b) Mo-N_4_@BPN using PBE and RPBE functionals.

**
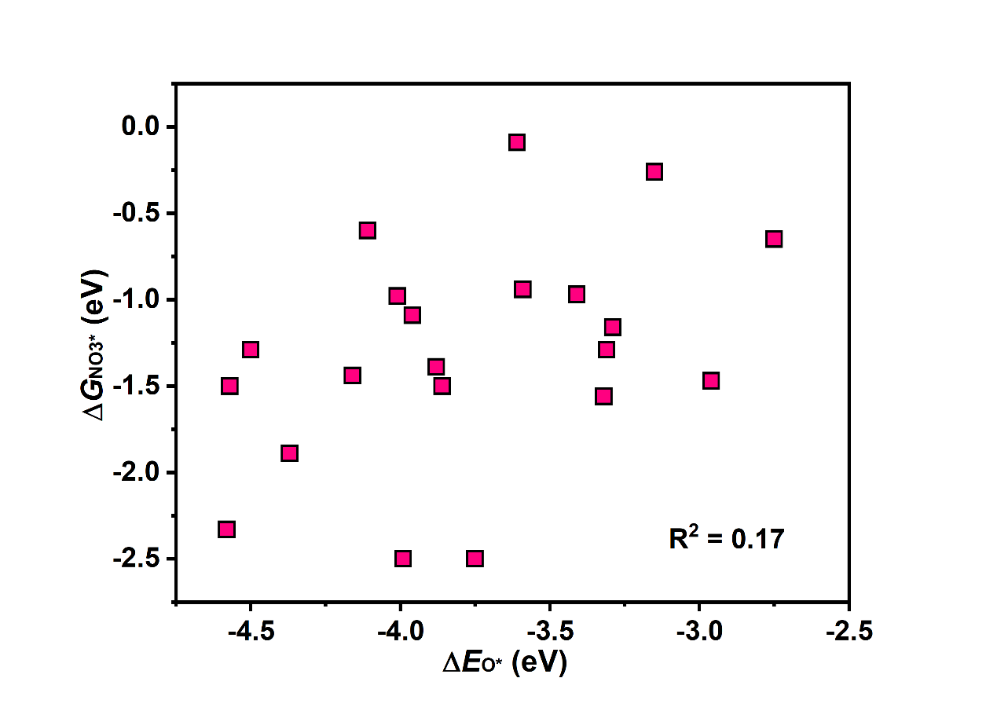
**

**Figure S33.** Relationships between the Gibbs free energy change of NO_3_^-^ adsorption and atomic O adsorption energy.


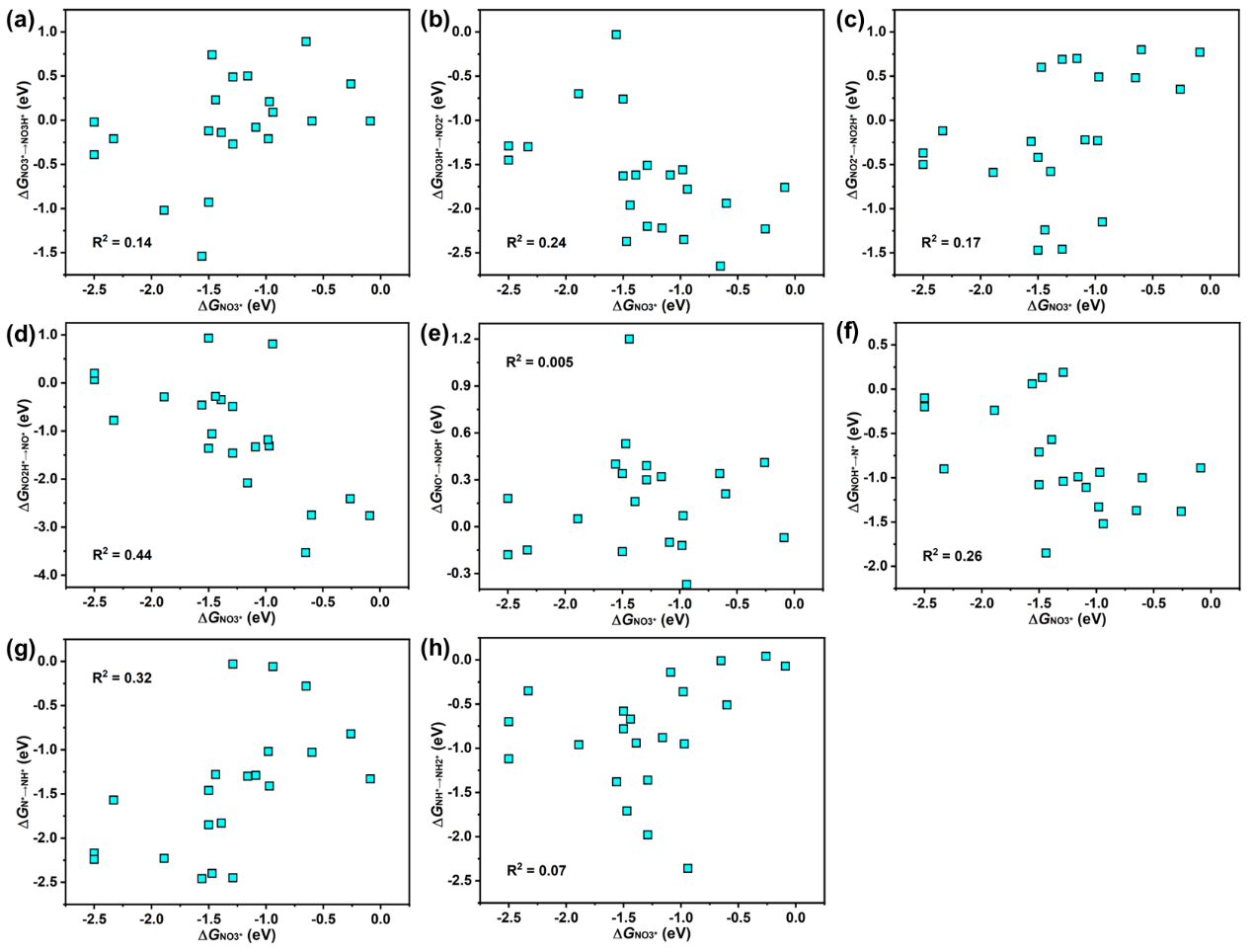


**Figure S34.** Relationships between the Gibbs free energy change of NO_3_^-^ adsorption and (a-h) each elementary reactions involved in NO_3_RR.


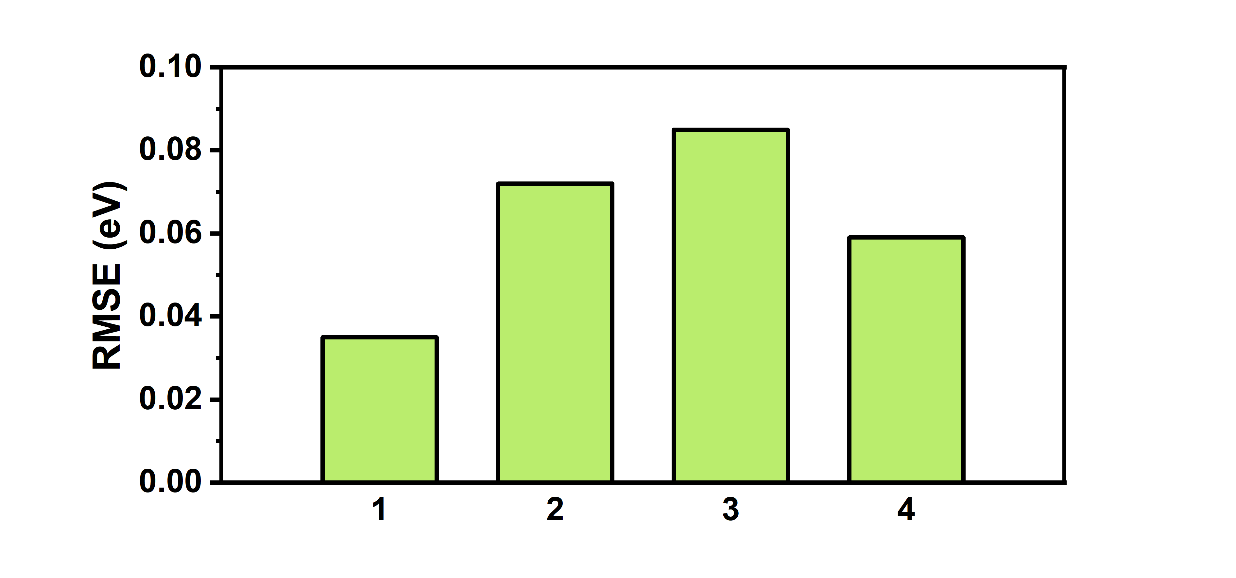


**Figure S35.** The root mean squared error (RMSE) of symbolic regression model using 4-fold cross-validation.

**References**

[1] G. Kresse, J. Furthmüller, *Comput. Mater. Sci.* **1996**, *6*, 15−50.

[2] G. Kresse, J. Furthmüller, *Phys. Rev. B* **1996**, *54*, 11169−11186.

[3] J. Hafner, *J. Comput. Chem.* **2008**, *29*, 2044−2078.

[4] P. E. Blochl, *Phys. Rev. B* **1994**, *50*, 17953−17979.

[5] J. P. Perdew, K. Burke, M. Ernzerhof, *Phys. Rev. Lett.* **1996**, *77*, 3865–3868.

[6] M. Ha, D. Y. Kim, M. Umer, V. Gladkikh, C. W. Myung, K. S. Kim, *Energy Environ. Sci.* **2021**, *14*, 3455−3468.

[7] S. Grimme, J. Antony, S. Ehrlich, H. Krieg, *J. Chem. Phys.* **2010**, *132*, 154104.

[8] W. Tang, E. Sanville, G. Henkelman, *J. Phys.: Condens. Matter* **2009**, *21*, 084204.

[9] H. Niu, Z. Zhang, X. Wang, X. Wan, C. Shao, Y. Guo, *Adv. Funct. Mater.* **2021**, *31*, 2008533.

[10] S. Wang, H. Gao, L. Li, K. S. Hui, D. A. Dinh, S. Wu, S. Kumar, F. Chen, Z. Shao, K. N. Hui, *Nano Energy* **2022**, *100*, 107571.

[11] X. Lv, T. Mou, J. Li, L. Kou, T. Frauenheim, *Adv. Funct. Mater.* **2022**, *32*, 2201262.

[12] S. Nosé, *J. Chem. Phys.* **1984**, *81*, 511–519.

[13] R. Nelson, C. Ertural, J. George, V. L. Deringer, G. Hautier, R. Dronskowski, *J. Comput. Chem.* **2020**, *41*, 1931−1940.

[14] J.-X. Liu, D. Richards, N. Singh, B. R. Goldsmith, *ACS Catal.* **2019**, *9*, 7052−7064.

[15] D. R. Lide, G. Baysinger, L. I. Berger, R. N. Goldberg, H. V. Kehiaian, K. Kuchitsu, D. L. Roth, D. Zwillinger, *CRC Handbook of Chemistry and Physics*; CRC Press: Boca Raton, **2005**.

[16] Z.-Y. Wu, M. Karamad, X. Yong, Q. Huang, D. A. Cullen, P. Zhu, C. Xia, Q. Xiao, M. Shakouri, F.-Y. Chen, J. Y. Kim, Y. Xia, K. Heck, Y. Hu, M. S. Wong, Q. Li, I. Gates, S. Siahrostami, H. Wang, *Nat. Commun.* **2021**, *12*, 2870.

[17] Y. Wang, M. Shao, *ACS Catal.* **2022**, *12*, 5407−5415.

[18] F. Rehman, S. Kwon, C. B. Musgrave III, M. Tamtaji, W. A. Goddard III, Z. Luo, *Nano Energy* **2022**, *103B*, 107866.

[19] Z. Shu, H. Chen, X. Liu, H. Jia, H. Yan, Y. Cai, *Adv. Funct. Mater.* **2023**, *33*, 2301493.

[20] R. Zhu, Y. Qin, T. Wu, S. Ding, Y. Su, *Small* **2024**, *20*, 2307315.

[21] Y. Pang, Z. Ding, A. Ma, G. Fan, H. Xu, *Sep. Purif. Technol.* **2025**, *354*, 129422.

[22] J. K. Nørskov, J. Rossmeisl, A. Logadottir, L. Lindqvist, J. R. Kitchin, T. Bligaard, H. Jónsson, *J. Phys. Chem. B* **2004**, *108*, 17886−17892.

[23] A. A. Peterson, F. Abild-Pedersen, F. Studt, J. Rossmeisl, J. K. Nørskov, *Energy Environ. Sci.* **2010**, *3*, 1311−1315.

[24] F. Pedregosa, G. Varoquaux, A. Gramfort, V. Michel, B. Thirion, O. Grisel, M. Blondel, P. Prettenhofer, R. Weiss, V. Dubourg, J. Vanderplas, A. Passos, D. Cournapeau, M. Brucher, M. Perrot, É. Duchesnay, *J. Mach. Learn. Res.* **2011**, *12*, 2825−2830.

[25] M. Yang, Z. Wang, D. Jiao, G. Li, Q. Cai, J. Zhao, *Appl. Surf. Sci.* **2022**, *592*, 153213.

[26] Z. Ding, Y. Pang, A. Ma, Z. Liu, Z. Wang, G. Fan, H. Xu, *Int. J. Hydrogen Energy* **2024**, *80*, 586–598.

[27] L. Lv, Y. Shen, J. Liu, X. Meng, X. Gao, M. Zhou, Y. Zhang, D. Gong, Y. Zheng, Z. Zhou, *J. Phys. Chem. Lett.* **2021**, *12*, 11143–11150.

[28] L. Lv, Y. Shen, J. Liu, X. Gao, M. Zhou, Y. Zhang, X. Meng, X. Yang, D. Gong, Y. Zheng, *Appl. Catal., A* **2022**, **645**, 118846.

[29] S. Zhu, M. Qin, L. Chen, S. Jiang, Y. Zhou, J. Jiang, W. Zhang, *J. Phys. Chem. Lett.* **2023**, *14*, 4185–4191.

[30] J. Wu, J.-H. Li, Y.-X. Yu, *J. Phys. Chem. Lett.* **2021**, *12*, 3968-3975.

[31] H. Ma, S. Yuan, S. Li, J. Wang, H. Zhang, Y. Zhang, J. Yang, J. Sun, K. Xu, S. Lei,  *J. Phys. Chem. C* **2025**, *129*, 1953-1960.

[32] R. Hu, H. Wang, R. Zhu, X. Yang, X. Zhao, F. Ma, J. Yu, X. Jiang, *J. Energy Chem.* **2025**, *110*, 336–346.
